# Regioselective Functionalization of Quinolines through C-H Activation: A Comprehensive Review

**DOI:** 10.3390/molecules26185467

**Published:** 2021-09-08

**Authors:** Alessandra Corio, Christine Gravier-Pelletier, Patricia Busca

**Affiliations:** Laboratoire de Chimie et Biochimie Pharmacologiques et Toxicologiques, UMR CNRS 8601, Université de Paris, 45 rue des Saints-Pères, 75006 Paris, France; alessandra.corio@u-paris.fr (A.C.); christine.gravier-pelletier@u-paris.fr (C.G.-P.)

**Keywords:** quinoline, C-H activation, selective functionalization, metal catalyzed reactions

## Abstract

Quinoline is a versatile heterocycle that is part of numerous natural products and countless drugs. During the last decades, this scaffold also became widely used as ligand in organometallic catalysis. Therefore, access to functionalized quinolines is of great importance and continuous efforts have been made to develop efficient and regioselective synthetic methods. In this regard, C-H functionalization through transition metal catalysis, which is nowadays the Graal of organic green chemistry, represents the most attractive strategy. We aim herein at providing a comprehensive review of methods that allow site-selective metal-catalyzed C-H functionalization of quinolines, or their quinoline *N*-oxides counterparts, with a specific focus on their scope and limitations, as well as mechanistic aspects if that accounts for the selectivity.

## 1. Introduction

**Figure 1:**
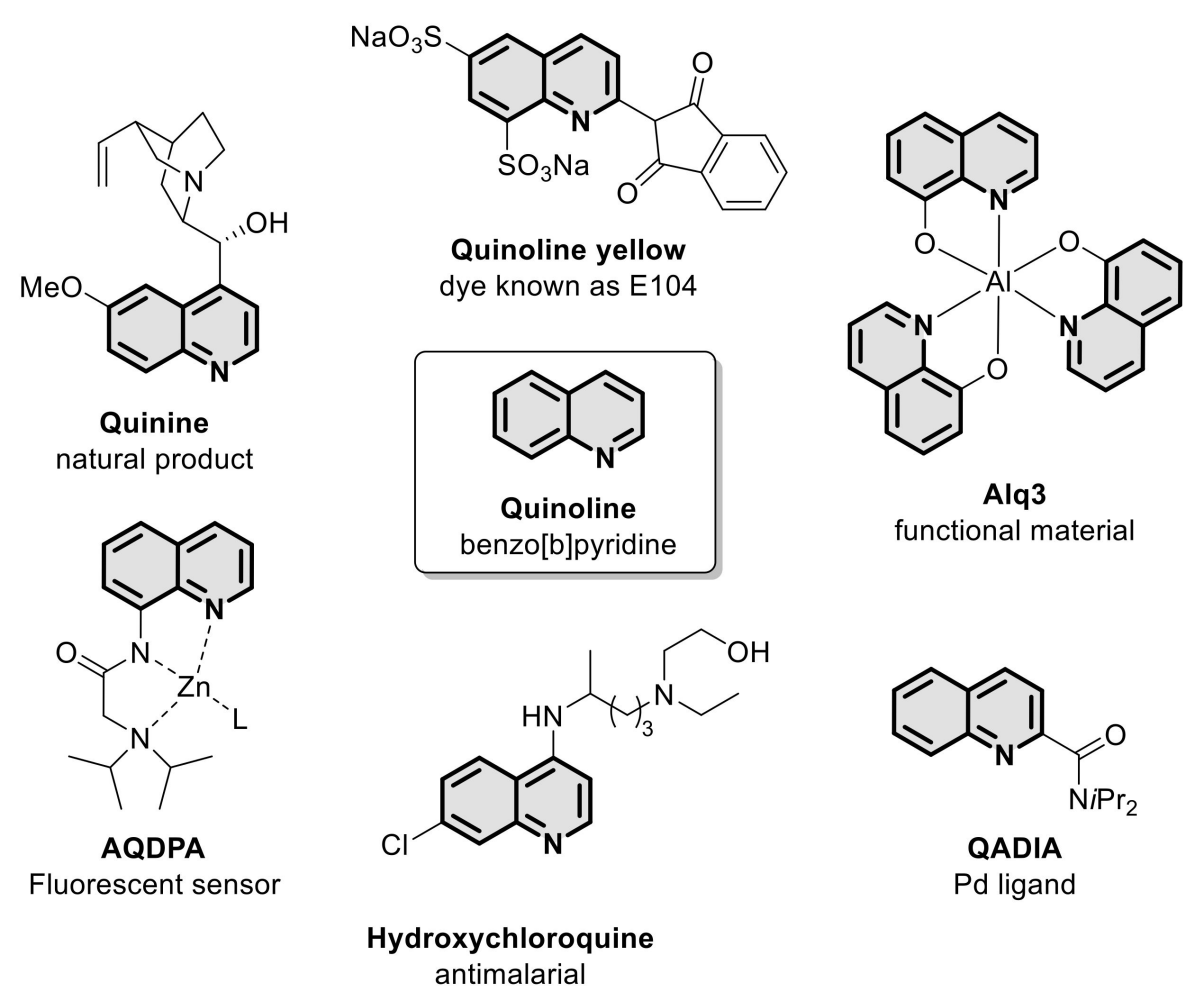
Quinoline scaffold and illustrative examples of significant quinoline derivatives.

Quinoline, also known as benzo[b]pyridine, is a remarkable heteroaromatic scaffold that belongs to the category of the privileged structures [1,2,3]. This structural motif can indeed be found in numerous natural products [4,5,6,7,8,9,10,11] and in countless drugs or pharmaceuticals with biological spectra ranging from antimalarial to anticancer activities [12,13,14,15,16,17,18,19,20,21,22,23]. Furthermore, it is also widely used as ligand in organometallic catalysis [24,25,26,27,28,29,30,31] as well as in material chemistry [32,33,34,35,36,37,38,39,40]. A few representative examples are depicted in Figure 1.

**Scheme 1:**
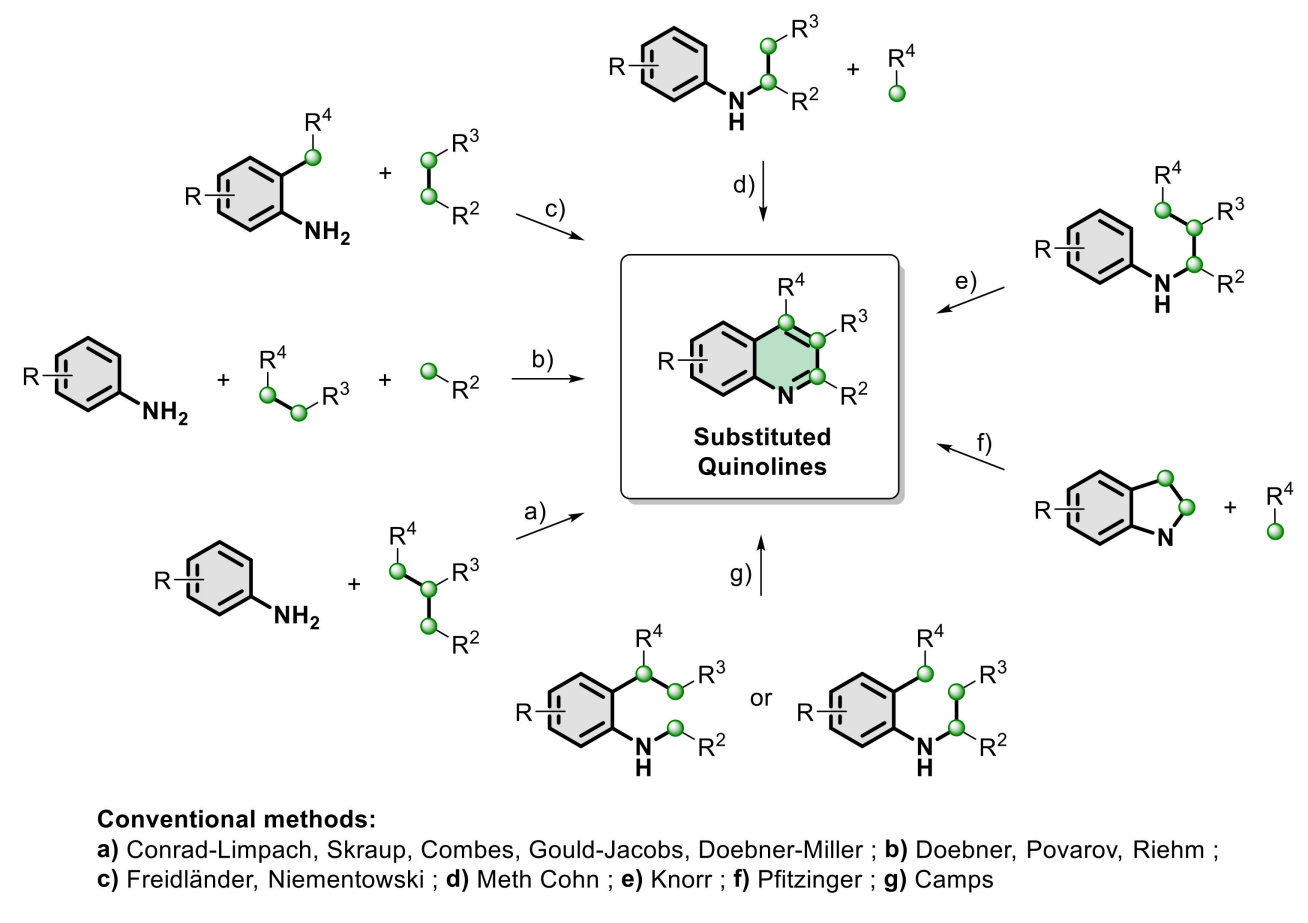
Main strategies to access quinoline derivatives through conventional methods.

Considering the significance of quinolines, intensive research efforts have been made to design efficient synthetic strategies. The main methods developed during the last century aimed at the construction of the pyridine ring through the cyclocondensation or cyclization of anilines derivatives with carbonylated reagents, as summarized in Scheme 1 [41,42,43,44,45,46]. Widely used, some of these procedures became well-known name reactions such as Combes, Friëdlander or Knorr reactions, to quote but a few.

However, these conventional quinolines syntheses suffer from several drawbacks such as harsh conditions, limited substrate scope, poor functional group tolerance, long reaction time, toxic solvents and low yields. In the last decades, tremendous work has been achieved to develop cleaner and safer pathways for their synthesis [47,48,49,50,51,52,53,54,55,56,57,58,59,60] and functionalization [61,62,63,64,65,66,67,68]. This need for greener conditions meets the C-H activation concept that has just revolutionized the field of organic synthesis [69,70,71,72,73,74,75]. This new type of catalytic process allows both atom and step economy as no pre-functionalized precursor is required. Furthermore, these non-demanding reactions usually tolerate various functional groups and thus allow a broad range of compounds to be used as substrates. The development of this promising synthetic tool led to highly efficient methods that can be successfully applied to the preparation or functionalization of diverse arenes and heteroarenes using various metal-based catalysts [76,77,78,79,80,81,82,83,84,85,86,87,88,89,90,91,92,93,94,95].

Since the pioneering work of Lewis, Bergman and Ellman regarding the Rh-catalyzed alkylation of quinoline at position C2 in 2007 [96], nearly one hundred methods have been published. They have already been partly reviewed, including: site-selective C-H functionalization of quinolines beyond C2 selectivity [97], C-H functionalization of the distal positions in quinolines [98], remote C5-H functionalization of 8-aminoquinolinamides [99], or C2-H alkenylation of quinoline *N*-oxides [100], the most recent one being the Rh-catalyzed functionalization of quinolines [101]. The development of methods focusing on the regioselective direct C-H functionalization of quinoline derivatives by transition metal is of high importance to achieve atom- and step-economical synthesis of libraries of quinoline-containing compounds that could not be obtained otherwise with conventional methods.

**Scheme 2:**
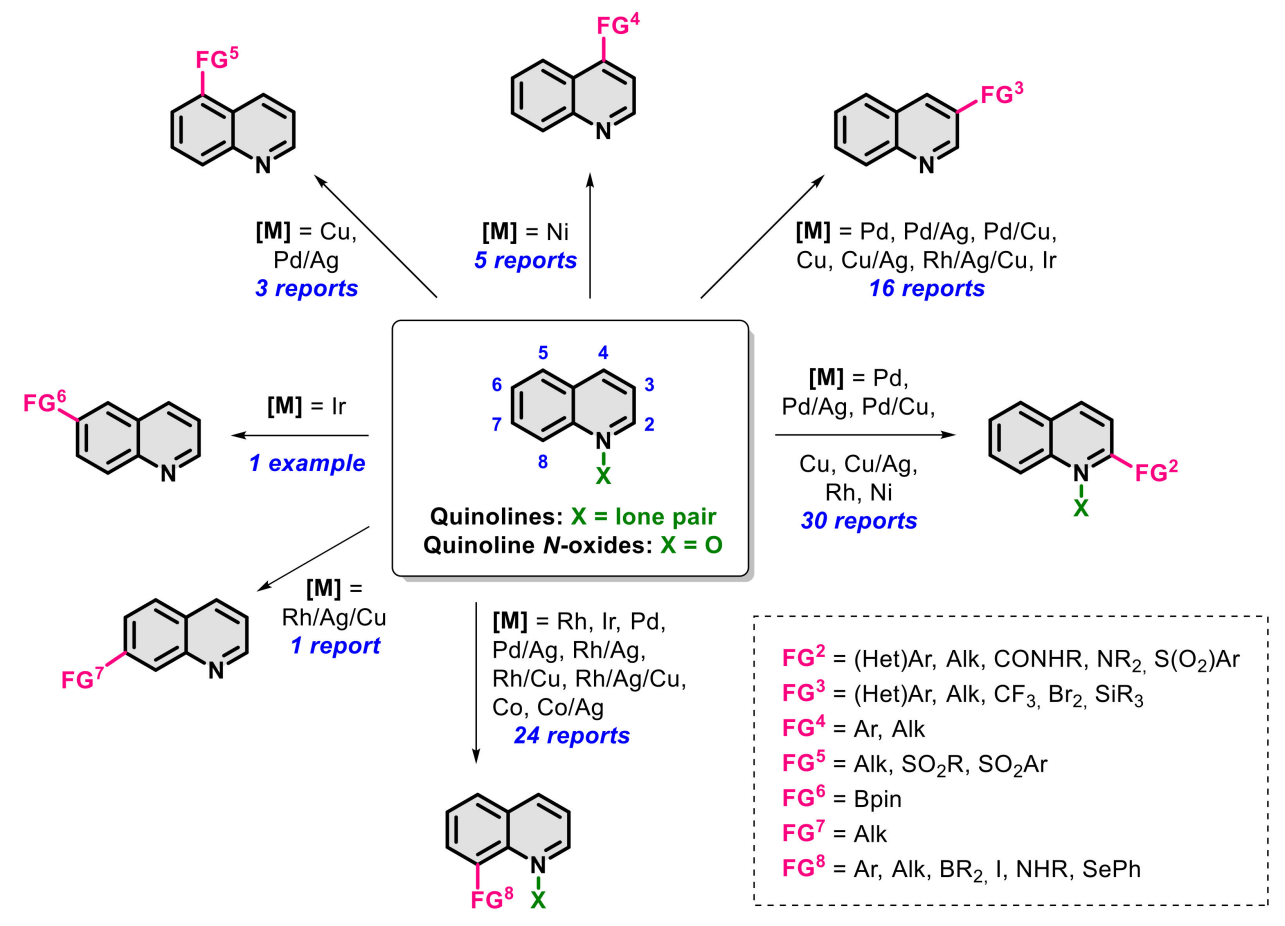
Scope of regioselective C-H functionalization of quinolines or quinoline *N*-oxides.

The aim of our review is to provide a comprehensive and updated overview of all these impressive research advances that i) enable the site-selective functionalization of quinolines at almost all positions and ii) allow the introduction of a large variety of functional groups, as represented in Scheme 2.

As above mentioned in Scheme 2, C-H functionalization of quinolines can be achieved using several of the main transition metals such as rhodium, iridium, palladium, copper, or combinations thereof, and thus various and complex metal-dependent mechanisms are involved. Besides being too numerous to be detailed herein, the mechanistic evidences are not systematically disclosed by the authors and consequently the full details of each mechanism will not be discussed in this review. Nevertheless, we do think that providing the reader with valuable insights regarding the general mechanism along with the key intermediates that account for regioselectivity can be helpful to functionalize new substrates or design new methods. If more details about the full mechanism are needed, the reader is invited to refer to the original papers that are presented herein.

**Scheme 3:**
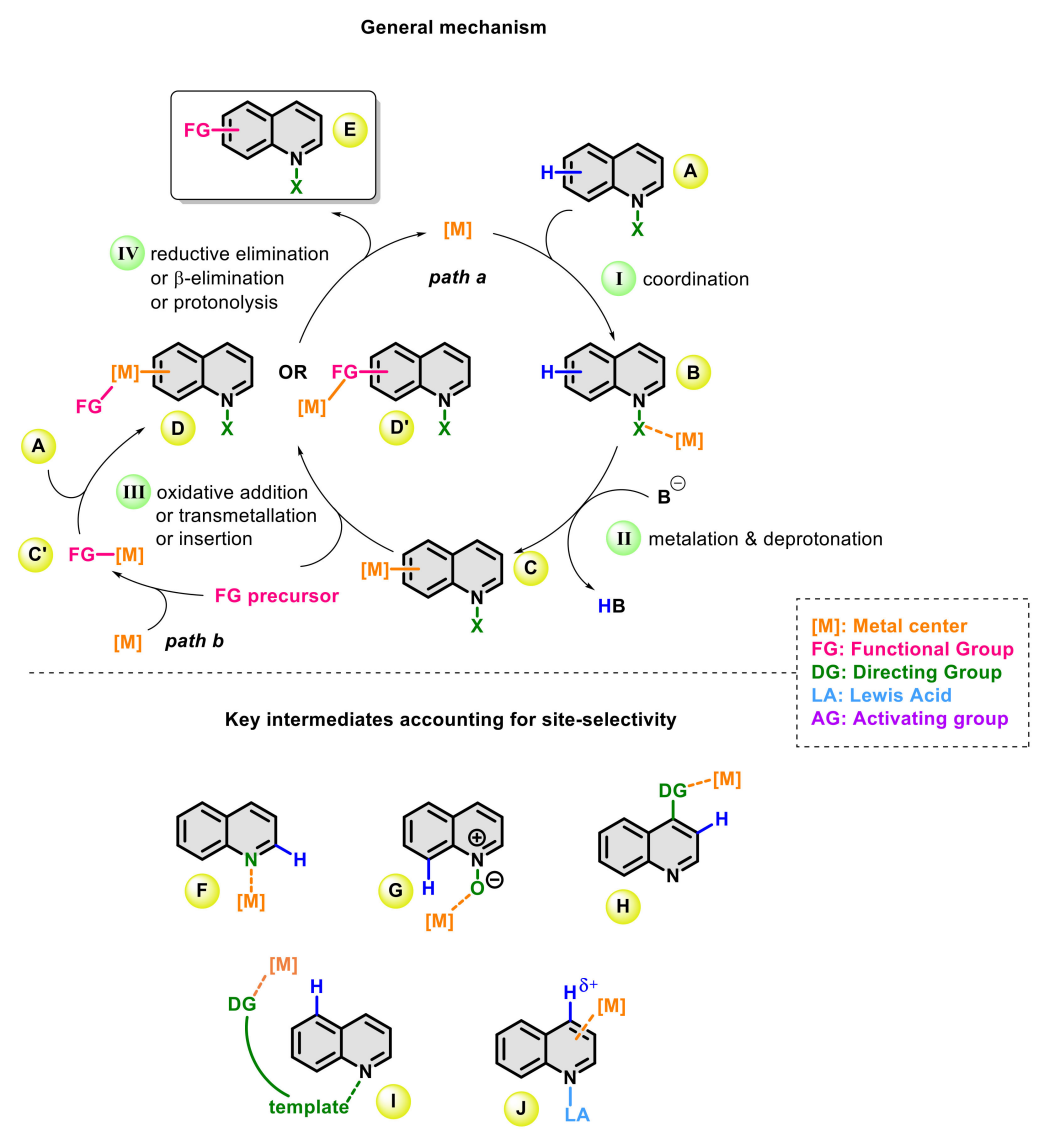
C-H functionalization general mechanism and key intermediates.

The Scheme 3 summarizes the common steps shared by virtually all the mechanisms, whatever the metal being used, and will serve as a reference for mechanistic discussions in the following paragraphs.

The first step consists in the coordination of the metal with the quinoline (QN) or the quinoline *N*-oxide (QNO) **A**, step **I**, to form the complex **B**. Subsequent C-H activation in step **II**, that corresponds to metalation and deprotonation, provides the organometallic species **C**. This step can be achieved through various mechanisms, such as oxidative addition (OA), single-electron transfer (SET), concerted metalation-deprotonation (CMD), sigma-metathesis or electrophilic aromatic substitution depending on the nature of the metal center. Introduction of the functional group (FG) on the metallic center that occurs in step **III**, can be achieved through oxidative addition, transmetalation or metal insertion. FG precursors can be for instance halides, boronic acids, Grignard reagents or non-functionalized arenes in the particular case of a double C-H activation. The resulting intermediate **D** or **D′** leads in step **IV** to the desired product **E** through reductive elimination, β-elimination or protonolysis, which allow also the regeneration of the catalyst. This latter step can sometimes require the assistance of an oxidant.

When the substrate possesses several C-H bonds, the C-H activation is usually driven by the reactivity of these C-H bonds: the more acidic a proton is, the easier will be the formation of the organometallic intermediate. This rule can be circumvented by the use of a directing group (DG) that is able to bring the metal in front of a specific position [98,102,103,104,105,106]. In the specific case of quinoline, the vast majority of C-H functionalizations were described in position 2 or 8 because the nitrogen atom of the pyridine ring, or the oxygen atom of the corresponding *N*-oxide, can act as embedded DG (**F** and **G**, Scheme 3). In contrast, only a few examples are reported to target other positions because the challenging control of site-selectivity requires appropriate molecular tricks that can be for instance: i) the choice of bulky ligands to favor the metalation in position 3 thanks to the trans effect [107], ii) the introduction of a non-removable DG (NR-DG) to reach any distal positions (**H**, Scheme 3), iii) the use of a template that acts as a transient or traceless DG (TDG) that binds to the nitrogen of the pyridine ring and reach position 5 or 3 (**I**, Scheme 3), and iv) the use of Lewis Acid (LA) to enhance the polarization of the C-H bond (**J**, Scheme 3).

Taken together, all these approaches contribute, even though not at the same level, to the expansion of the preparative methods that can afford substituted quinoline in a regioselective manner. In order to present this review as a handbook of C-H activation toolbox for site-selective modification of QNs, sections are categorized depending on the position of functionalization and whenever possible, further subdivided according to the nature of the functional group.

## 2. Functionalization in Position 2

**Scheme 4:**
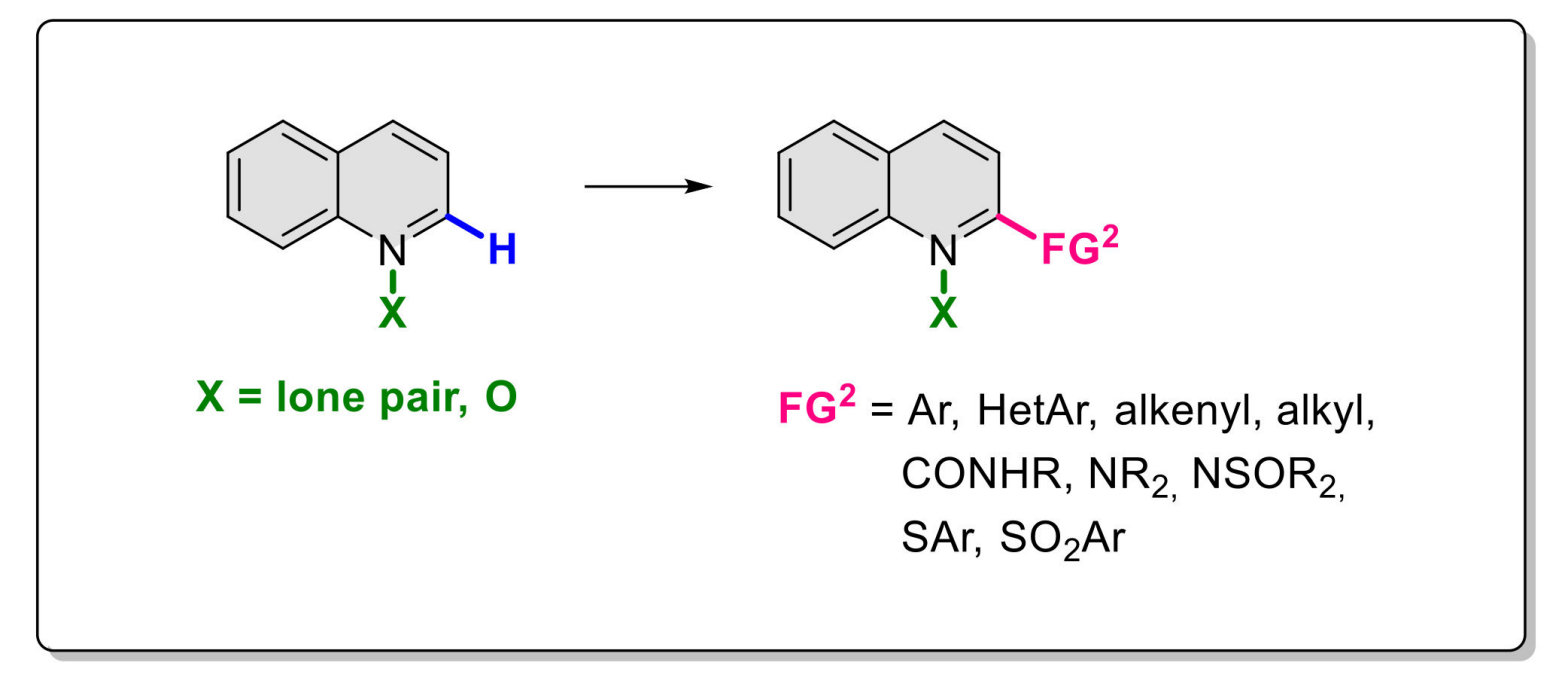
Scope of the selective C-H functionalization achieved in position 2 to date.

Thanks to the tremendous piece of work accomplished over the past decade, more than thirty different methods were successfully developed to introduce a large variety of aryl, heteroaryl, alkenyl, alkyl, acyl, amino, sulfonamido, thio, arylsulfonyl groups in position 2 (Scheme 4). The vast majority of these reactions are using quinoline *N*-oxides as substrates, and Pd appears to be the metal of choice to form C-C bonds, while Cu-based catalysts are preferred in the case of C-N and C-S bonds.

### 2.1. Formation of C-Ar Bonds

**Scheme 5:**
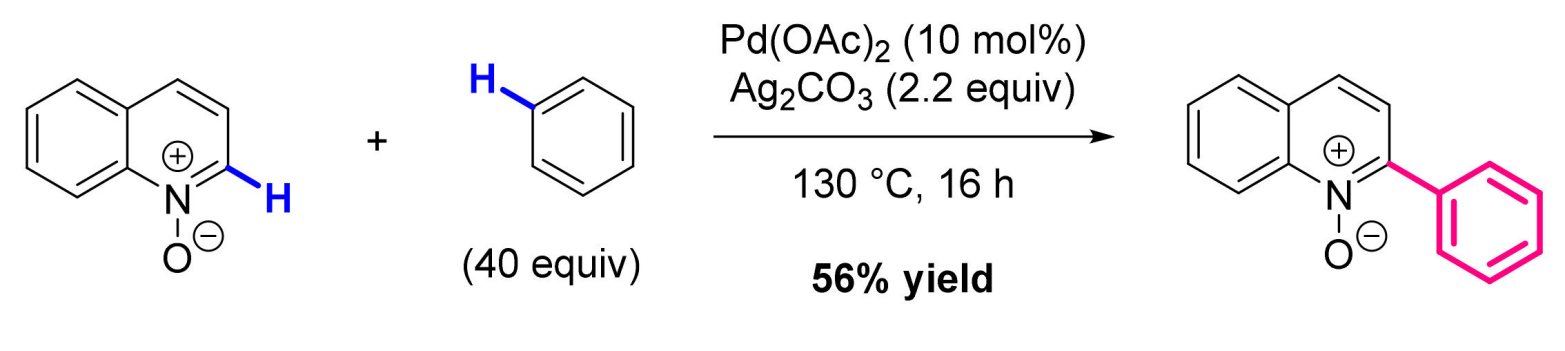
Pd-catalyzed C2 arylation of quinoline-*N*-oxide with unactivated benzene.

The first example of arylation dates back to 2008, when Chang et al. were studying the Pd(II)-catalyzed alkenylation and arylation of pyridine *N*-oxides in *ortho* position via a double C-H bond activation route [108]. The arylation, at first observed as a side reaction, was performed with 1 equiv pyridine *N*-oxide and 40 equiv of benzene in the presence of Pd(OAc)_2_ (10 mol%) and Ag_2_CO_3_ (2.2 equiv) at 130 °C, but failed to be improved by the addition of various additives. In these conditions, exploration of the scope revealed that quinoline *N*-oxide can be arylated in position 2 with a good regioselectivity in 56% yield (Scheme 5).

Some kinetic isotope competition experiments were carried out using deuterated pyridine *N*-oxide, but no mechanism was proposed at this stage.

**Scheme 6:**
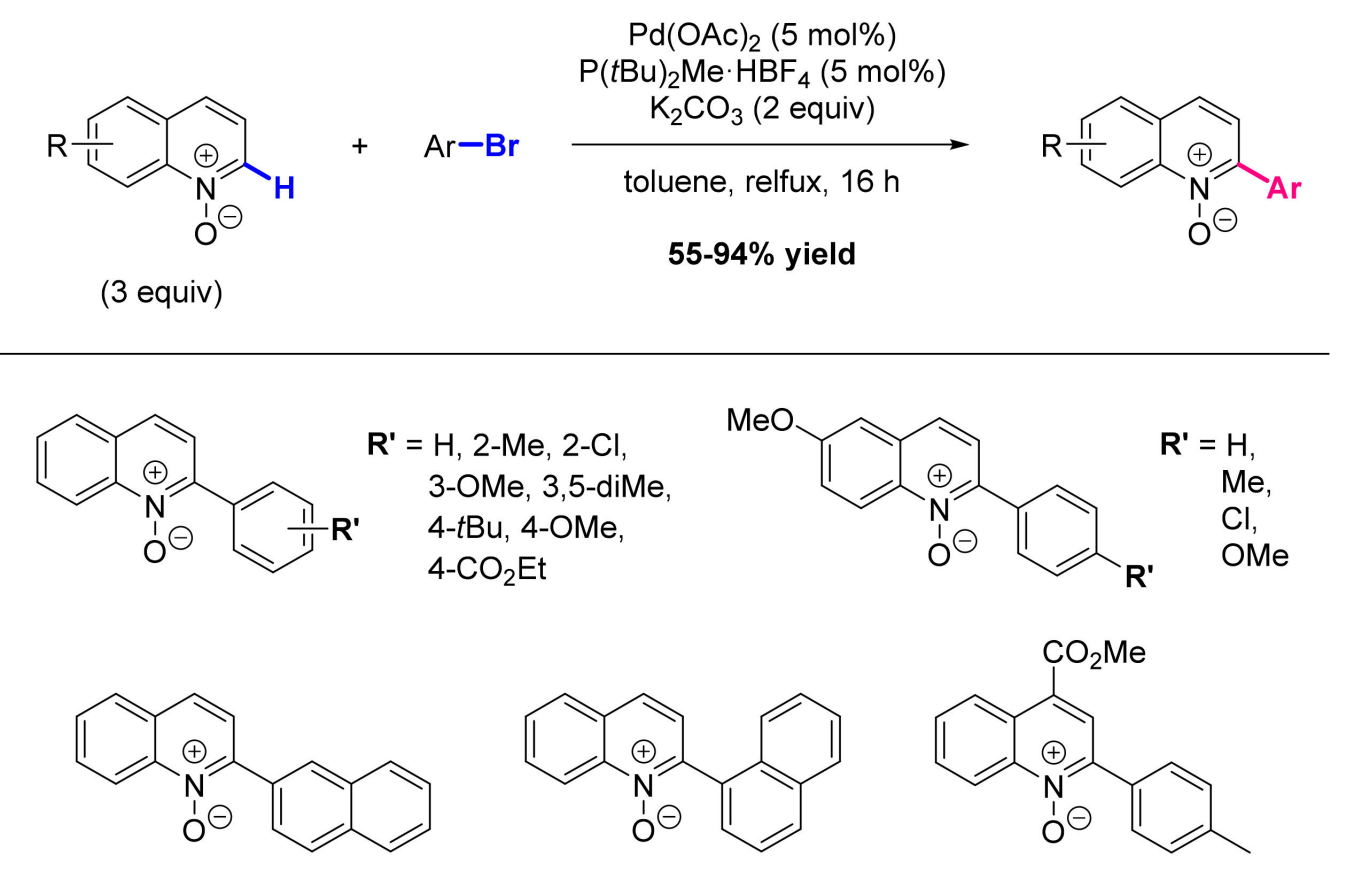
Pd-catalyzed C2 arylation of quinoline N-oxides with aryl bromides.

In 2009, the group of Fagnou described another palladium-catalyzed arylation method, using aryl-bromides as coupling partners and a broad range of azines and azoles as substrates [109]. Starting from a catalytic system composed of Pd(OAc)_2_ (5 mol%) and K_2_CO_3_ (2 equiv), various ligands and solvents were screened using different amounts of quinoline *N*-oxide, which resulted in the selection of di-*t*-butyl-methylphosphonium tetrafluoroborate (5 mol%) in refluxing toluene as the best option to arylate 3 equiv of substrate in nearly quantitative yield. When the generality of this reaction was evaluated, it proved to be compatible with a range of functional groups and substitution patterns on the aryl bromide, with both EDGs and EWGs well tolerated on the quinoline *N*-oxide, leading to a series of fifteen C2-arylated QNOs in good 55–94 % yields (Scheme 6). Noteworthy, the subsequent *N*-oxide deoxygenation could efficiently be achieved in the presence of Pd/C (10 mol%) and ammonium formate (10 equiv) in methanol (0.1 M) at room temperature, leading to the QN derivatives in high yields.

The mechanism proposed by the authors is a Pd(0)/Pd(II) process that corresponds to the *path b* described above in the Scheme 3, involving a standard OA/RE sequence. After detailed mechanistic studies carried out with pyridine *N*-oxide, it was demonstrated that C-H activation of the quinoline follows an inner-sphere CMD pathway, for which the acetate from the Pd(OAc)_2_ precatalyst is acting as the deprotonating agent in the step **II** [110].

**Scheme 7:**
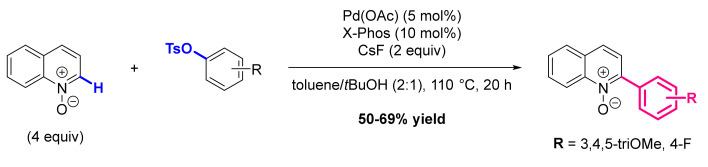
Pd-catalyzed C2 arylation of quinoline *N*-oxide with aryl tosylates.

In 2011, Ackermann et al. reported another method for the direct Pd-catalyzed arylation of electron-deficient (hetero)arenes using aryl tosylates as aryl donors [111]. The model arylation of pyridine *N*-oxide (4 equiv) with 3,5-dimethylphenyl tosylate (1 equiv) catalyzed by Pd(OAc)_2_ (5 mol%) was optimized through the screening of various ligands, organic and inorganic bases, solvent systems and additives, which highlighted that superior results are obtained with X-Phos (10 mol%) and CsF (2 equiv) in a *t-*BuOH/toluene 2:1 solvent mixture. In these conditions, the selective C2-arylation of quinoline *N*-oxide with two differently substituted aryl tosylates was achieved in good yields (Scheme 7).

**Scheme 8:**
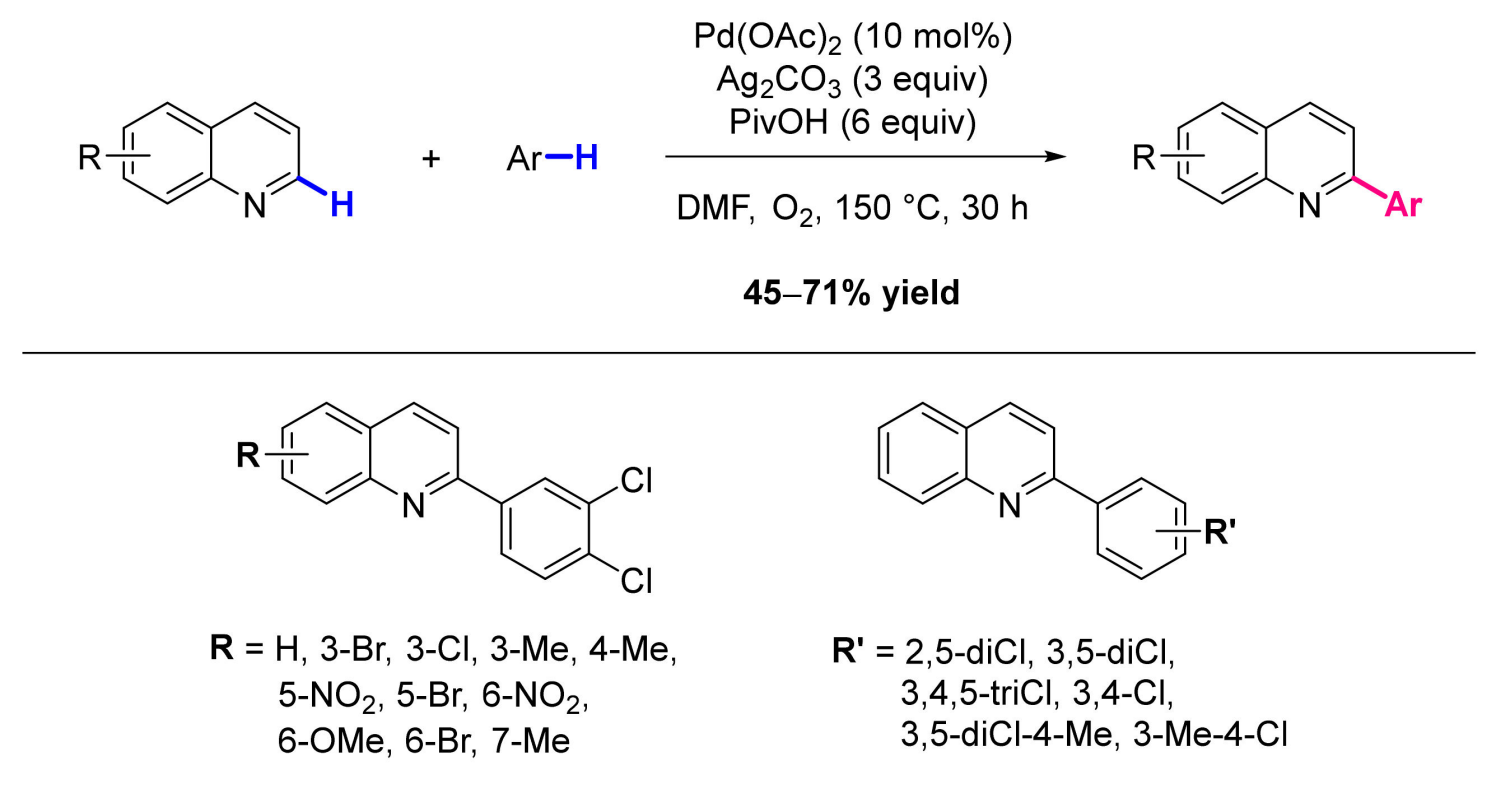
Pd-catalyzed C2 arylation of quinolines with unactivated arenes.

The last example of Pd-mediated arylation was reported in 2013 by the group of Huang who developed an efficient Pd-catalyzed oxidative CH/CH coupling of quinolines with unactivated arenes [112]. As described earlier for the analogous method of Chang [108], the reaction requires a large excess of benzene derivative and with 10 mol% of Pd(OAc)_2_, after a rapid screening of few oxidants, organic acids and solvents, the addition of Ag_2_CO_3_ (3 equiv) and PivOH (6 equiv) in DMF appeared to be the best conditions. Moreover, even if the reaction does not require external oxidant, the addition of O_2_ proved to enhance significantly the yield. Examination of the scope with various QNs and chlorobenzene derivatives resulted in the synthesis of seventeen C2-arylated QNs in moderate to good yields ranging from 45 to 71% (Scheme 8). Regarding the substituents on the QN, electronic properties seem to be more influential than steric effects and moreover, better yields were obtained with EWGs than EDGs.

The mechanism proposed by the authors involves a double C-H activation process that matches with the *path a* described above in the Scheme 3, for which the pivalate ligand play the role of the base for the deprotonation step **II**, and the silver salt is necessary to reoxidize the Pd (0) into Pd(II) after the RE step **IV**.

**Scheme 9:**
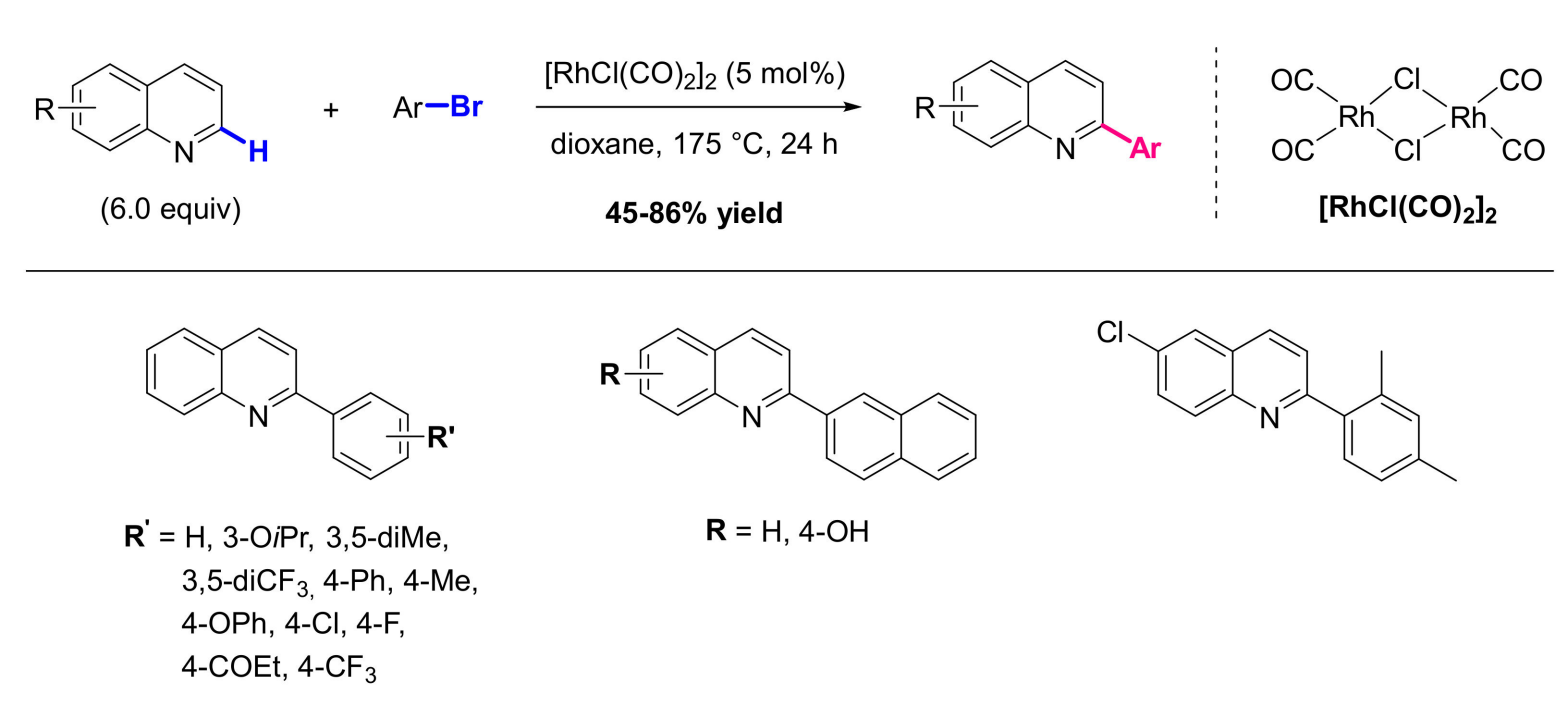
Rh-catalyzed C2 arylation of quinolines with aryl bromides.

The C2-arylation of quinoline can also be performed via a Rh-based processes, as exemplified by the work of Bergman and Ellman published in 2008 and 2010, relying on the commercially available and air-stable catalyst [RhCl(CO)_2_]_2_. In the first report, the arylation is performed with aryl bromides [113]. Starting from the conditions they had previously developed for the alkylation of QNs [114], the cross coupling of 2-methylpyridine (3 equiv) and 3,5-dimethylphenyl bromide (1 equiv) with 0.05 equiv of catalyst was studied by adding some additives or varying the concentration and the temperature. This optimization revealed that absolute concentration is the most important parameter, with values of 0.8 M for the pyridines and 0.3 M for the QNs. The generality of this protocol was investigated by coupling a few QNs with various aryl bromides, which resulted in the synthesis of several C2-arylated QNs in a yield range of 45 to 86% (Scheme 9). Both electron-rich and electron-poor substituents on the aryl donor proved to be tolerated with equal efficiency, whereas cross-coupling with 2-bromotoluene failed to yield the expected product.

**Scheme 10:**
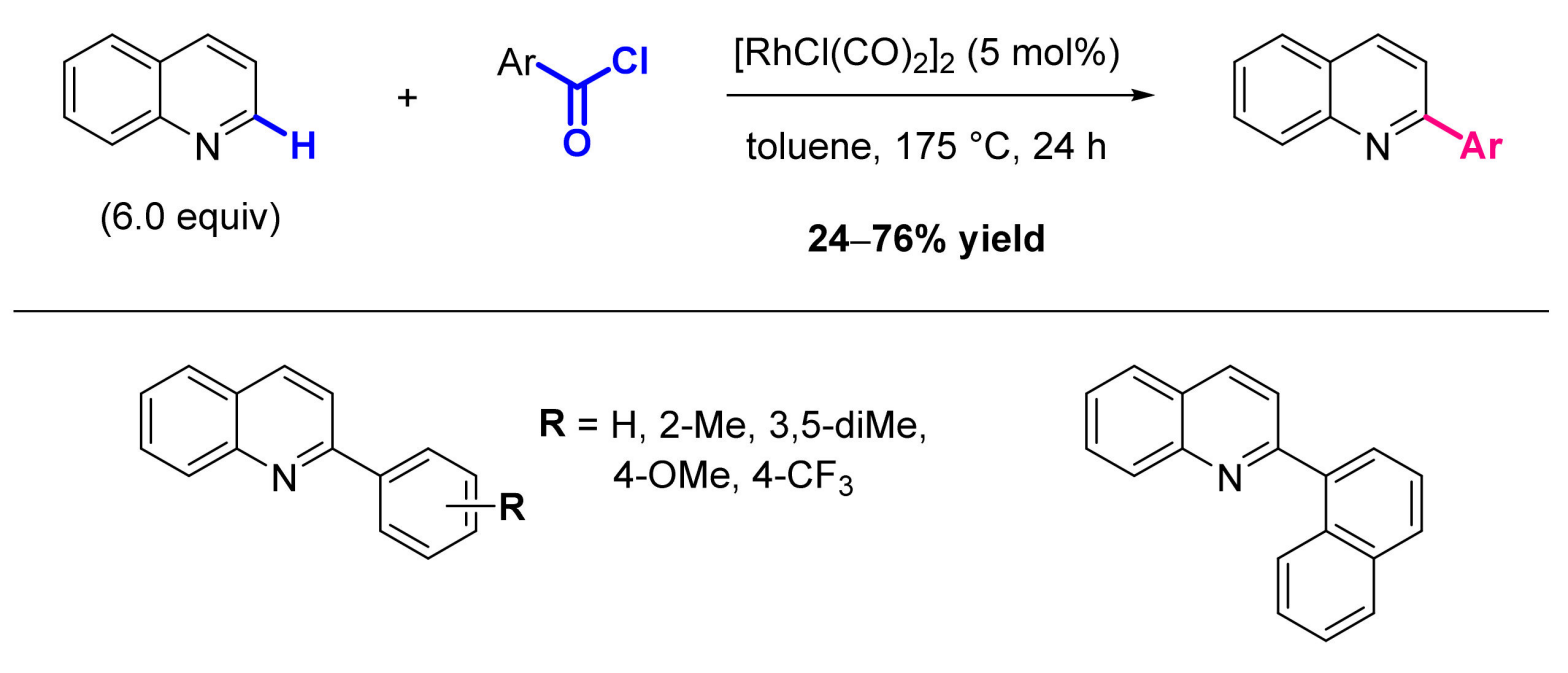
Rh-catalyzed C2 arylation of quinoline with aroyl chlorides.

In the second report, the scope of the previous method was expanded with four new examples, and more importantly, conditions were adapted to the use of aroyl chlorides as coupling partners in order to achieve arylation via a decarbonylation pathway [115]. Additives, concentration and temperature were once again studied as influential parameters for the [RhCl(CO)_2_]_2_-catalyzed arylation of quinoline (6 equiv) with benzoyl chloride (1 equiv) and it was found that switching the solvent from dioxane to toluene had a positive impact whereas the addition of a base or the variation of concentration proved to be deleterious for the reaction. In these optimized conditions, six different aroyl chlorides were used as coupling partners and led to the synthesis of the corresponding C2-arylated QNs in modest to good yields (Scheme 10). Noteworthy, electron rich or sterically hindered coupling partners gave the expected product in yields ranging from 52 to 76% whereas electron-poor 4-trifluoromethyl benzoyl chloride revealed turned out to be less efficient with 24% yield.

The mechanism proposed by the authors is similar to the *path a* described above in the Scheme 3, involving a standard OA/RE sequence. In the particular case of aroyl chlorides, the OA step **III** is followed by decarbonylation to furnish the key intermediate **D**.

**Scheme 11:**
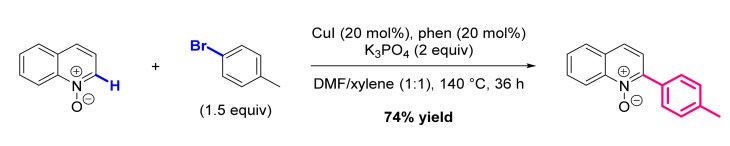
Cu-catalyzed C2 arylation of quinoline *N*-oxide with 4-bromotoluene.

At the same period, the group of You disclosed the sole example of Cu-catalyzed arylation of quinoline *N-*oxide with 4-bromotoluene [116]. The conditions were optimized for the coupling of caffeine (1 equiv) with *p*-bromotoluene (1.5 equiv) using CuI (20 mol%) as catalyst through the screening of various ligands, bases and solvents, which revealed that 1,10-phenanthroline (phen, 20 mol%) with K_3_PO_4_ (2 equiv) in a DMF/xylene 1:1 mixture is the best combination. Among the various heteroarenes that were tested to expand the scope of this reaction, QNO turned out to be a good substrate and afforded the corresponding C2-arylated product in 74% yield (Scheme 11).

**Scheme 12:**
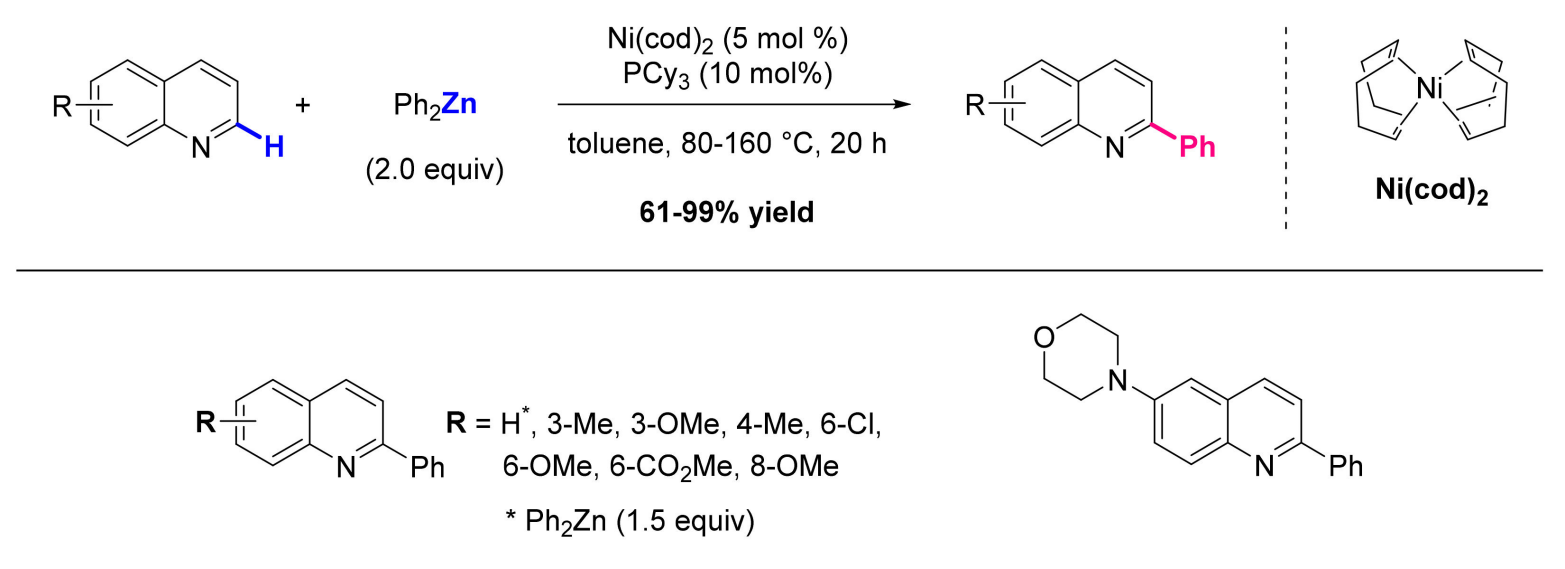
Ni-catalyzed C2 phenylation of quinolines with diphenylzinc reagent.

Finally, Tobisu and Chatani described in 2012 the Ni-catalyzed arylation of electron-deficient heteroarenes, including quinoline, with organozinc reagents [117]. After a rapid screening of different metal-based catalysts mixed with PCy_3_ (10 mol%), Ni(cod)_2_ (5.2 mol%) was found to be the most efficient to achieve the arylation of QN (1 equiv) with diphenyl zinc (1.5 equiv) in toluene. QNs substituted by diverse functional groups, including chloro, methoxy, amino or even ester substituents, proved to be compatible with the transformation, leading to a short series of C2-arylated products in good to high yields ranging from 61 to 99% (Scheme 12). Noteworthy, electron-deficient substrates were generally arylated more rapidly than electron-rich ones, and these conditions are tolerant to steric hindrance as evidenced by the high yields obtained for substrates substituted at C3 or C8 positions.

**Scheme 13:**
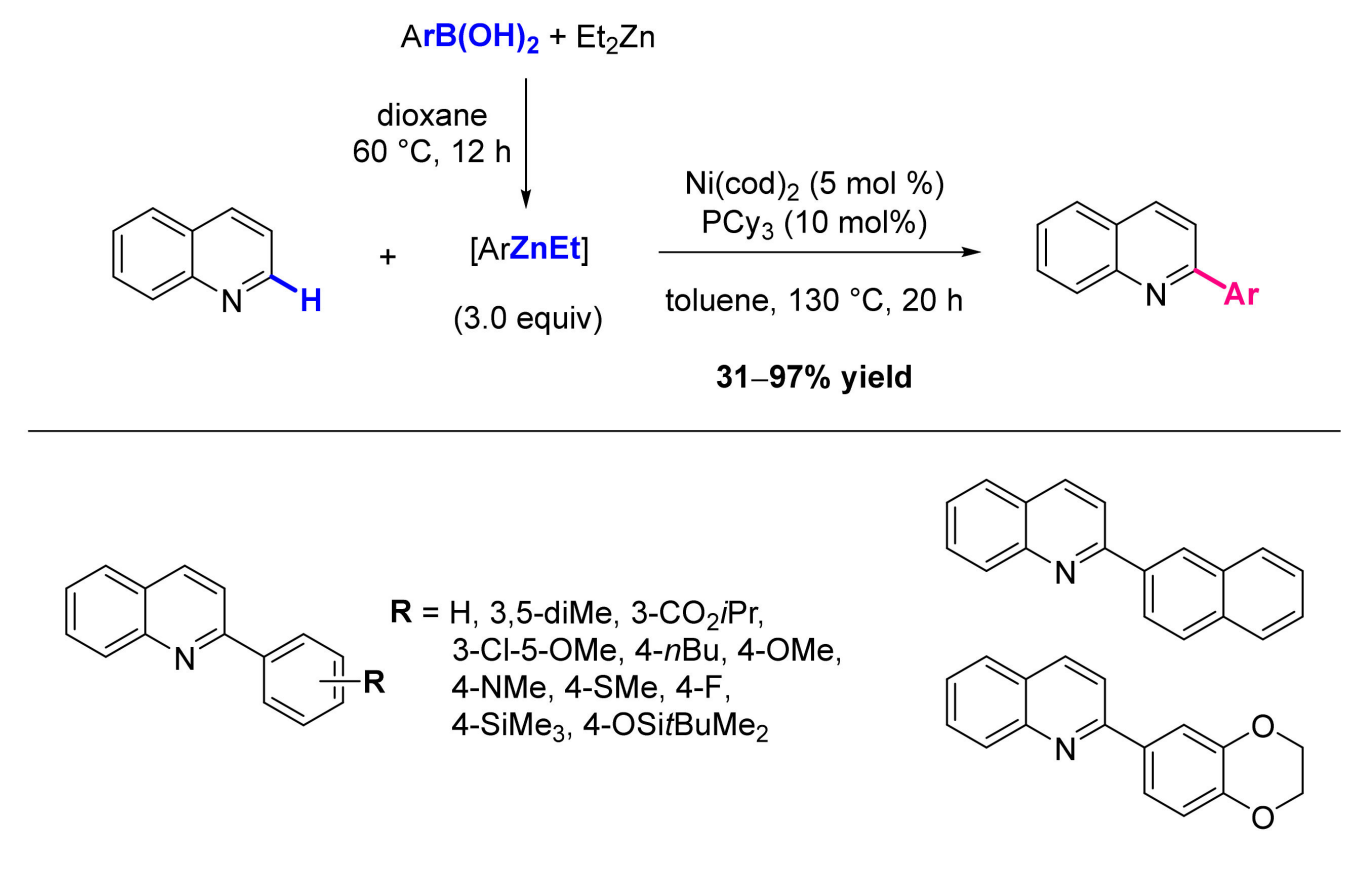
Ni-catalyzed C2 arylation of quinoline with arylzinc reagents.

In order to study the scope of the reaction, various organozinc reagents were prepared *in situ* from aryl boronic acids, avoiding thus the formation of metal salts that may have a detrimental effect on the nickel-catalyzed process. Thirteen different organozinc reagents were tested as coupling partners and led to the synthesis of the corresponding C2-arylated quinolines in moderate to high yields (Scheme 13). As anticipated, the reaction proved to be more efficient with an electron-rich than with an electron-deficient aryl donor.

The mechanism proposed by the authors does not correspond to any of the pathways described above in the Scheme 3 and the reader is invited to consult the original article for a detailed description if desired.

### 2.2. Formation of C-HetAr Bonds

While the C2-arylation has been reported with different metals, introduction of heteroaromatics in position 2 was exclusively described with Pd-based catalytic systems. The vast majority of these methods rely on a double C-H activation process, or dehydrogenative cross-coupling (DCC), that requires the addition of silver or copper salts in order to regenerate the catalytic species. However, none of these protocols was studied with a full panel of quinolines, or the corresponding *N*-oxides. In each case, only a few examples of QN were reported as part of a bigger scope of azines.

**Scheme 14:**
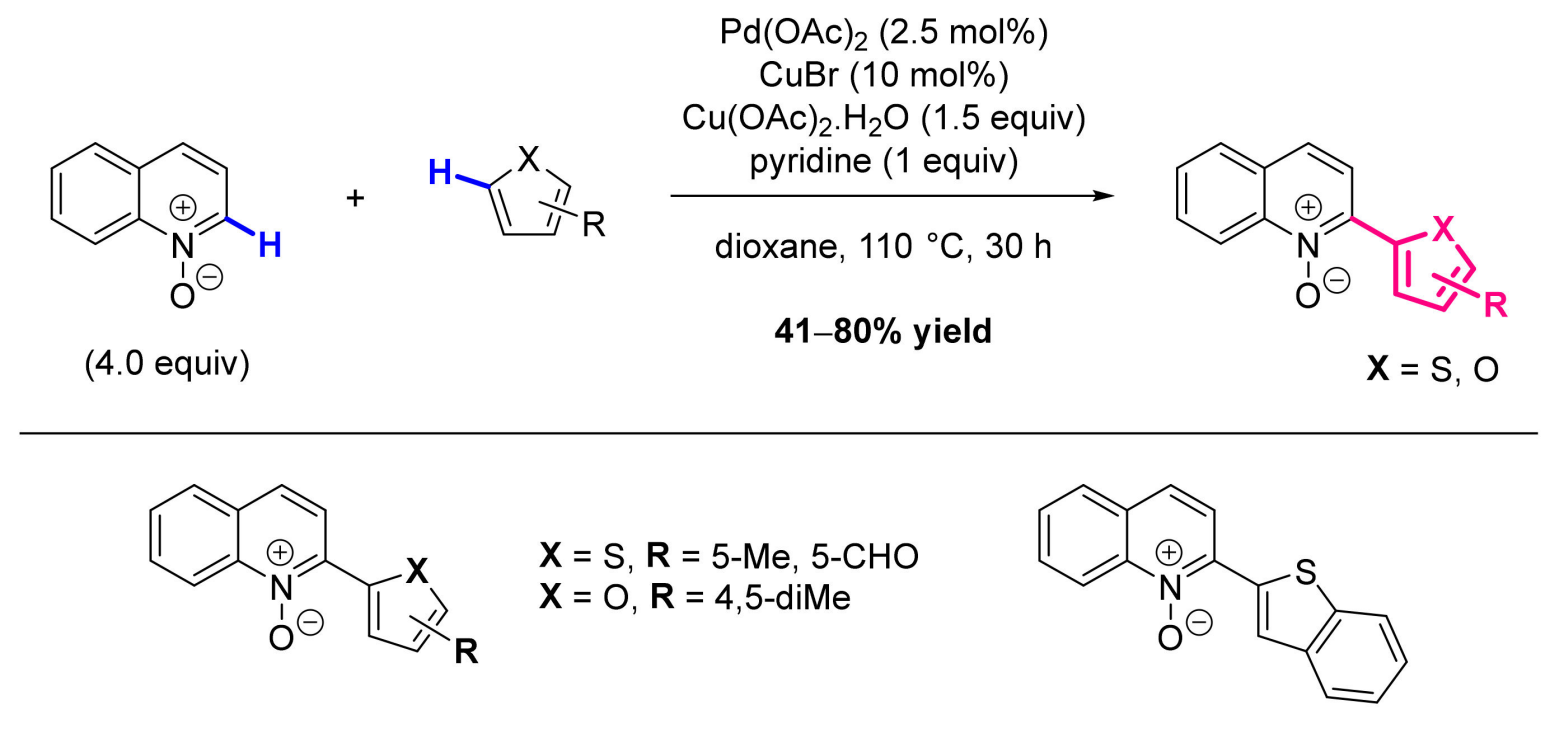
Pd-catalyzed C2 oxidative cross-coupling of quinoline *N-*oxide with heteroarenes.

The first report was published in 2013 by the group of You, who developed the Pd-catalyzed oxidative C-H/C-H cross-coupling of xanthines and other *N*-containing heteroarenes with thiophens or furans [118]. The optimization was conducted on the coupling of caffeine (1 equiv) with 2-formylthiophene (3 equiv) catalyzed by Pd(OAc)_2_ (2.5 mol%). The screening of several parameters such as oxidant, additive and solvent, resulted in the choice of Cu(OAc)_2_⋅H_2_O (1.5 equiv), pyridine (1 equiv) and 1,4-dioxane. However, when the scope of the reaction was investigated, it was found that 4 equiv of quinoline *N*-oxide and addition of CuBr (10 mol%) were necessary to retain high efficiency. In these adapted conditions, the reaction of quinoline *N*-oxide with a panel of five coupling partners afforded the desired unsymmetrical biheteroaryl products in a yield range of 41 to 80% (Scheme 14). Interestingly, the homocoupling, if detectable, is limited to ∼10% and the synthesis of 2-(5-methylthiophen-2-yl)-quinoline *N*-oxide was performed without problems on an ∼2 g scale.

According to the DFT calculation based on the coupling of *N*-methylimidazole and thiophene, the mechanism proposed by the authors is analogous to the *path b* described above in the Scheme 3, relying on a double C-H activation sequence for which Cu-mediated reoxidation of Pd(0) into Pd(II) is necessary to close the catalytic cycle after the RE step **IV**.

**Scheme 15:**
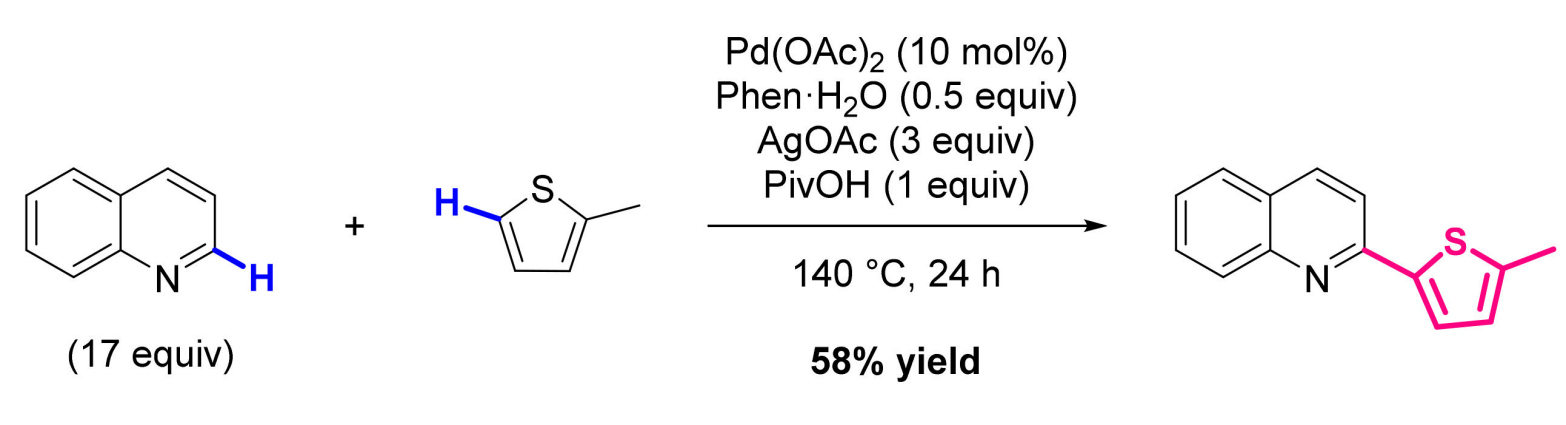
Pd-catalyzed C2 oxidative cross-coupling of quinoline *N-*oxide with 2-methylthiophene.

In 2013, the same group devised another catalytic system in order to achieve the step-economic arylation of non-oxidized substrates [119]. The model reaction of pyridine (25 equiv) with 2-methylthiophene (1 equiv) catalyzed by Pd(OAc)_2_ was optimized by screening various oxidants, ligands and additives, which showed that the addition of AgOAc (3 equiv), Phen·H_2_O (0.5 equiv) and PivOH (1 equiv) is necessary to reach the best conversion. In these conditions, the use of quinoline (17 equiv) as substrate led to the desired product in 58% yield with excellent regioselectivity (Scheme 15).

The mechanism proposed by the authors involves a double C-H activation process that matches with the *path a* described above in the Scheme 3, which ends with the Ag-mediated reoxidation of Pd (0) into Pd(II), after the RE step **IV**, to regenerate the catalytic species. Experimental investigation carried out with TEMPO ruled out a radical mechanism and measurement of KIE showed that step **II** is the rate-limiting step and follows a carboxylate-assisted CMD pathway.

**Scheme 16:**
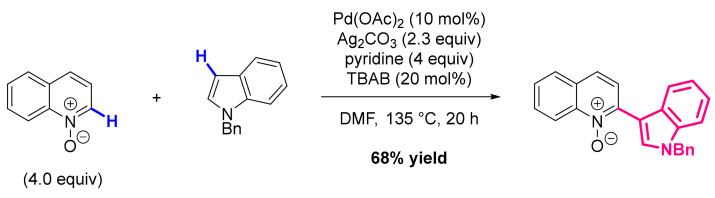
Pd-catalyzed C2 oxidative cross-coupling of quinoline *N-*oxide with indole.

Over the same period, Zhang and Li disclosed another Pd-based catalytic system to achieve the cross-coupling of pyridine *N*-oxides and indoles [120]. This reaction represents an interesting challenge since indoles are prone to decomposition in oxidative conditions [121]. Optimization of the reaction conditions was performed with pyridine *N*-oxide (4 equiv), *N*-benzylindole (1 equiv) and Pd(OAc)_2_ (10 mol%), by screening diverse oxidants, bases and additives. It demonstrated that Ag_2_CO_3_ (2.3 equiv), pyridine (4 equiv) and tetrabutylammonium bromide (20 mol%) were the most effective. In these conditions, the C2 heteroarylation of quinoline *N*-oxide was achieved in 68% yield, with the C-H functionalization selectively occurring at position 3 of the indole (Scheme 16).

**Scheme 17:**
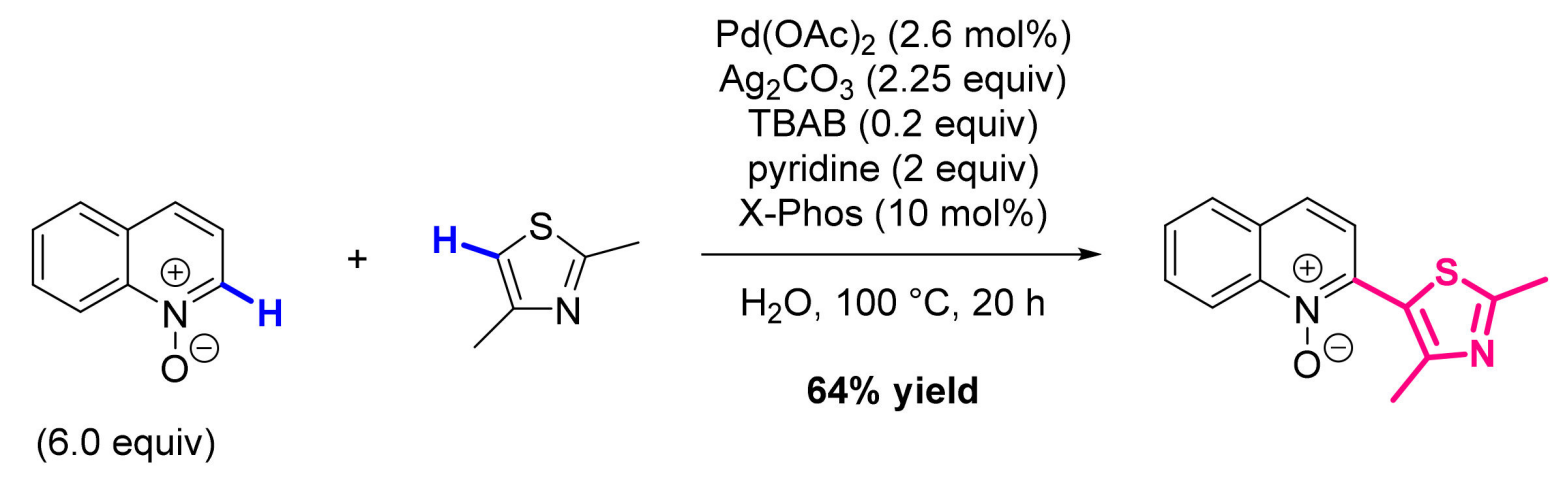
Pd-catalyzed C2 oxidative cross-coupling of quinoline *N-*oxide with thiazole.

The last example of Pd-catalyzed DCC was reported in 2014 by Smith et al. who, inspired by the work of Zhang and Li, expanded the scope to the coupling of pyridine *N*-oxide and thiazole “on water” [122]. With pyridine-*N*-oxide (6 equiv) and 2,3-dimethylthiazole (1 equiv) as model substrates, the Zhang and Li’s conditions were firstly tested against various solvents: water at 100 °C proved to give better yields than DMF at 135 °C. Further investigations regarding the amount of substrate, catalyst, base and oxidant, as well as the addition of various ligands, allowed to identify Pd(OAc)_2_ (2.6 mol%), pyridine (2 equiv) and X-Phos (10 mol%) as optimized conditions. When the scope was evaluated, quinoline *N*-oxide was found to be a good substrate, leading to the C2-heteroarylated product in a good 64% yield (Scheme 17).

The plausible reaction pathway proposed by the authors corresponds to the *path b* described above in the Scheme 3, relying on a double C-H activation process with subsequent Ag-promoted reoxidation of Pd(0) into Pd(II) after the RE step **IV** to close the catalytic cycle.

**Scheme 18:**
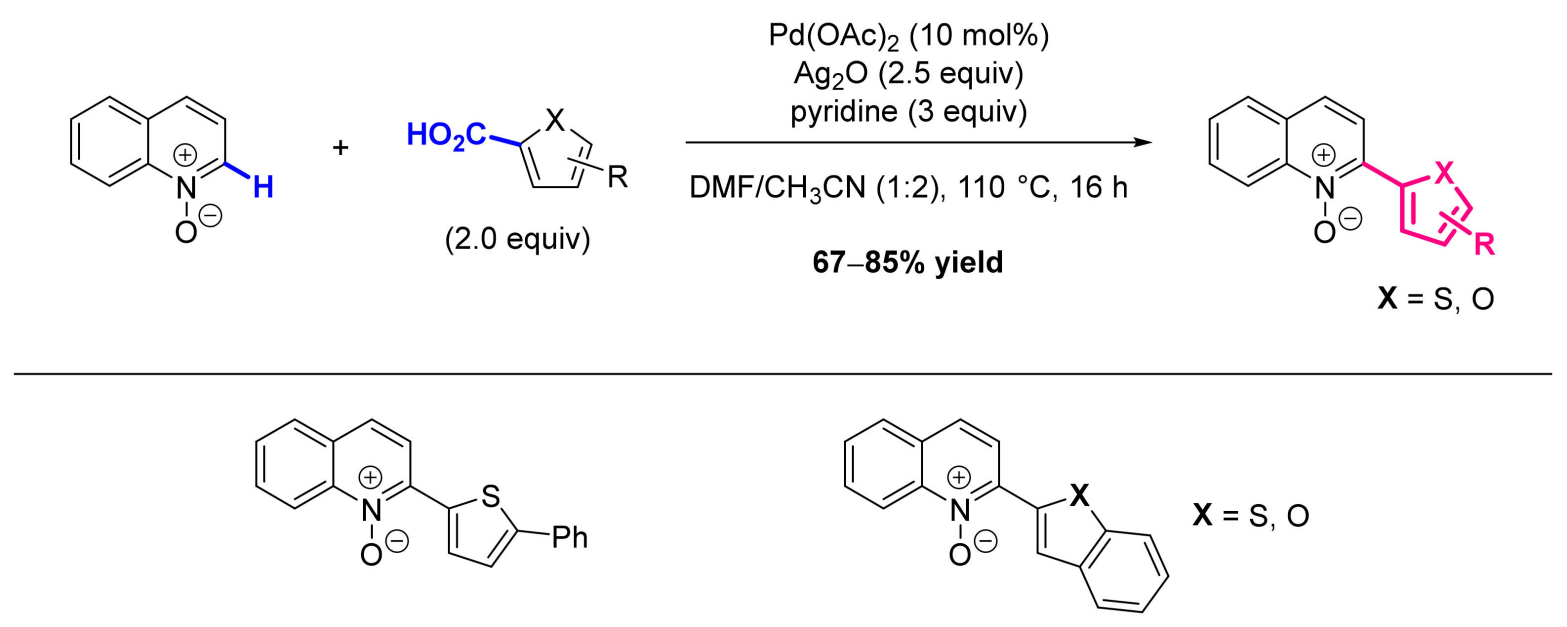
Pd-catalyzed C2 decarboxylative-coupling of quinoline *N*-oxide with carboxylic acids.

The same year, complementing these DCC methods, a Pd-catalyzed decarboxylative coupling was developed by Muthusubramanian’s team to achieve the C2-arylation of pyridine *N*-oxides with heteroaryl carboxylic acids [123]. The feasibility of this coupling was assessed using 4-methylpyridine *N*-oxide (1 equiv) and 5-phenylthiophene-2-carboxylic acid (1.5 equiv) as model substrates and, after the screening of various catalysts, bases, ligands/additives and solvents, the combination of Pd(OAc)_2_ (10 mol%), Ag_2_O (2.5 equiv) and pyridine (3 equiv) in a mixture of DMF/CH_3_CN (1:2) proved to be optimal. These conditions allowed the coupling of quinoline *N*-oxide with benzo[b]thiophene- and benzofuran-2-carboxylic acids, affording the parent C2-heteroarylated QNO derivatives in good yields ranging from 67 to 85% (Scheme 18).

The mechanism proposed by the authors is consistent with the *path b* described above in the Scheme 3, starting with the Ag-promoted decarboxylation of the heteroaryl carboxylic acid, followed by transmetalation to furnish the Pd-intermediate **C′**. From there, C2-H activation of the QNO and subsequent RE (step **IV**) yield the C2-arylated product and Pd(0) which is finally reoxidized by Ag(I) species.

### 2.3. Formation of C-C=C Bonds

The direct C-H alkenylation of quinoline *N*-oxide has been reviewed by the group of Kouznetsov in 2020 [100]. However, several of these methods do not rely on a C-H activation mechanism and fall therefore outside the scope of our review. If one stays focused on C-H activation processes, almost all the alkenylation processes reported to date are using Pd-based catalysis.

**Scheme 19:**
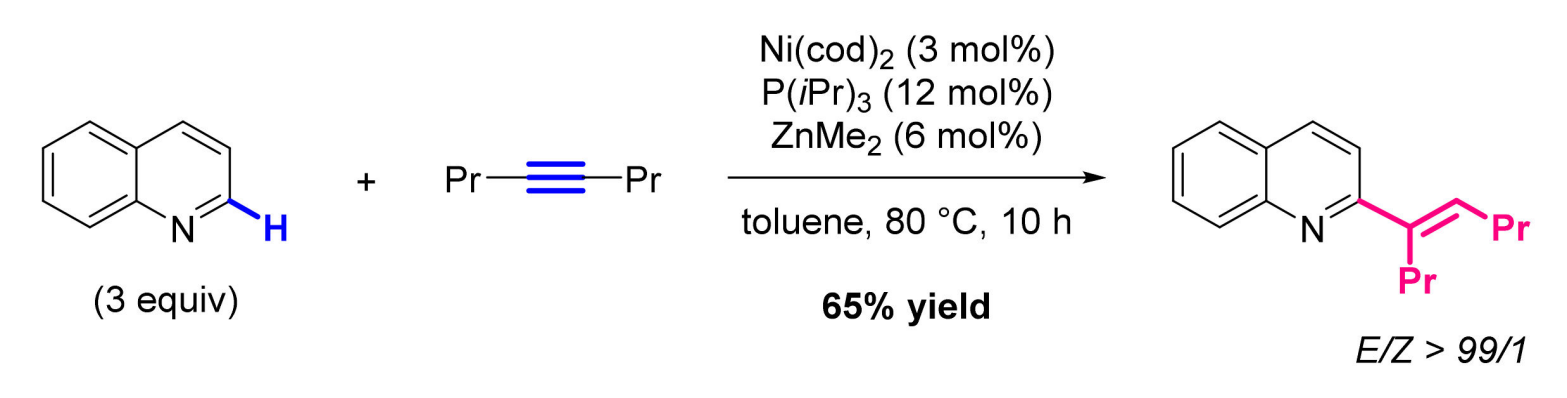
Ni-catalyzed C2 alkenylation of quinoline with 4-octyne.

The first strategy was reported in 2008 by Nakao and Hiyama who developed a nickel and Lewis acid catalytic system for the alkenylation of pyridines with alkynes [124]. In continuation of their previous work on pyridine *N*-oxides [125], the authors envisaged to generate *in situ* an activated pyridine through the coordination of the nitrogen to a LA catalyst. The impact of various LA was examined towards a model reaction involving a mixture of pyridine (3 equiv), 4-octyne (1 equiv), Ni(cod)_2_ (3 mol%) and P(i-Pr)_3_ (12 mol%), and ZnMe_2_ (6 mol%) appeared to be the best candidate. When applied to the alkenylation of quinoline, these optimized conditions afforded the desired C2-alkenylated product in 65% yield with a high regio- and stereoselectivity (Scheme 19).

The mechanism proposed by the authors is similar to the *path a* described above in the Scheme 3, starting with the ZnMe_2_-promoted metalation-deprotonation of the heterocycle (step **II**), followed by hydronickelation across the alkyne (step **III**) and subsequent reductive elimination (step **IV**). However, another mechanism involving a metallacycle cannot be excluded [126].

**Scheme 20:**
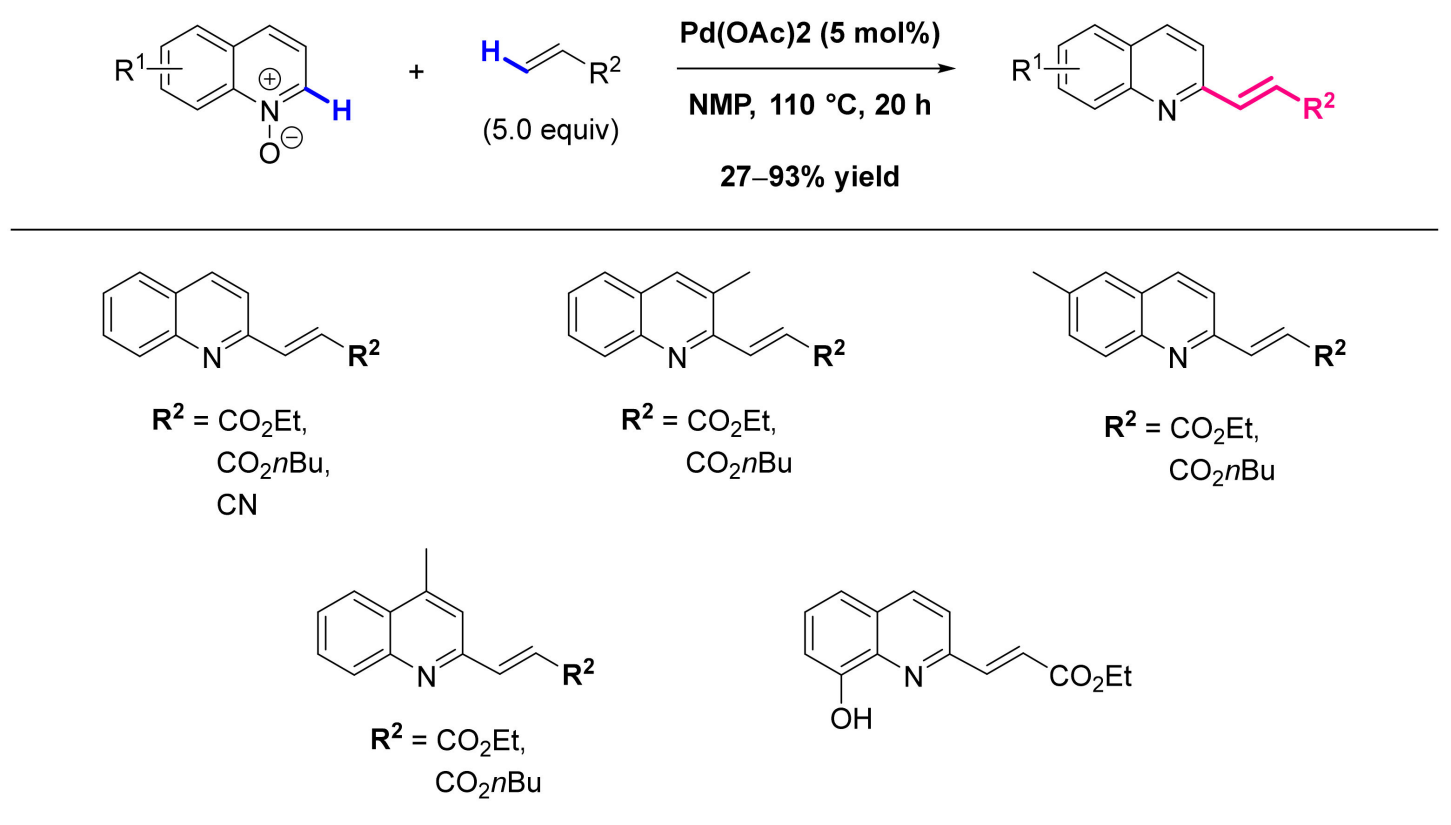
Pd-catalyzed C2 alkenylation of quinoline *N*-oxides with alkenes.

In 2009, Cui and Wu proposed the first Pd-catalyzed alkenylation method of quinoline *N*-oxides with acrylates under external-oxidant-free conditions, assuming that the *N*-oxide moiety might serve as both a directing group and an oxidant [127]. A rapid optimization of the reaction conditions towards the coupling of quinoline *N*-oxide (1 equiv) and ethyl acrylate (5 equiv) with Pd(OAc)_2_ (5 mol%) led to the identification of NMP as the best solvent. The scope of this reaction was evaluated with several QNOs and acrylates, affording the synthesis of ten diversely substituted C2-alkenylated quinolines in 27–97% yield range (Scheme 20). The best results were obtained for quinoline *N-*oxides substituted by electron-donating methyl groups at the C3, C4, and C6 positions whereas no reaction was observed with the 4-NO_2_ derivative.

The mechanism proposed by the authors is consistent with the *path a* described above in the Scheme 3, involving a *syn* insertion/β-hydride elimination sequence that releases a QNO intermediate and a Pd-hydride, followed by a final redox step to furnish the alkenyl-QN product and regenerate the catalyst.

**Scheme 21:**
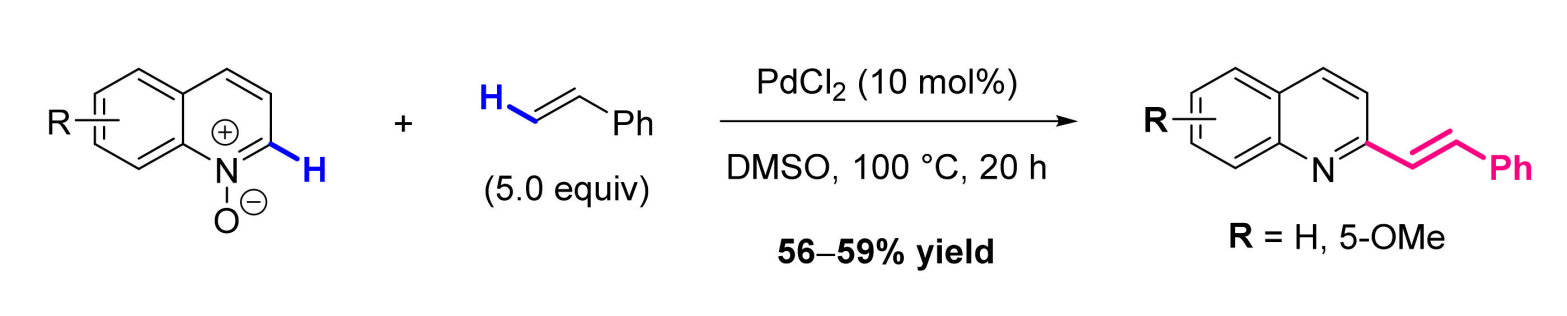
Pd-catalyzed C2 alkenylation of quinoline *N*-oxides with styrene.

In 2016, Han and Liu described an analogous external-oxidant-free protocol for the coupling of quinoline *N*-oxide with olefins [128]. Using isoquinoline *N*-oxide (1 equiv) and styrene (5 equiv) as model substrates, the screening of various Pd-based catalysts, additives and solvents showed that the optimal conditions require PdCl_2_ (10 mol%) and DMSO. When these conditions were applied to QNO and its 5-OMe derivative, the expected C2-alkenylated products were obtained in moderate 54–59 % yields (Scheme 21).

The mechanism proposed by the authors corresponds to the *path a* described above in the Scheme 3, with the same sequence of steps as that described by Cui and Wu for the previous example.

**Scheme 22:**
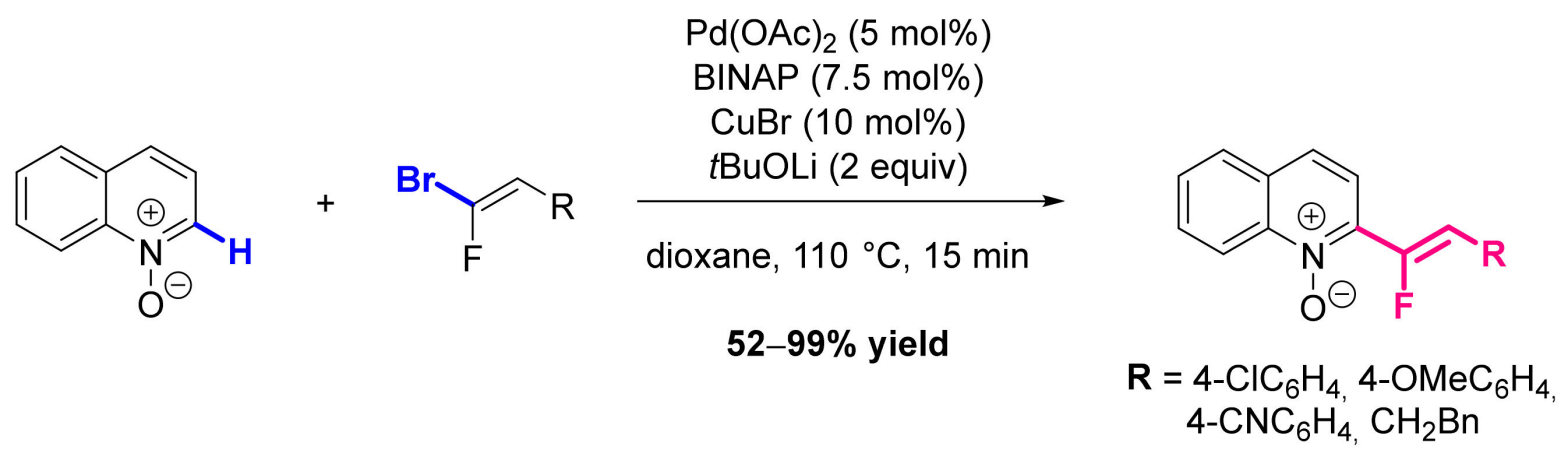
Pd-catalyzed C2 alkenylation of quinoline *N*-oxide with bromoalkenes.

One year later, the group of Couve-Bonnaire developed a bicatalytic Pd/Cu-based system that allows the direct C-H fluoroalkenylation of pyridine *N*-oxides and related derivatives with *gem*-bromofluoroalkenes as electrophiles [114]. Unlike the previous methods, in this case the *N*-oxide moiety is not reduced during the reaction. The first optimization aimed at the development of an efficient Cu-based catalytic system but the desired alkenylation reaction turned out to be hampered by the significant degradation of both the coupling partner and the product. Starting with a model system composed of 2-methylpyridine-*N*-oxide (1 equiv), *gem*-bromofluoroalkene (1.2 equiv), CuBr (10 mol%) and *t*BuOLi (2 equiv), various Pd-based catalysts and ligands were tested and the combination of Pd(OAc)_2_ (5 mol%) and BINAP (7.5 mol%) appeared to enhance remarkably the yields. In these fully optimized conditions, quinoline *N*-oxide reacted smoothly with four different alkenes to furnish the desired products in good yields varying from 52 to 99% (Scheme 22).

According to the authors, the mechanism of this bicatalytic Pd/Cu process is similar to the one which was previously postulated by Bellina and Rossi [129], in which the copper is necessary to form the heteroarylcopper species that is then involved in the transmetalation step with the palladium.

**Scheme 23:**
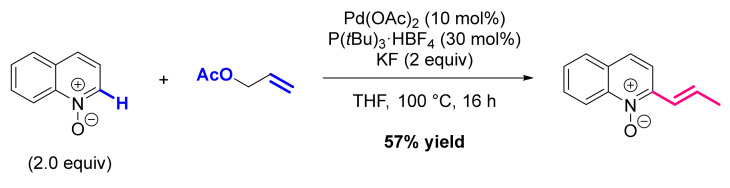
Pd-catalyzed C2 alkenylation of quinoline *N*-oxide with allyl acetate.

The last example of alkenylation was disclosed in 2018 by the team of Poli, who developed the Pd-catalyzed C-H alkenylation and allylation of azine *N*-oxides using allyl acetates as coupling partners [130]. Various ligands, bases and solvents were screened towards the coupling of pyridine *N*-oxide (2 equiv) and allyl acetate (1 equiv) with Pd(OAc)_2_ (10 mol%), and optimized conditions proved to be the use of P(*t*-Bu)_3_·HBF_4_ (30 mol%) and KF (2.0 equiv) in THF. When the generality of the reaction was investigated, quinoline *N*-oxide reacted satisfactorily to give the corresponding C2-alkenylated product in 57% yield (Scheme 23).

After extensive deuterium labeling experiments and DFT calculations, the authors proposed a mechanism that is similar to the *path b* described above in the Scheme 3, starting with the OA of the allyl acetate to form an allylpalladium complex **C′**, followed by the C2-H activation to furnish the key intermediate **D**. From there, the RE step **IV** yield an intermediate C2-allyl QNO that is finally isomerized into the more conjugated C2-crotyl product.

### 2.4. Formation of C-Alkyl Bonds

Introduction of alkyl chains in position 2 of quinolines was successively developed with Rh-, Pd- and more recently Cu-catalytic systems. Most of these methods are using quinoline *N*-oxides as starting material.

**Scheme 24:**
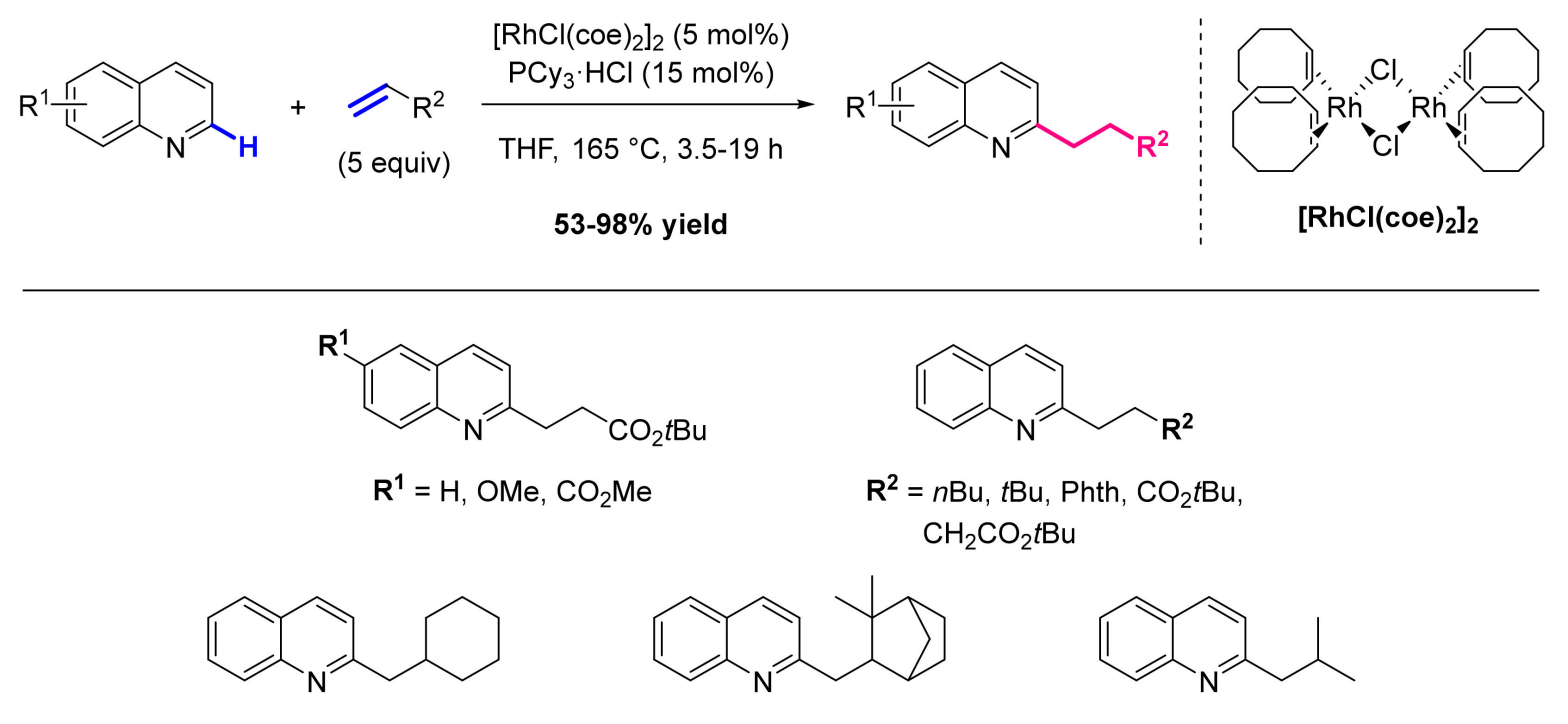
Rh-catalyzed C2 alkylation of quinolines with alkenes.

In 2007, Bergman and Ellman reported the Rh-catalyzed alkylation of quinolines and pyridines with alkene [96]. A Rh/PCy_3_ system was used to perform both the activation and the alkylation of 2-methyl pyridine. The phosphine, additive and concentration of the reaction were rapidly screened towards the coupling of 2-methylpyridine (1 equiv) and 3,3-dimethylbutene (5 equiv). The optimal conditions involved RhCl(coe_2_)_2_ (0.05 equiv) and PCy_3_·HCl (0.15 equiv) as an acid additive at a 0.8 M concentration in the heterocycle, in THF. The investigation of heterocycle scope showed that quinoline, ether- and ester 6-substituted QNs are alkylated in excellent yields. The scope of the alkene was next examined: mono- or disubstituted alkylated olefins, cyclohexene and camphene revealed to be suitable partners for this reaction and both esters and phthalimides were also found to be compatible with the reaction conditions (Scheme 24).

**Scheme 25:**
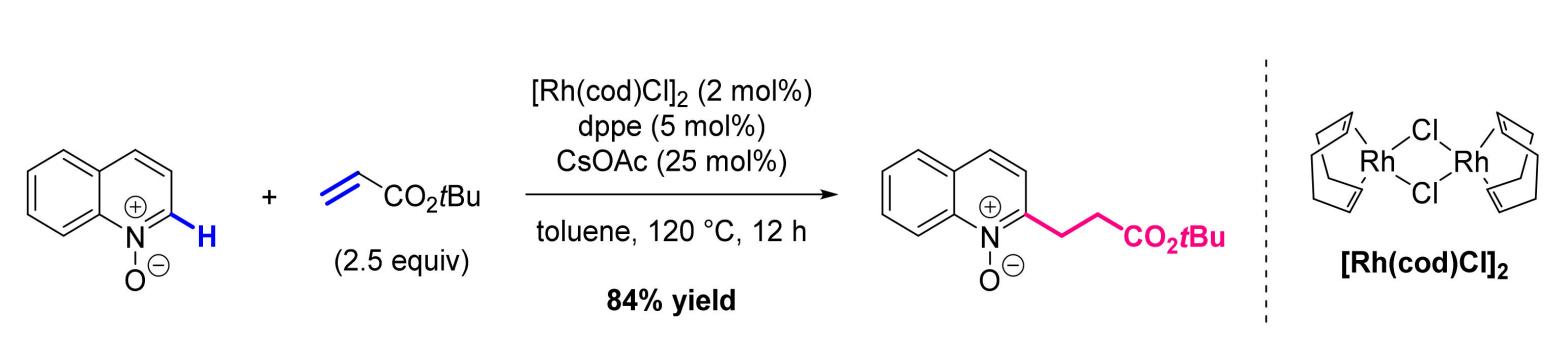
Rh-catalyzed C2 alkylation of quinoline *N*-oxide with *tert*-butyl acrylate.

In 2012, Cho and Chang reported another rhodium catalyst system that enabled the highly selective C2 activation of heteroarenes for the hydroarylation of both alkenes and alkynes [131]. Various combinations of rhodium species, ligands and bases were screened for the alkylation of the pyridine-*N*-oxide (2.5 equiv) with *tert*-butyl acrylate (1 equiv) and the best conditions of the reaction revealed to be [Rh(cod)Cl]_2_ (2 mol%), 1,2-bis-(diphenylphosphino)ethane (5 mol%) as a bidentate phosphine ligand and CsOAc (25 mol%) in toluene (0.5 mL), leading to the C2-alkylated product in 90% isolated yield, with 3% of the 2,6-dialkylated compound as a side product. These conditions revealed to be highly efficient on quinoline *N*-oxide, leading to the corresponding C2-alkylated derivative in 84% isolated yield (Scheme 25).

The mechanism proposed by the authors matches with the *path a* described above in the Scheme 3, relying on an olefin insertion/acidolysis sequence. H/D exchange experiments confirmed that step **II** follows a CMD process, and investigation of the KIE suggested that it is the rate-limiting step of the reaction.

**Scheme 26:**
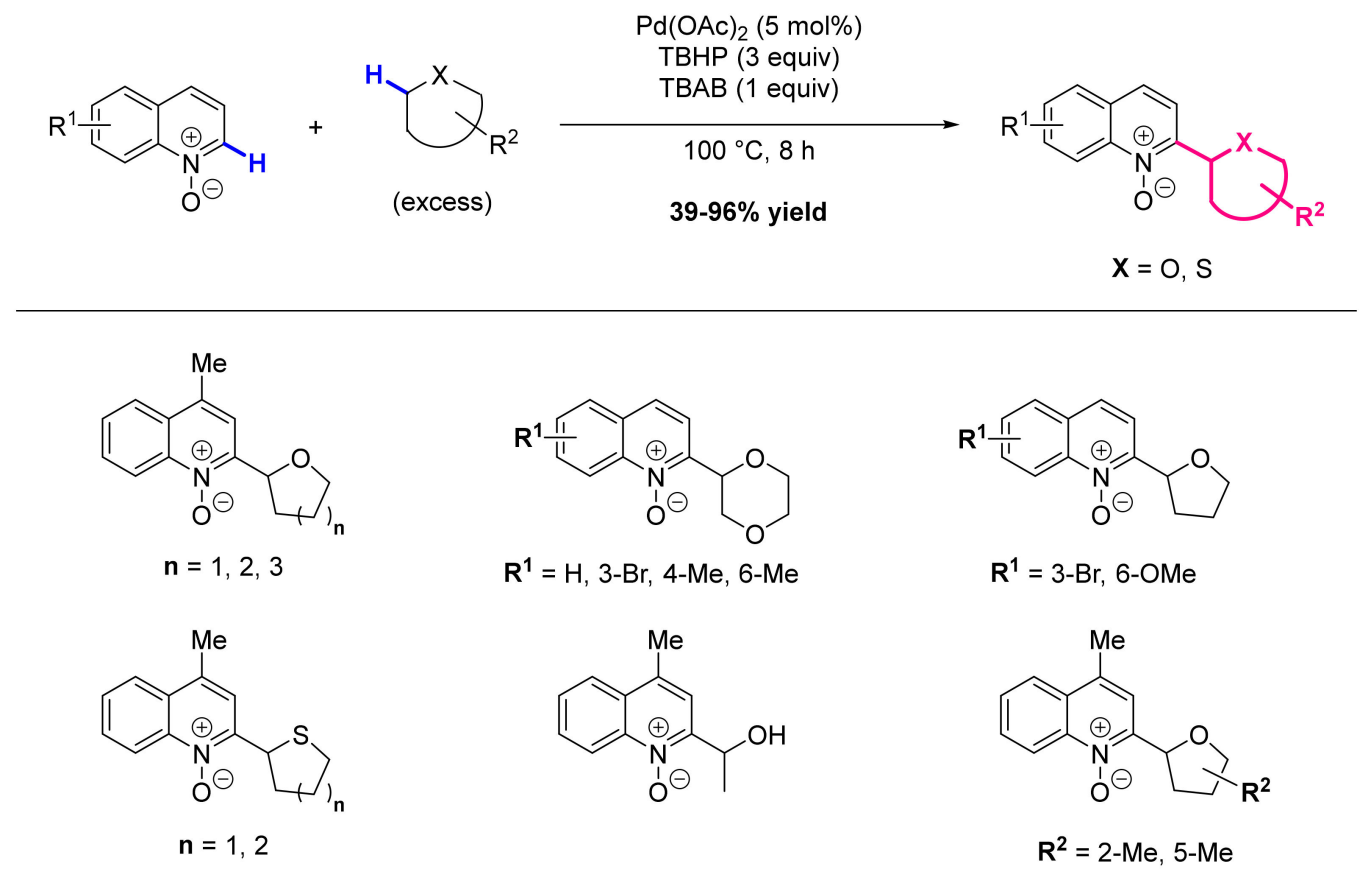
Pd-catalyzed C2 oxidative cross-coupling of quinoline N-oxides with heterocycles.

In 2013, Cui et al. developed an efficient direct alkylation of quinoline *N*-oxide with ethers via a palladium-catalyzed dual C-H bonds activation [132]. Using 4-methylquinoline *N*-oxide (1 equiv) and 1,4-dioxane/H_2_O (1.8/0.2 (v/v), 2 mL) as model substrates, a large screening of palladium catalysts, oxidants and salts revealed that Pd(OAc)_2_ (5 mol%), 70% *tert*-butylhydroperoxide in water (3 equiv) and TBAB (1 equiv), under air at 100 °C for 8 h, provided the *N*-alkylated QNO in an excellent 96% yield. In this reaction, the oxidant was shown to play a paramount role. The scope of the reaction was then studied on 4-methyl-, 6-methyl-, 6-methoxy- and 3-bromoquinoline *N*-oxides with ethers and thioethers displaying various sizes. A panel of 14 C2 alkylated products was isolated in moderate to excellent yields (Scheme 26).

The mechanism proposed by the authors corresponds to the *path a* described above in the Scheme 3, relying on the addition of a dioxane radical, which is generated *in situ* from the reaction of dioxane with TBHP, on the C2-metallated species **C** (step **III**), followed by the RE step **IV** to afford the desired product and to regenerate the Pd(II)-catalyst.

**Scheme 27:**
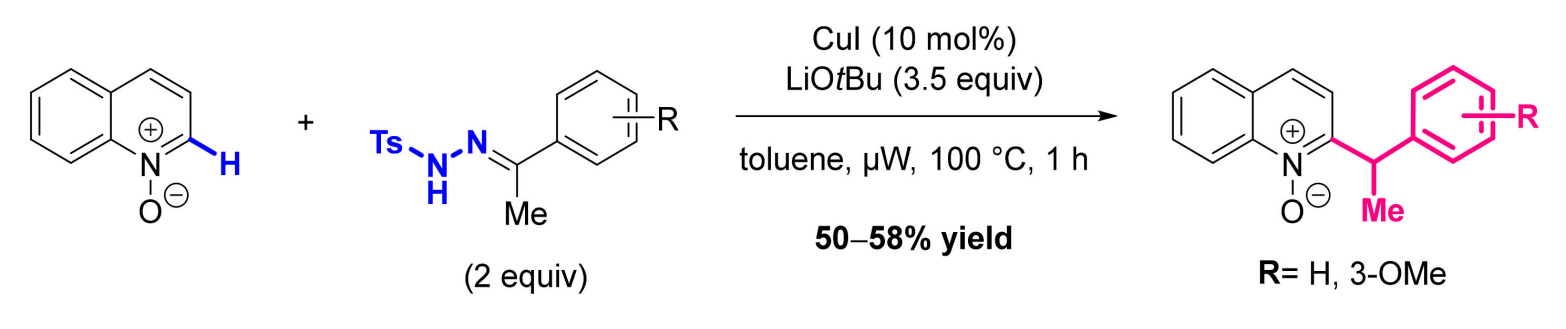
Cu-catalyzed C2 alkylation of quinoline *N*-oxide with tosylhydrazones.

In 2016, Jain et al. reported the first Cu-catalyzed microwave-assisted C2 alkylation of azine *N*-oxides, including the quinoline *N*-oxide, with tosylhydrazones [133]. This reaction, catalyzed by copper(I) iodide, revealed to be an efficient and selective route towards the synthesis of both primary and secondary C2-alkyl-substituted azines. Optimization of the reaction conditions towards the coupling of pyridine *N*-oxide (1 equiv) with the *N*-tosylhydrazone of acetophenone (2 equiv) was carried out with respect to the copper catalyst, base, solvent, time and temperature of the reaction. The best identified conditions for this reaction involved the irradiation of the substrates under µW at 100 °C for one hour using CuI (10 mol%) and LiO*t*Bu (3.5 equiv) in toluene, and gave the 2-alkylated pyridine in 77% yield. The scope of *N*-tosylhydrazones, derived from variously substituted aldehydes and ketones, was explored. The reaction was also successfully applied to the quinoline *N*-oxide as a substrate that reacted with the tosyl hydrazones of methylphenyl and methyl-3-methoxyphenyl ketones to give the corresponding C2-alkylated QNO derivatives in 50 and 58% yield, respectively (Scheme 27).

The mechanism proposed by the authors is similar to the *path a* described above in the Scheme 3, involving the reaction of the C2-Cu organometallic species **C** with a diazo compound (step **III**), which was generated *in situ* from *N*-tosylhydrazone treated with LiO*t*Bu, to form the corresponding copper-carbene as key intermediate **D**. From there, migratory insertion of the alkenyl group onto the carbenic carbon and subsequent protonolysis (step **IV**) yields the desired product and regenerates the catalyst. Experimental investigations carried out with deuterated substrates excluded that step **II** could be the rate-determining step.

### 2.5. Formation of C-C(O)NHR Bond

**Scheme 28:**
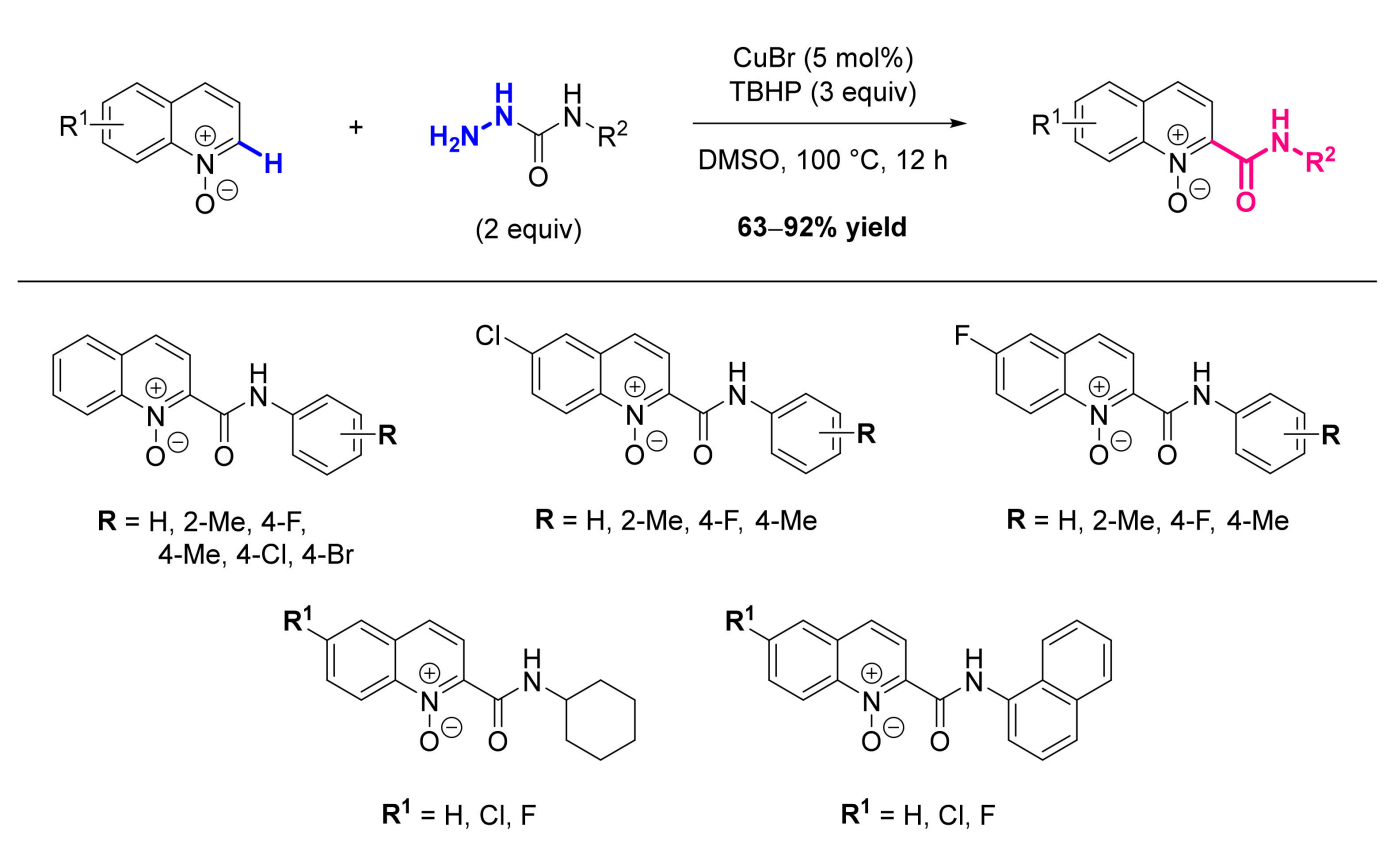
Cu-catalyzed C2 carbamoylation of quinoline *N*-oxides with hydrazinecarboxamides.

Single example in this category, the direct copper-catalyzed carbamoylation of quinoline *N*-oxides with hydrazinecarboxamides was reported by Wang et al. in 2019 [134]. The conditions of the reaction were optimized with quinoline *N-*oxide (1 equiv) and *N*-phenylhydrazinecarboxamide (2 equiv) as model substrates. The screening of various copper catalysts revealed that CuBr was the most efficient while palladium ones were unsuccessful. The best conditions involved CuBr (15 mol%), TBHP (3 equiv) in DMSO under air for 12 h at 100 °C, and led to the C2-carbamoyl derivative of quinoline *N*-oxide in 92% yield. The scope of the hydrazinecarboxamides was then investigated, with substrates containing both EWGs and EDGs on the benzene ring, leading to a panel of 5 new products in yields ranging from 75 to 81%. The reaction was also shown to be compatible with substituted QNOs (6-Cl- and 6-F-), giving 8 C2-carbamoylated products in good yields (65–90%). *N-*cyclohexyl hydrazinecarboxamide and *N*-(naphthalen-1-yl) hydrazinecarboxamide were also well tolerated, providing the corresponding products in moderate to high yields. However, 6-methoxyquinoline *N*-oxide, 6-methylquinoline *N*-oxide, 3-bromoquinoline *N*-oxide and 6-(methoxycarbonyl)quinoline *N*-oxide proved to be unreactive under these conditions (Scheme 28).

The mechanism proposed by the authors matches with the *path a* described above in the Scheme 3, involving the addition of a carbamoyl radical, which was generated *in situ* from the hydrazinecarboxamide treated with TBHP and Cu, on the C2-metallated species **C** (step **III**), followed by the RE step **IV** to yield the desired product and close the catalytic cycle. The existence of a free radical intermediate was supported by control experiments that were carried out in the presence of radical trapping reagents (di-*tert*-butylhydroxytoluene—BHT and 2,2,6,6-tetramethylpiperidinyl-1-oxy—TEMPO).

### 2.6. Formation of C-N Bond

The formation of a C-*N* bond at the C2 position of quinolines is limited to quinoline *N*-oxides substrates in the reported methods, only the use of various copper catalysts revealed efficient for the reaction, the tested palladium catalysts being either inefficient or only leading to traces of the expected product. In most of these methods, the 2-aminoquinoline *N*-oxides could efficiently be reduced by PCl_3_ in excellent yields.

**Scheme 29:**
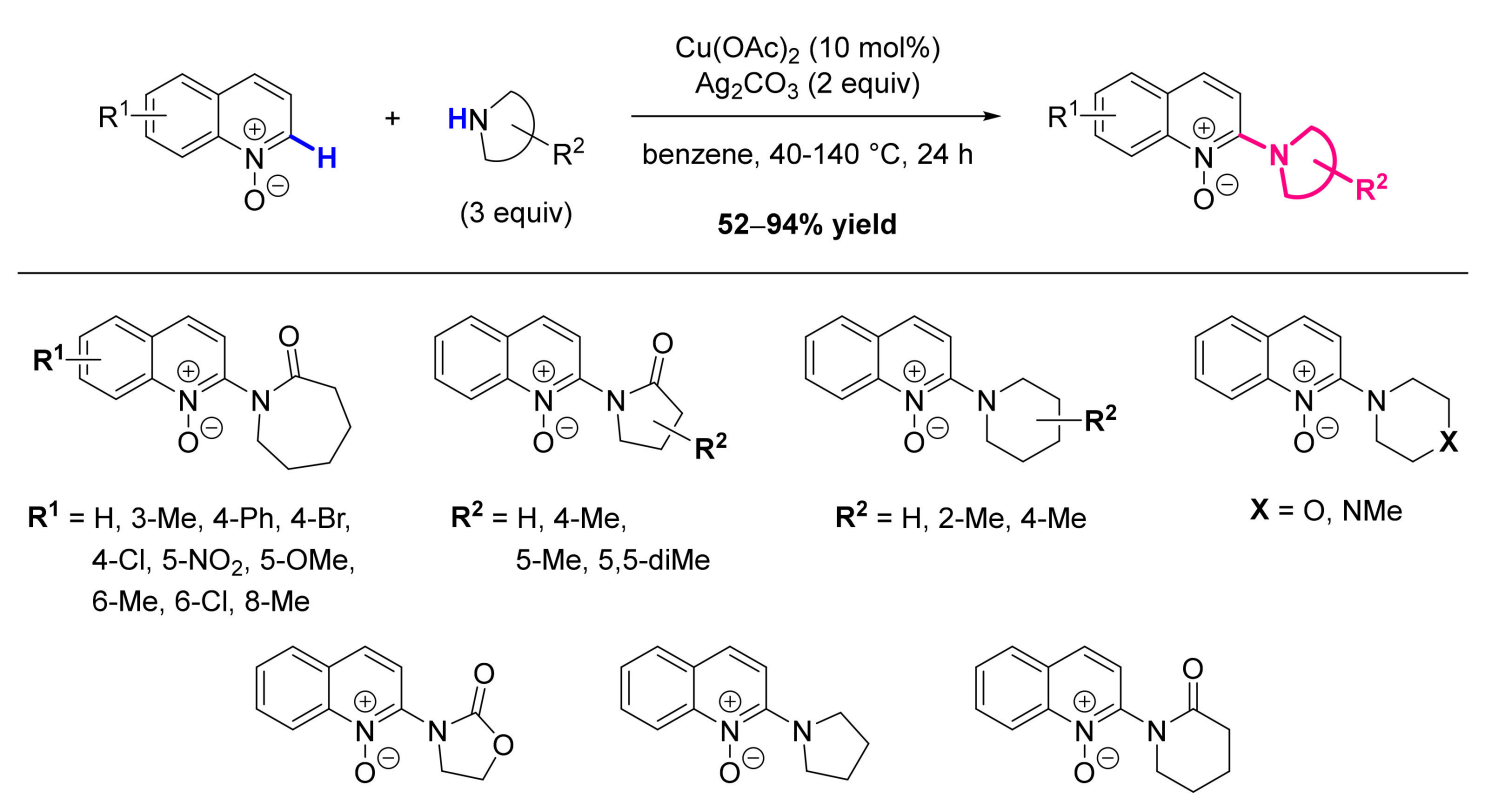
Cu-catalyzed C2 amination/amidation of quinoline *N*-oxides with amines or amides.

In 2013, Li et al. described a copper-catalyzed intermolecular dehydrogenative amination/amidation of quinoline *N*-oxides with lactams/cyclamines to construct 2-aminoquinolines [135]. The conditions of the reaction were optimized with quinoline *N*-oxide (1 equiv) and hexanolactam (3 equiv) as model substrates. Screening of various copper catalysts, oxidants and solvents revealed that the best conditions require Cu(OAc)_2_ (10 mol%), Ag_2_CO_3_ (2 equiv) in benzene at 120 °C for 24 h to afford the the C2 aminated quinoline *N*-oxide in an excellent 93% yield. The scope of the reaction was investigated regarding both substrates, leading to a panel of 22 C2-*N*-substituted QNOs that were obtained in yields ranging from 52 to 94%. QNOs substituted by alkyl and aryl groups in different positions, including a methyl group at C3, led to the products in excellent yields. Strong EWGs and EDGs were well tolerated, as well as halogens, giving the expected products in high yields. Next, lactams with various sizes, 2-pyrrolidone with methyl substituents in different positions and 2-oxazolinone were found to be excellent partners for the reaction. Piperidine, pyrrolidine, and methylpiperidine exhibited excellent reactivities as well as morpholine and piperazine (Scheme 29).

The mechanism proposed by the authors corresponds to the *path a* described above in the Scheme 3, involving the Ag_2_CO_3_-mediated oxidation of the key intermediate **D** from Cu(II) to Cu(III) complex, followed by the RE step **IV** to furnish the desired product, plus another Ag-mediated oxidation to close the catalytic cycle. Deuterium-labelling experiments showed that the C-H bond cleavage (step **II**) is not involved in the rate-limiting step.

**Scheme 30:**
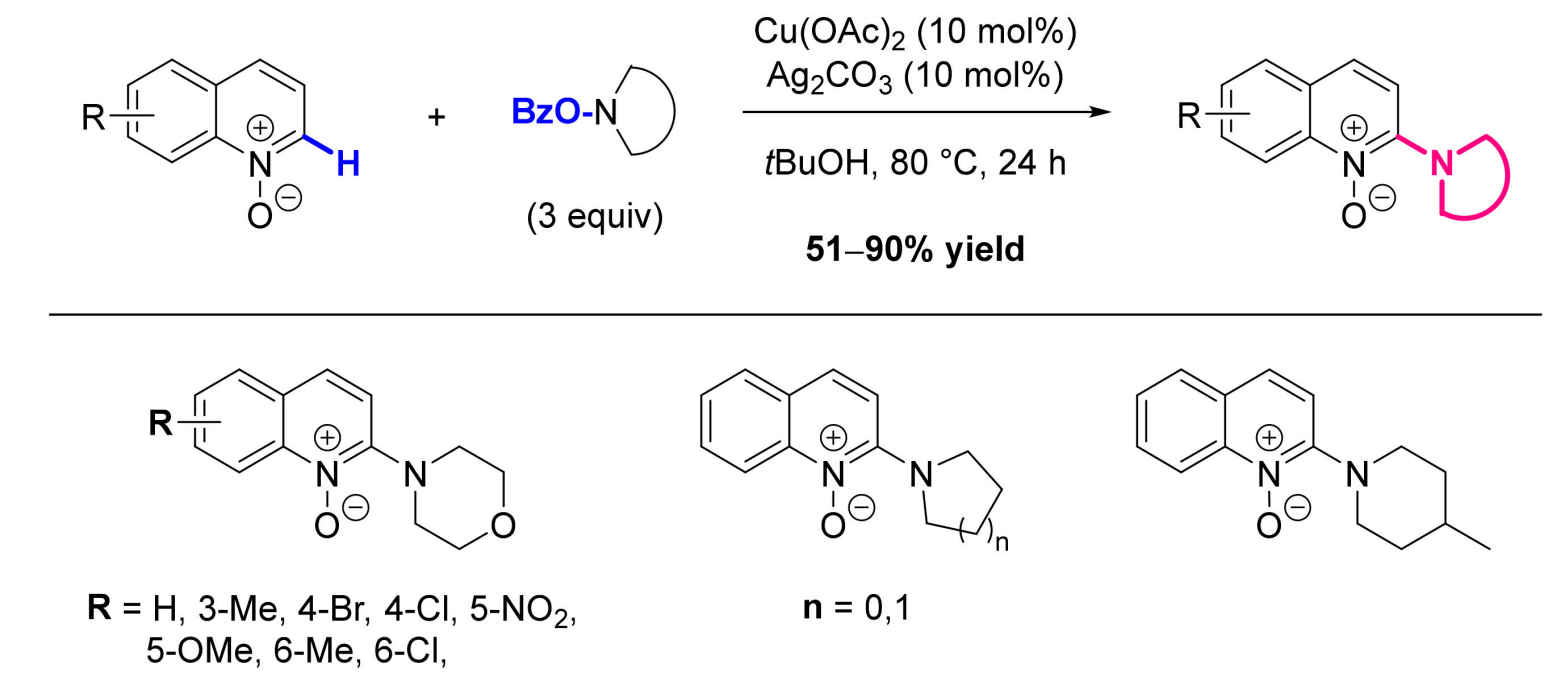
Cu-catalyzed C2-amination of quinoline *N*-oxides with *O*-benzoyl hydroxylamines.

Later on, in 2015, Li and Wu developed a copper(II)-catalyzed electrophilic amination of quinoline *N-*oxides with *O*-benzoyl hydroxylamine as the electrophilic amination reagent [136]. For the transformation of quinoline *N*-oxide (1 equiv) and *O*-benzoyl hydroxylamine (3 equiv), used as model substrates, the screening of catalysts, additives and solvents revealed that, as in the previous example, Cu(OAc)_2_ (10 mol%) and Ag_2_CO_3_ (10 mol%) were superior to other copper catalysts and carbonates or phosphate additives. Under these optimized conditions in *tert-*BuOH at 80 °C for 24 h, the C2-aminated derivative was isolated in 77% yield. The scope of this transformation was then explored. Substitution of quinoline *N*-oxide with an alkyl at C6, or even at C3, gave the expected products in excellent yields while its substitution at C5 by either an EWG or an EDG led to the products in moderate yields. Interestingly, halogen substituents, suitable for further functionalization, were tolerated, therefore delivering the C2-aminated compounds in moderate to good yields. *O*-benzoyl hydroxylamines displaying various sizes and substitution patterns gave the corresponding C2-aminated derivatives in moderate yields (Scheme 30).

The mechanism proposed by the authors corresponds to the *path a* described above in the Scheme 3, relying on a standard OA/RE sequence. Measurement of the intermolecular deuterium KIE suggested that the C-H bond activation in QNO is not involved in the rate-limiting step.

**Scheme 31:**
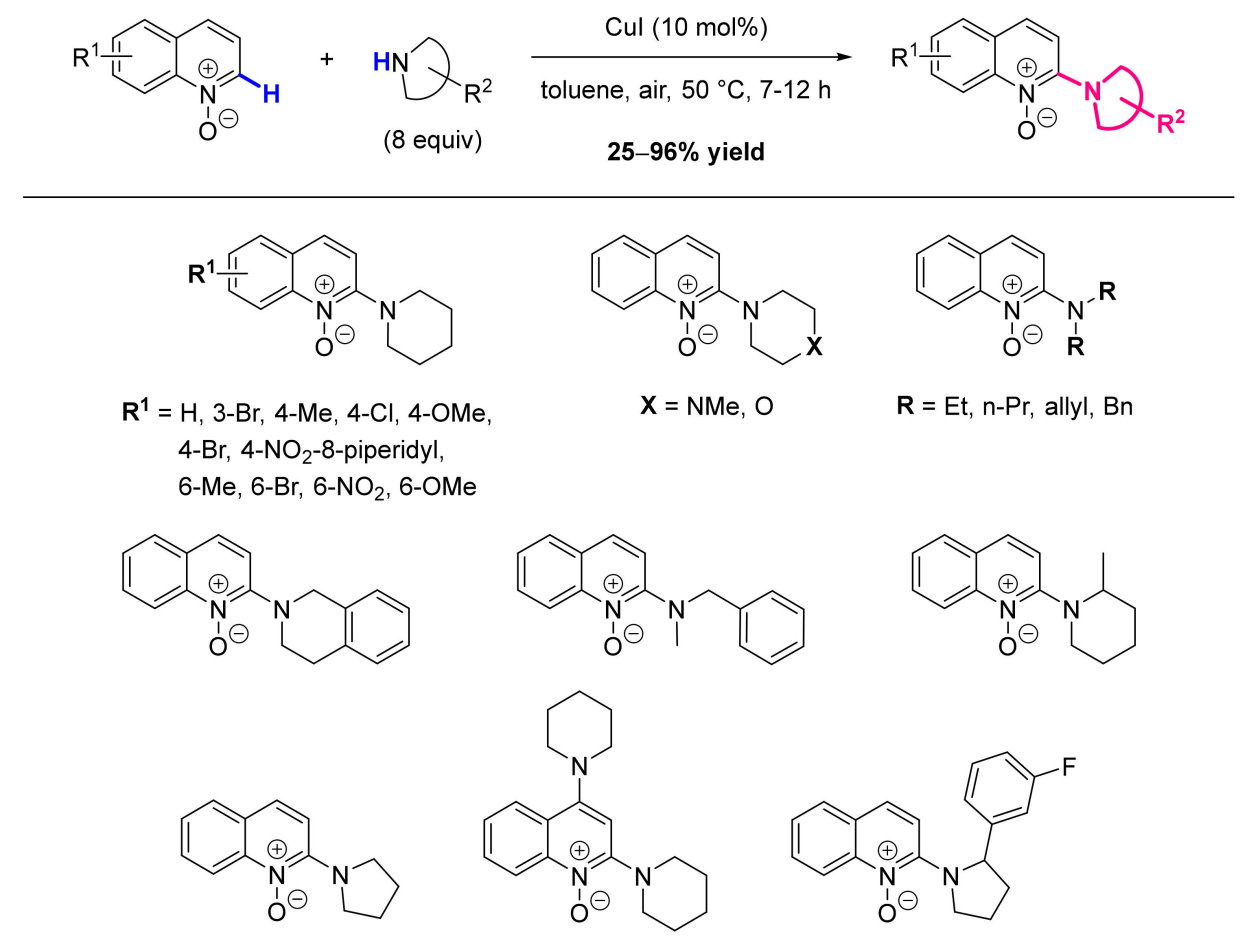
Cu-catalyzed C2 amination of quinoline *N*-oxides with aliphatic secondary amines.

In 2014, Wu and Cui described the direct amination of quinoline *N*-oxides via copper-catalyzed dehydrogenative C−*N* coupling with aliphatic secondary amines [137]. Interestingly, the reported conditions are ligand, additives, base, and external oxidant free. They were optimized regarding the catalyst, the solvent and the loading of catalyst and amine. In the best ones, involving quinoline *N*-oxide (1 equiv) and an excess of piperidine (8 equiv) as substrates, the C2 aminated product was isolated in 91% yield in the presence of CuI (10 mol%) in toluene at 50 °C under air. The scope of the reaction was investigated regarding both substrates. Cyclic amines gave the expected products in moderate to good yields. Acyclic secondary aliphatic amines, either symmetric or dissymmetric, could also be used. However, diallylamine and dibenzylamine gave poor yields that might be due to their lower nucleophilicity. It was shown that QNO substitution by EDGs and EWGs did not display significant influence on the transformation, the desired products being isolated in excellent yields. 4-bromoquinoline *N*-oxide gave a mixture of monoaminated (65% yield) and diaminated (31% yield) products. For 4-nitroquinoline *N*-oxide as a substrate, 2,8-diaminated quinoline *N*-oxide was isolated in 80% yield. However, the presence of the -OMe electron-donating group at position 6 of QNO only gave the desired product a modest 27% yield, probably due to the reduced reactivity of C2-H bond (Scheme 31).

The mechanism proposed by the authors corresponds to the *path a* described above in the Scheme 3, and is very similar to the Cu(I)/Cu(III) process described by Li et al. for the previous example, except that in this case O_2_ serves as the oxidant instead of Ag_2_CO_3_. Control experiments conducted under nitrogen atmosphere evidenced the role of O_2_ and DFT calculations supported the overall mechanism.

**Scheme 32:**
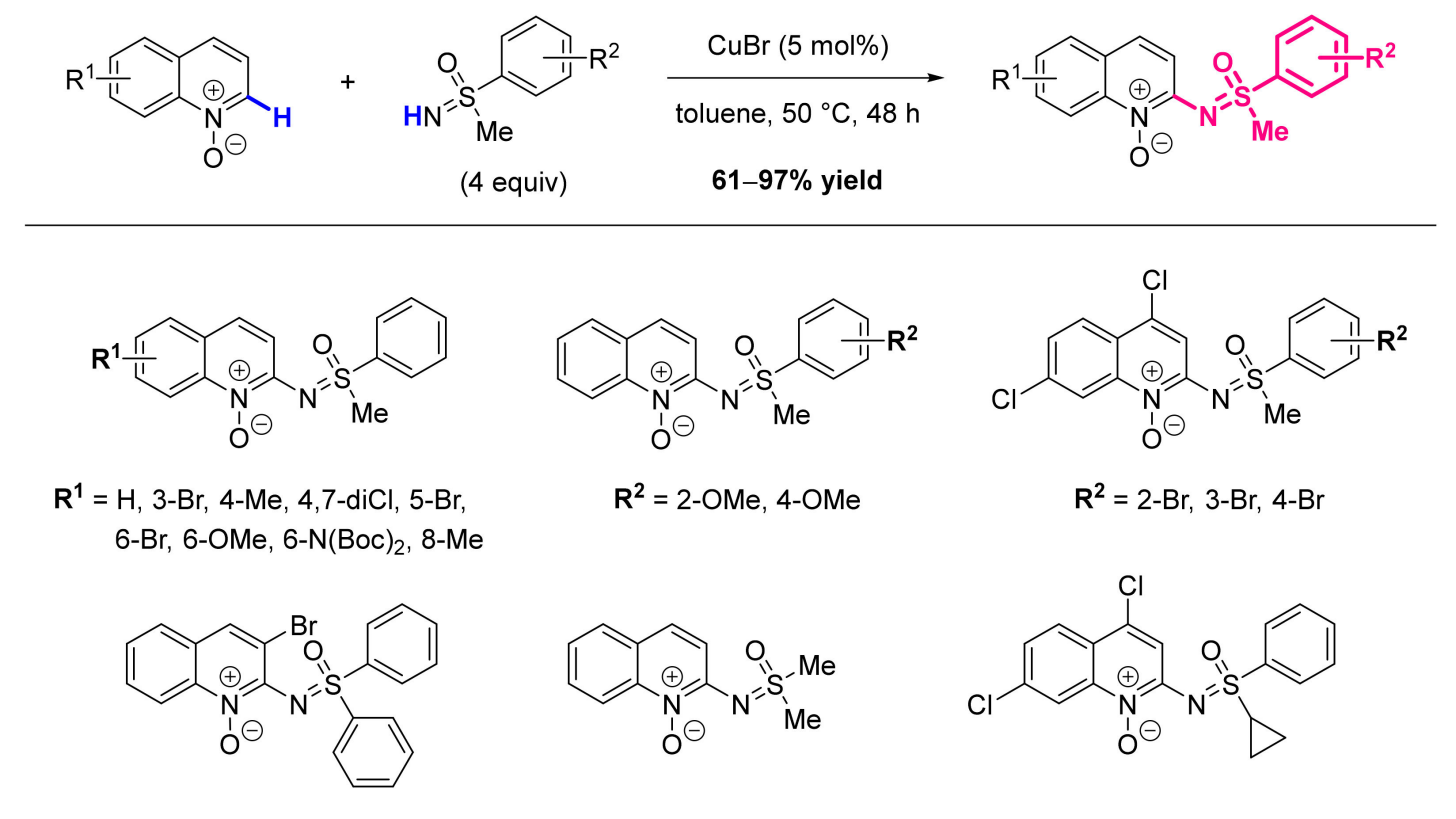
Cu-catalyzed C2 sulfoximination of quinoline-*N*-oxides with sulfoximines.

In 2016, Bolm et al. described the copper-catalyzed direct sulfoximination of quinoline-*N*-oxides by dual C-H/N-H dehydrogenative coupling with sulfoximines [138]. Among the screened copper catalysts for the reaction of quinoline *N*-oxide (1 equiv) and S-methyl-S-phenylsulfoximine (4 equiv), CuBr (5 mol%) in toluene at 50 °C for 48 h proved to be the most efficient and afforded the C2-functionalized QNO derivative in 96% yield. The reaction was performed in air and no additional ligand, base or oxidant was required. QNOs with various substituents led to the expected products in high to excellent yields, showing that neither the substitution pattern nor the electronic effects induced by substituents played a major role. Bulky substituents at neighboring carbons did not have a negative effect on the reaction. Quinoline *N-*oxide, 4,7-dichloro-quinoline *N-*oxide and 3-bromo-quinoline *N-*oxide also reacted with various sulfoximines in moderate to good yields (Scheme 32).

The mechanism proposed by the authors corresponds to the *path a* described above in the Scheme 3, involving the aerobic oxidation of the key intermediate **D** from Cu(I) to Cu(III) complex, prior to the RE (step **IV**) that provides the desired product and regenerates the catalyst. Deuterium-labelling experiments showed that the C-H bond cleavage (step **II**) is not involved in the rate-limiting step.

**Scheme 33:**
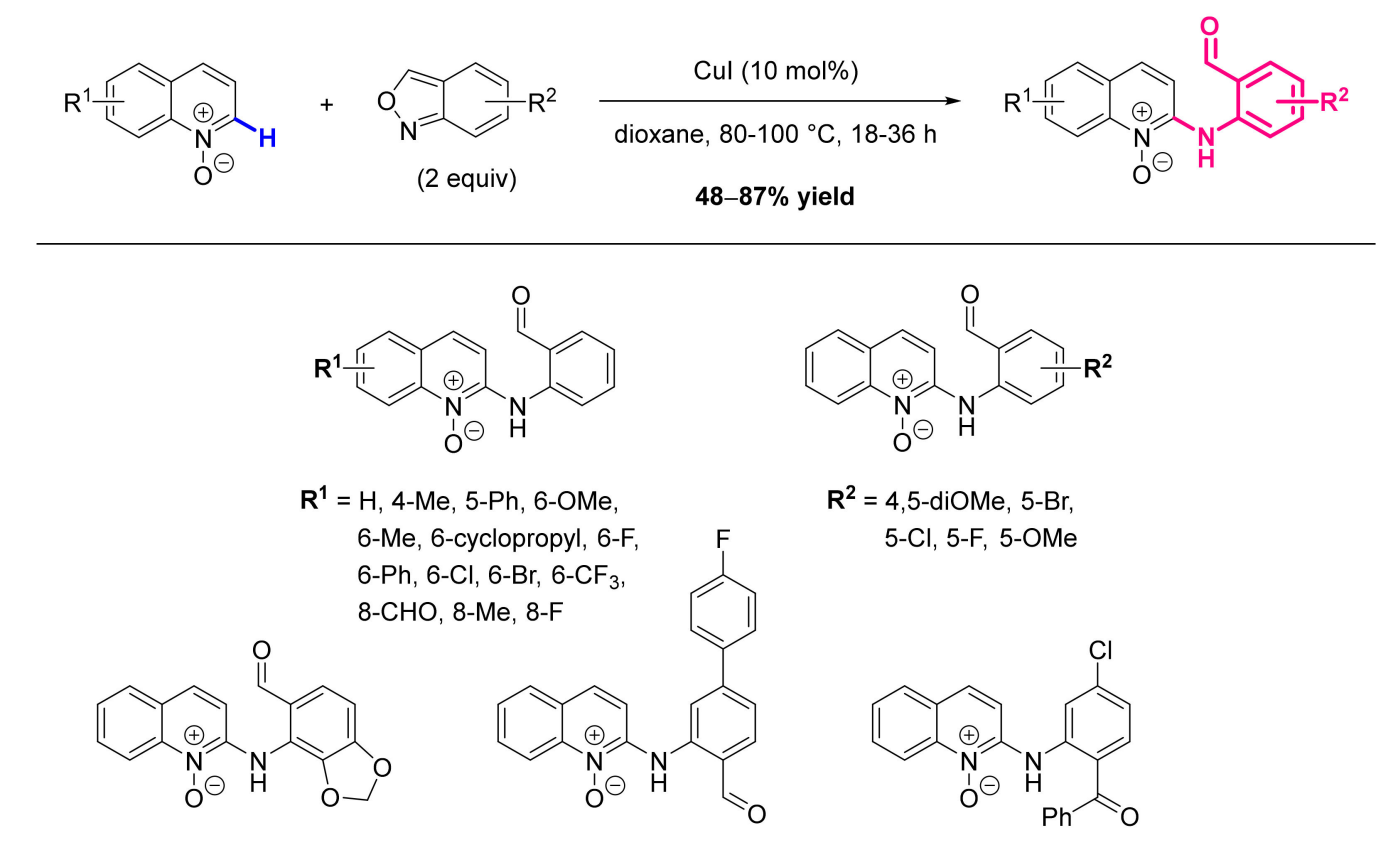
Cu-catalyzed C2 arylamination of quinoline *N*-oxides with anthranils.

More recently, in 2017, Samanta et al. reported the copper-catalyzed regioselective arylamination of various heterocyclic *N-*oxide under redox-neutral conditions using anthranils as arylaminating reagents [139]. The conditions for this reaction were optimized with quinoline *N*-oxide (1 equiv) and anthranil (1.2 equiv) as substrates, and screening of various copper catalysts showed that CuI (10 mol%) in 1,4-dioxane at 100 °C for 36 h afforded the C2-*N*-arylated quinoline *N*-oxide in the best yield (89%). The scope of both substrates was investigated. Electronically and sterically variable functional groups at the C6 position of QNO were well tolerated. Substitutions at the C5 or C4 positions were also possible, while alkyl and halogen groups were compatible at the C8 position. However, the sensitive acetal group was cleaved during the reaction and gave the corresponding product with an aldehyde group. Several anthranil compounds were also successfully engaged in the reaction leading to a panel of 22 C2-arylaminated QNOs (Scheme 33). Noteworthy, the dual functional groups in the final molecules were exploited to construct nitrogen-containing polyaromatic hydrocarbons via a series of post synthetic modifications.

To get insights into the mechanism, control experiments in the presence of 3,5-di-*tert*-4-butylhydroxytoluene (BHT) as radical quencher excluded the possibility of a radical pathway. Moreover, the absence of KIE, neither in parallel nor in competitive experiments, led to the conclusion that the C-H bond cleavage in QNO does not occur during the rate-determining step. Nevertheless, two different mechanisms remain practicable and thus the reader is invited to consult the original article for more information if desired.

### 2.7. Formation of C-S Bond

**Scheme 34:**
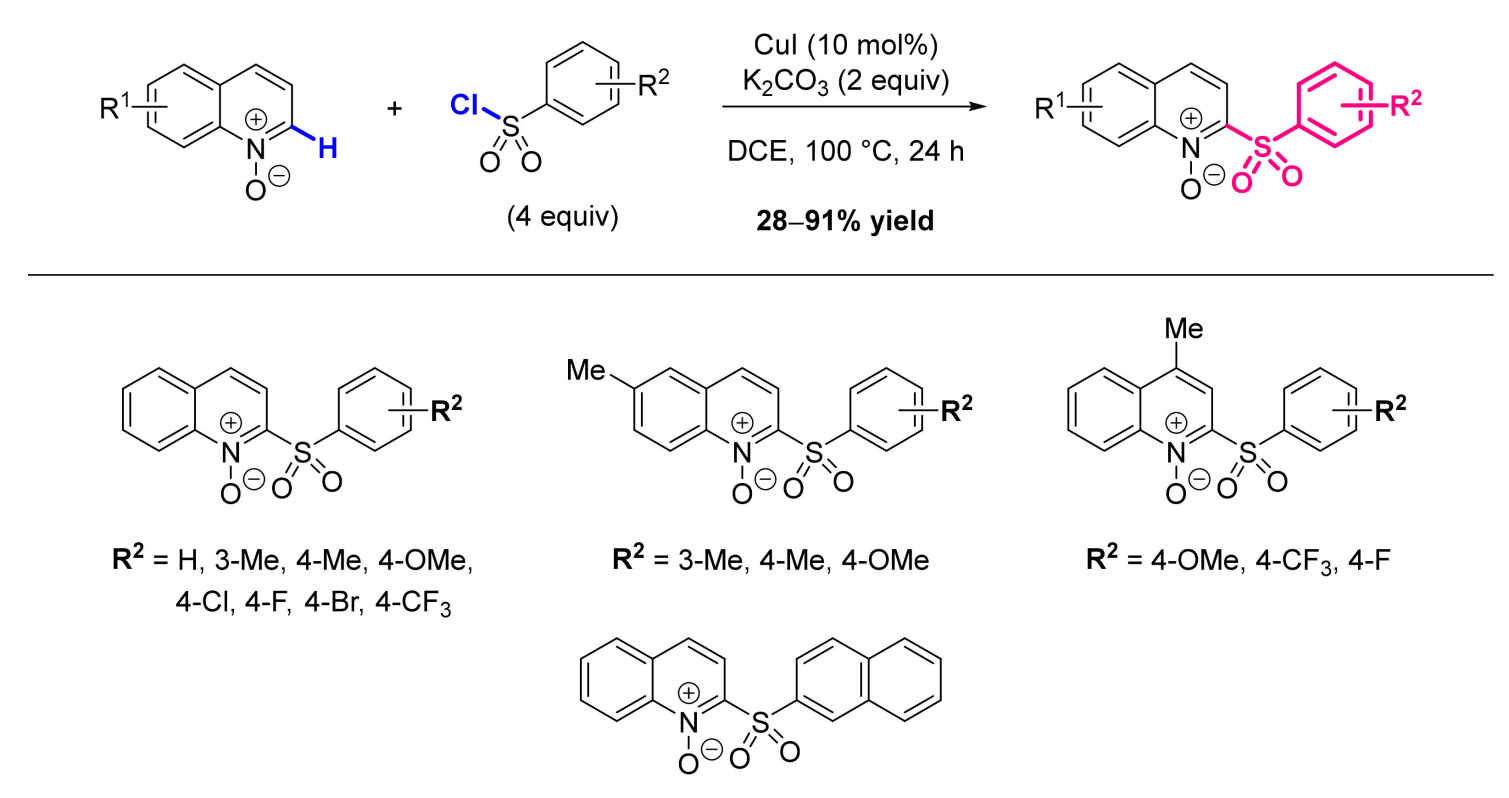
Cu-catalyzed C2 sulfonylation of quinoline *N*-oxides with sulfonyl chlorides.

Wu and colleagues developed successively two Cu-based methods to form C-S and C-Se bonds. The first one, reported in 2013, allows the sulfonylation of quinoline *N*-oxides with aryl sulfonyl chlorides [140]. The conditions of this reaction were optimized with quinoline *N*-oxide (1 equiv) and *p*-tolylsulfonyl chloride (4 equiv) as model substrates for which a panel of catalysts and bases were screened. In the best conditions involving CuI (10 mol%) and K_2_CO_3_ (2 equiv) in DCE at 100 °C for 24 h under air, the C2-sulfonylated quinoline *N*-oxide derivative was isolated in 91% yield. The scope of the reaction was studied with various QNOs and diversely substituted aryl sulfonyl chlorides as substrates. QNO derivatives with electron-donating methyl groups at the 4- and 6-positions provided the expected C2-sulfonylated products in moderate to good yields. Both EDGs and EWGs on the sulfonyl chloride revealed to be compatible with the reaction conditions leading to the products in moderate to excellent yields. Noteworthy, the electron donating ones proved to be slightly more efficient than the others (Scheme 34).

The mechanism proposed by the authors matches with the *path a* described above in the Scheme 3, relying on a standard OA/RE sequence.

**Scheme 35:**
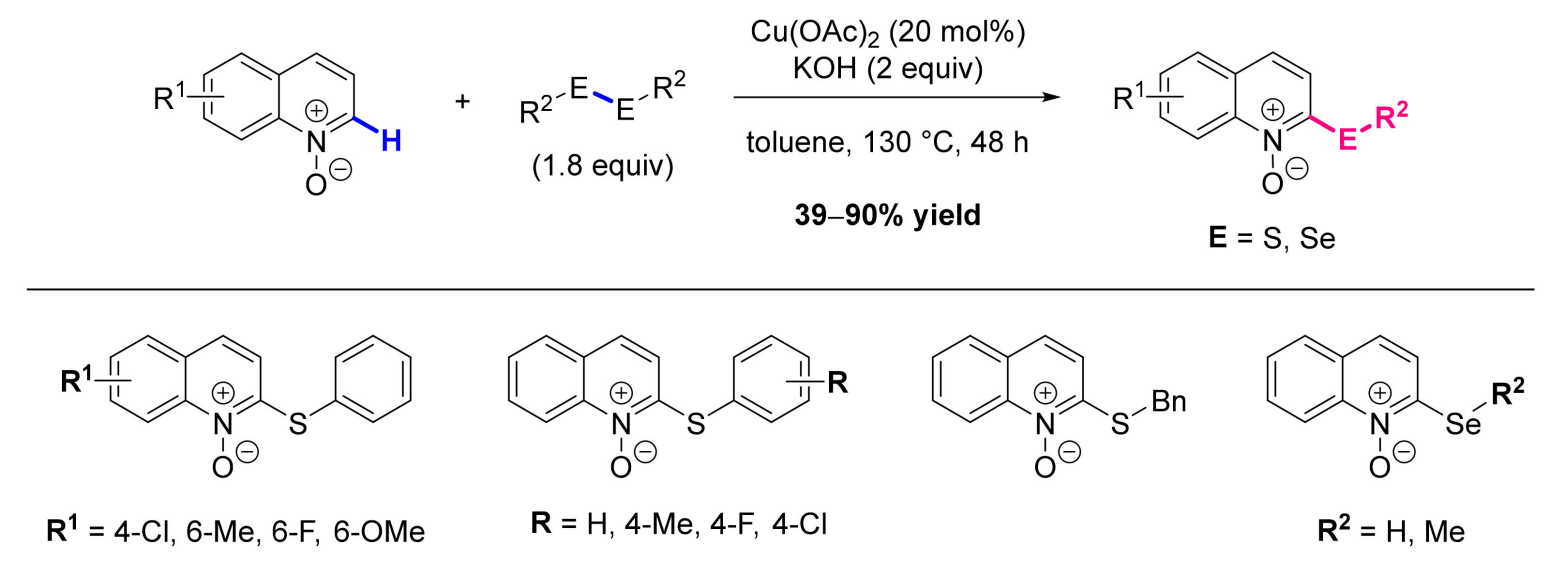
Cu-catalyzed C2 thiolation of quinoline *N*-oxides with disulfides.

The second method is the copper-catalyzed regioselective thiolation of aza-heteroaromatic *N*-oxides with bisulfides [141]. The conditions of this transformation were optimized regarding the stoichiometry of the reactants, the copper catalyst, the base and the solvent. In the best identified conditions, quinoline *N*-oxide (1 equiv) and diphenyl disulfide (1.8 equiv) were used as model substrates, and were reacted in the presence of Cu(OAc)_2_ (20 mol%) and KOH (2 equiv) in toluene at 130 °C for 48 h to afford the C2-thiolated derivative of quinoline *N*-oxide in 80% yield. The scope of the reaction was studied and various QNOs and disulfides were screened. A panel of nine products coming from four diversely substituted QNOs and phenyl or benzyl disulfides was obtained in moderate to good yields. It was observed that diphenyl disulfide with EDGs gave higher yields than those having EWGs. Diphenyl and dimethyl diselenide were compatible with the conditions of the reaction, giving the corresponding products in 90 and 60% yield, respectively. (Scheme 35).

The mechanism proposed by the authors corresponds to the *path a* described above in the Scheme 3, involving the addition of a thiyl radical on the C2-metallated species **C** (step **III**), followed by RE (step **IV**) and reoxidation of Cu(I) into Cu(II) to yield the desired product and close the catalytic cycle. The existence of a radical intermediate was evidenced by experiments that were carried out in the presence of radical scavenger such as TEMPO, BHT or galvinoxyl free radical.

**Scheme 36:**
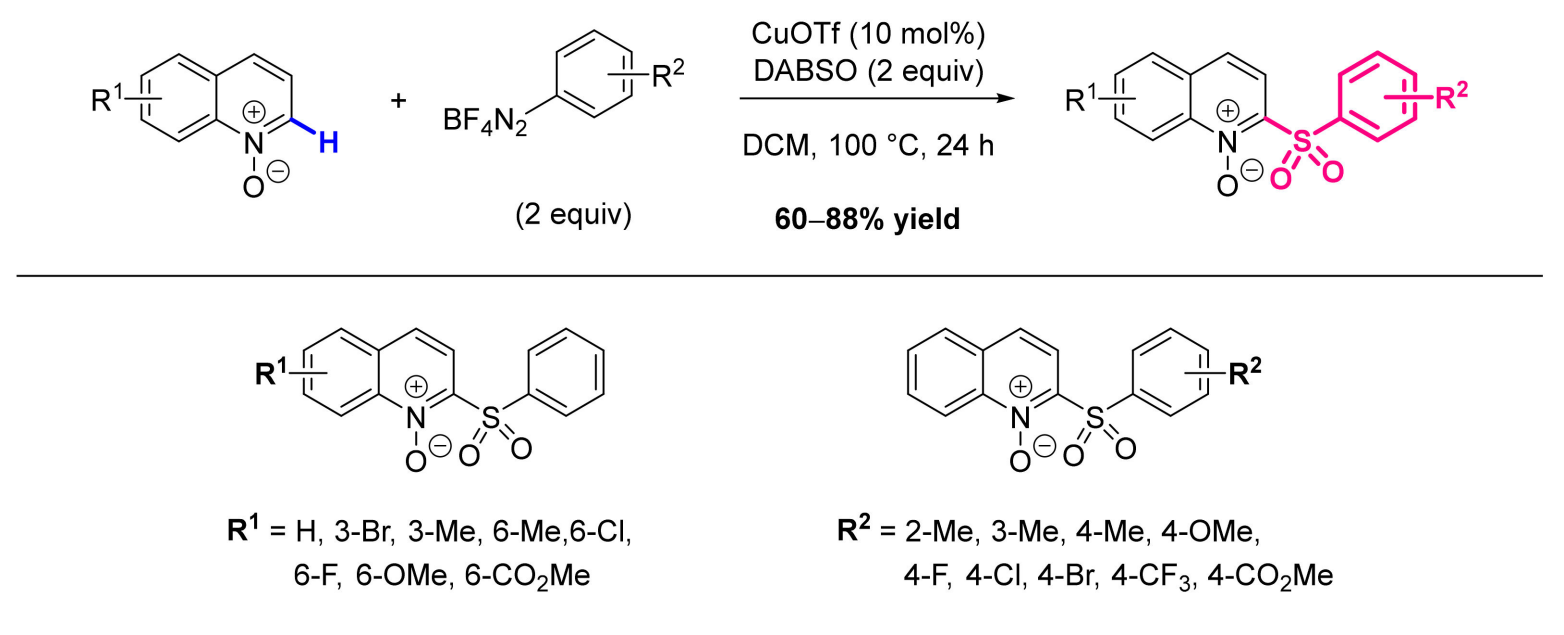
Cu-catalyzed C2 sulfonylation of quinoline *N*-oxides with tetrafluoroborates.

More recently, C2-sulfonylation of QNOs with 1,4-diazabicyclo[2.2.2]octane bis(sulfur dioxide) (DABSO) and phenyldiazonium tetrafluoroborates was described by the team of Wang [142]. In order to optimize the reaction, the effect of copper catalyst, solvent and temperature was examined and the use of CuOTf (10 mol%) in DCM at 100 °C appeared to be the most efficient. These conditions proved to be compatible with EWG- or EDG-substituted quinolines and aryl donors, leading to the synthesis of 18 examples in a yield range of 60 to 88% (Scheme 36).

The mechanism proposed by the authors corresponds to the *path a* described above in the Scheme 3, relying on the addition of a sulfonyl radical, which was generated *in situ* from the reaction of DABSO with the aryldiazonium tetrafluoroborate, on the C2-metallated species **C** (step **III**), followed by a deoxygenative elimination (step **IV**) to afford the desired product and to regenerate the Cu-based active species. The hypothesis of a free radical intermediate was supported by radical-trapping experiments.

## 3. Functionalization in Position 3

**Scheme 37:**
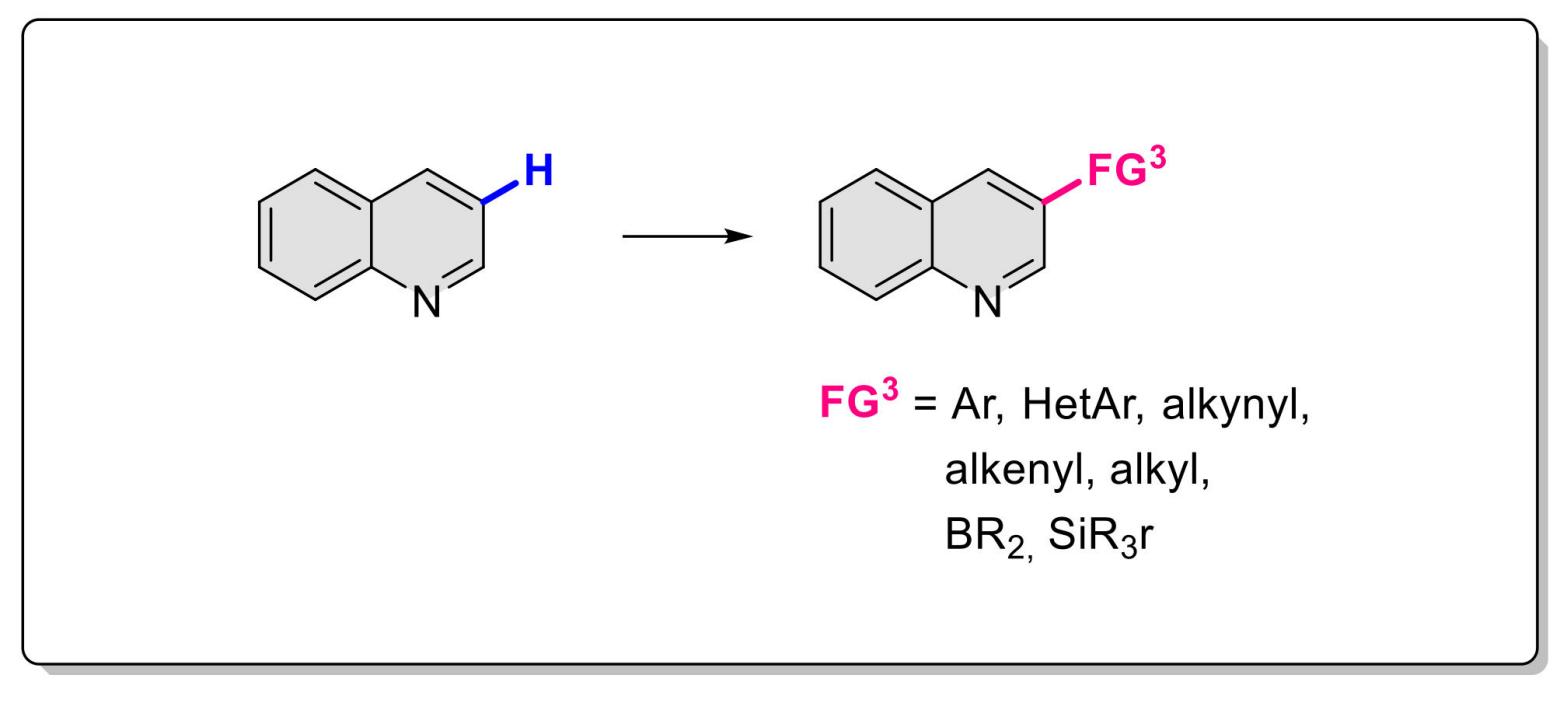
Scope of the selective C-H functionalization achieved in position 3 to date.

Reaching distal positions has still remained challenging in the field of C-H activation. However, a dozen of methods were developed to address the selective C3-H functionalization of quinolines to append aryl, heteroaryl, alkynyl, alkenyl, boronic and silano substituents (Scheme 37). All these reactions are starting from quinolines as substrates, and two main strategies are employed to get the desired regioselectivity: either the introduction of a directing group in an adjacent position (2 or 4), or the design of particular ligands. Once again, Pd seems to be the metal of choice to form C-C bonds while Ir-based catalysts are preferred in the case of C-heteroatom bonds.

### 3.1. Formation of C-Ar Bonds 

**Scheme 38:**
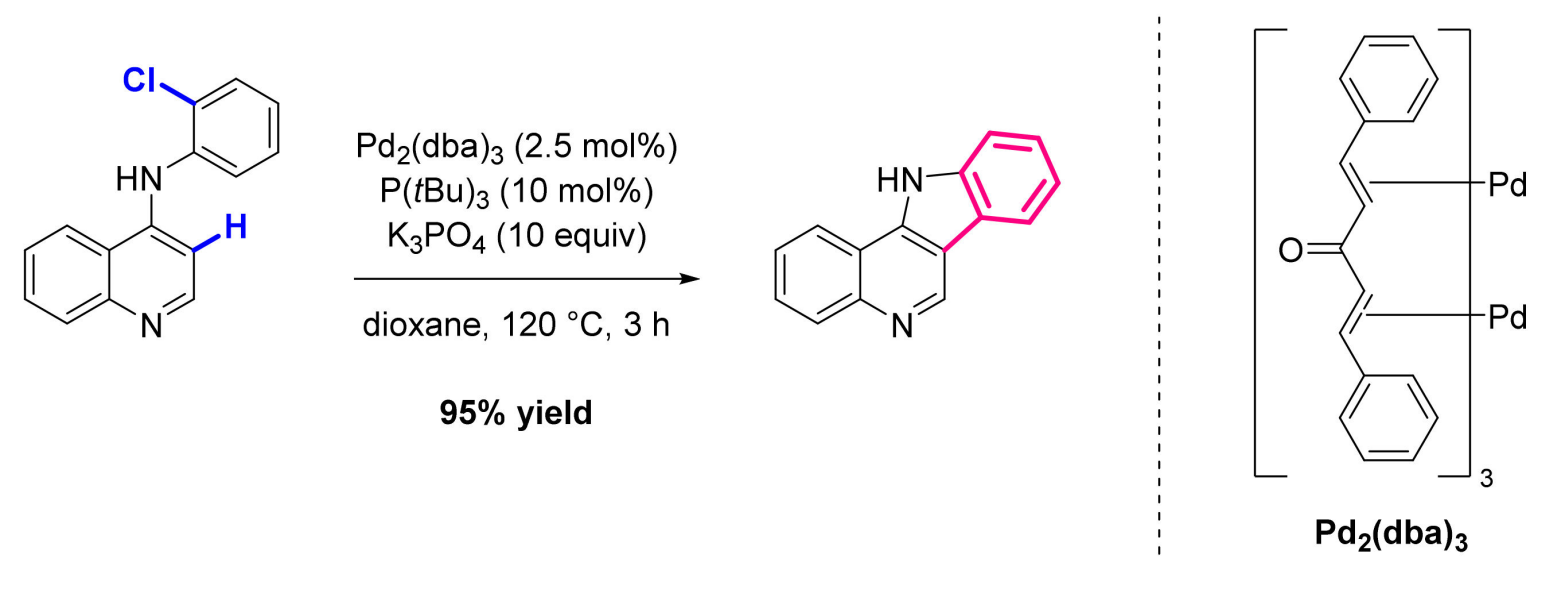
Intramolecular Pd-catalyzed C3 arylation of quinoline.

The first example of C3-H functionalization to form C-C bonds dates back to 2003, when the team of Maes described the total synthesis of natural alkaloid Isocryptolepine through a direct intramolecular C-H arylation of quinoline [143]. The screening of different ligands allowed the group to pass from a trace of product to a 53% yield, and the optimization of the reaction conditions (choice of the base, pressure and temperature) improved the yield up to 95% and decreased the reaction time from 72 h to only 3 h (Scheme 38).

**Scheme 39:**
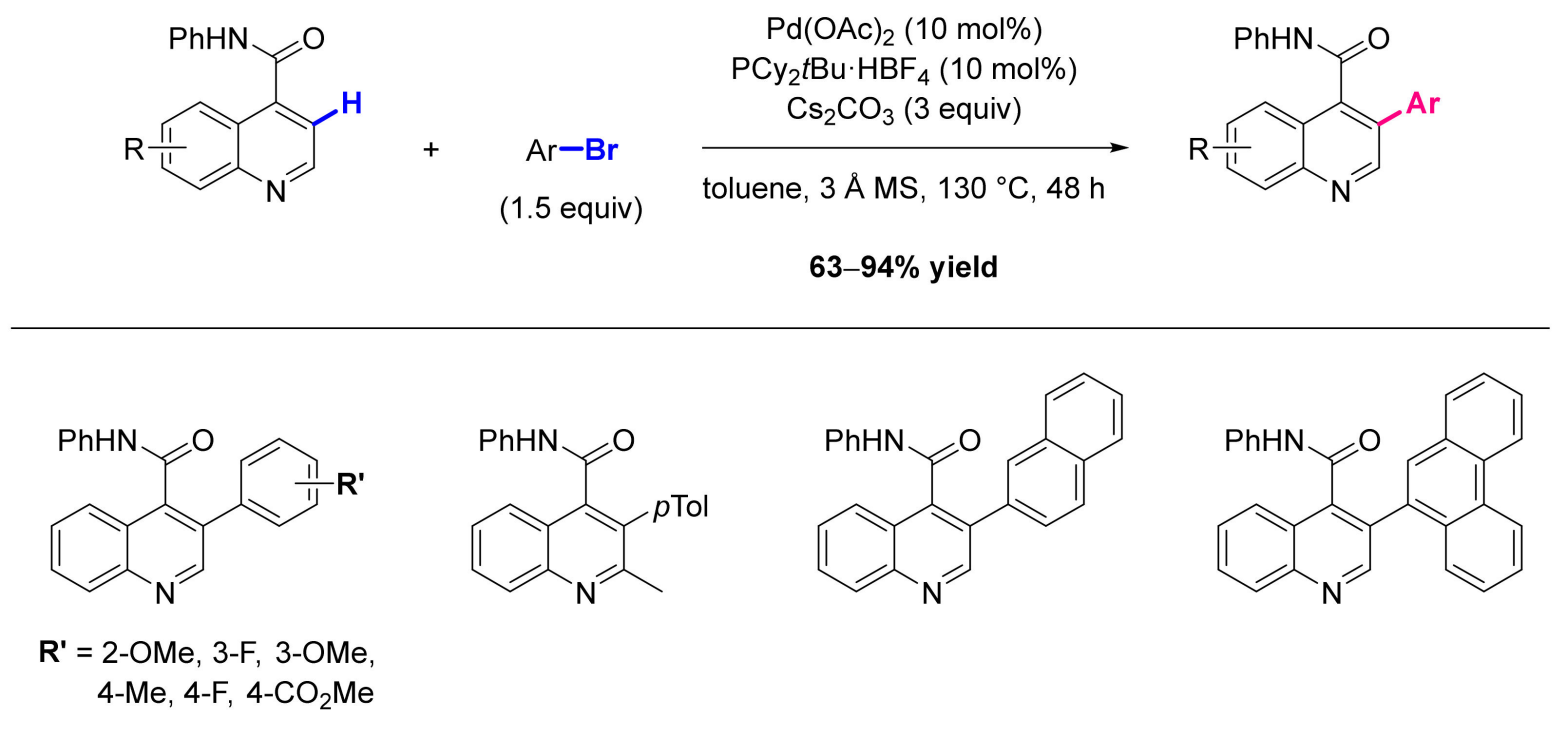
Pd-catalyzed DG-assisted C3 arylation of quinolines with aryl bromides.

In 2010 the team of Yu reported for the first time an intermolecular Pd-catalyzed C-H arylation, that is selective for the C3 position of both pyridines and quinolines bearing an *N*-aryl amide as directing group [144]. This method was optimized on a pyridine substrate through the screening of different ligands, which proved PCy_2_*t*Bu·HBF_4_ [145] as the most effective, and with the synthesis of different *N*-phenyl amides as NR-DG. The optimized conditions, using 10 mol% of Pd(OAc)_2_, 10 mol% of PCy_2_*t*Bu·HBF_4_ and 3 equivalents of Cs_2_CO_3_ in toluene, in the presence of molecular sieves and N_2_ for 48 h at 130 °C, were then applied to the quinoline substrates. QNs proved to be monoarylated selectively in position 3 with good overall yields ranging from 71% to 94%. The reaction tolerates EWGs, EDGs and fused aromatic rings. However, the reaction is hampered when the aryl bromide is substituted in *ortho* position and, as expected, no reaction was observed without the DG (Scheme 39).

**Scheme 40:**
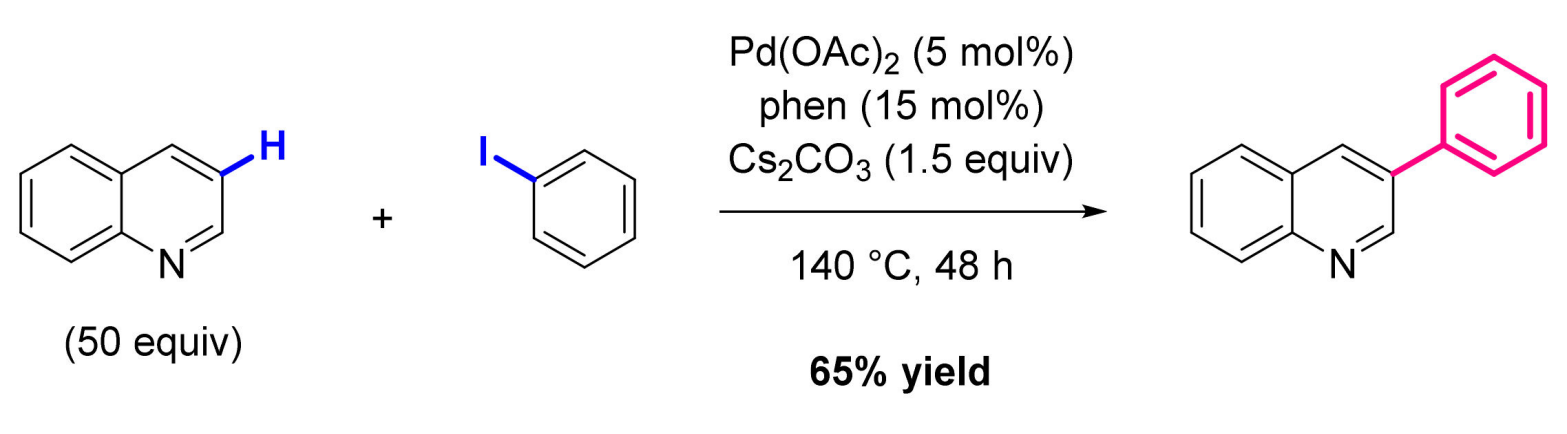
Pd-catalyzed L-promoted C3 arylation of quinoline with iodobenzene.

In the following years, the same team continued working on the C3-selective functionalization of pyridines with Pd catalysts. Both the C3 arylation and olefination were studied (see also the alkenylation paragraph) [146,147]. Optimization studies were carried out using pyridine as substrate and only the best conditions were applied to functionalize a quinoline: ligands such as bipyridines, substituted phenanthrolines or triphenylphosphines were ineffective; whereas 1,10-phenanthroline (phen) gave a 65% yield and, most importantly, the C3 selectivity was observed, even in the absence of the DG. Cs_2_CO_3_ revealed to be the best base and it was proved that the commonly used silver salts were not necessary for this reaction (Scheme 40). The main drawbacks of this coupling remained the necessity of a large excess of substrate and the lower selectivity observed for QN. In fact, because of the removal of the DG, 2-substituted QN was also isolated as minor product (C3/C2 3:1 ratio).

The authors proposed a mechanism similar to the *path a* described above in Scheme 3, relying on a standard OA/RE sequence. Investigation of KIE and competition reactions showed that the formation of the key aryl C3-Pd(II) intermediate **C** occurs through a CMD process (step **II**).

**Scheme 41:**
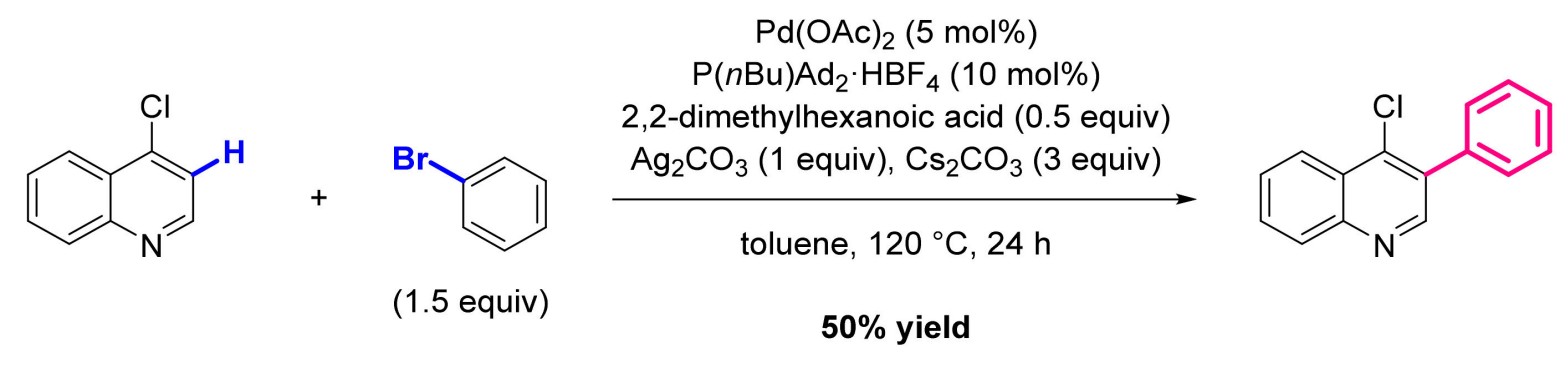
Pd-catalyzed L and LA-assisted C3 arylation of quinoline with bromobenzene.

Independently, the same year, Guo et al. reported a protocol allowing the intermolecular C-H arylation of mostly pyridines and some quinolines containing common EWGs (NO_2_, CN, F and Cl) [148]. In this case, the coupling is catalyzed by a Pd carboxylate catalytic system and it runs in the presence of a Lewis acid. Ag_2_CO_3_ is the most suitable among the tested silver salts to act as LA and halide scavenger, and Cs_2_CO_3_ was confirmed to be the best base. However, the phosphine ligand and the carboxylic acid need to be adapted to suit the substitution pattern previously present on the QN. The substrate-dependence of the reaction conditions is described more in details in the corresponding article (Scheme 41). The high regioselectivity is controlled by the electronic character of the C-H bonds and steric effects: the repulsion between the palladium carboxylate and the lone pair on the quinoline’s nitrogen prevents the functionalization in position 2, the LA enhances the reactivity of only the pyridine ring and, above all, the presence of the EWG determines the complete regioselectivity of the arylation either in position 3 or 4. More precisely, 3- and 5- substituted QNs undergo C4 arylation while 4-subsituted QNs undergo C3-arylation. The LA can also reduce the frequently described deactivating effect of the nitrogen on the catalyst.

**Scheme 42:**
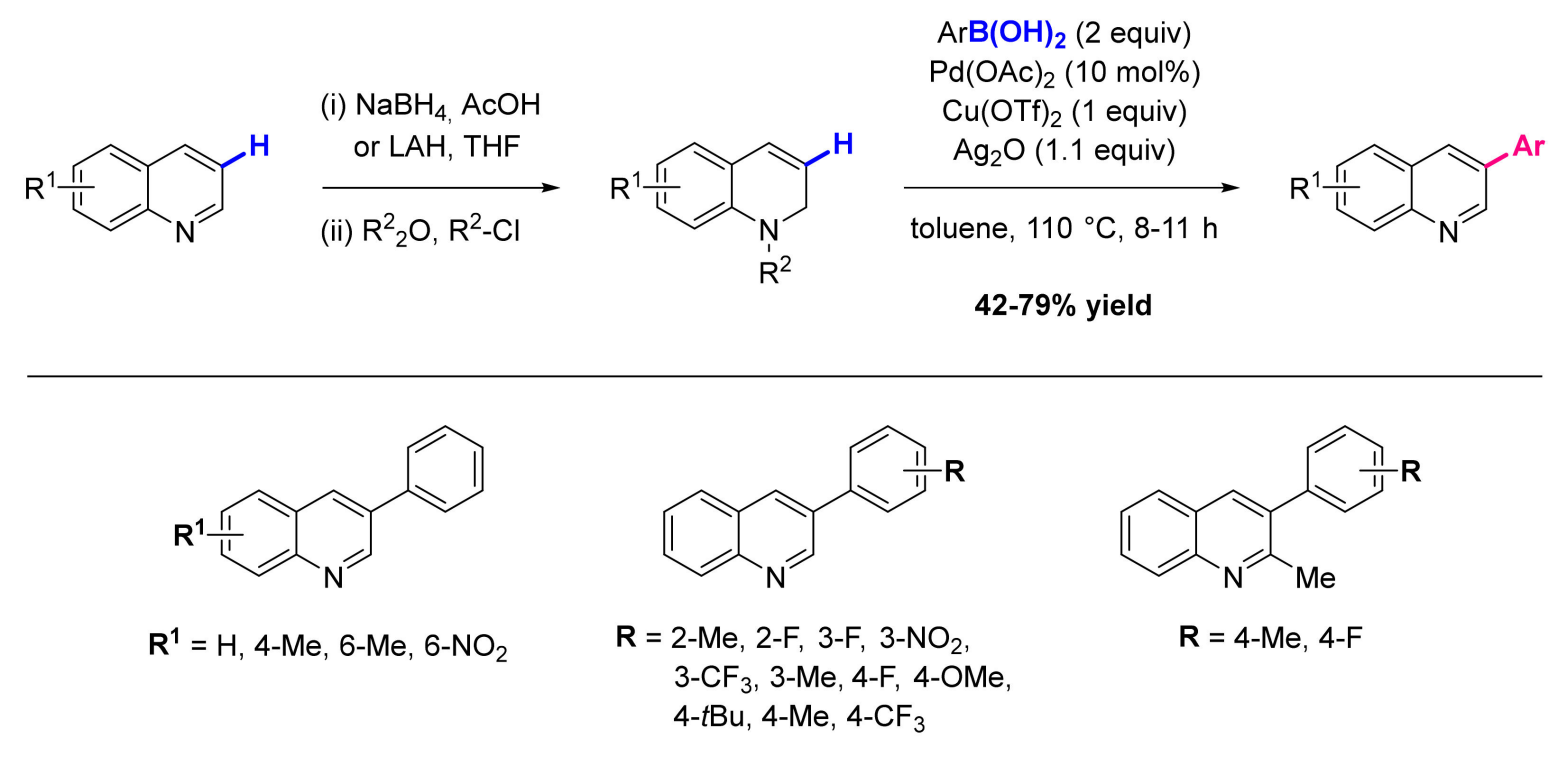
Pd-catalyzed C3 arylation of quinolines with arylboronic acids.

Another approach was explored by Kapur in 2013, who developed a regioselective Pd-catalyzed coupling between the C3-H of *N*-acyl-1,2-dihydroquinolines and arylboronic acids [149]. The method requires a pre-activation step if the starting compound is a quinoline, but the functionalization is achieved in good yield and regioselectivity. Moreover, it gives the 3-aryl quinoline through a one-pot sequence of Pd-coupling, *N*-deacylation and aromatization (Scheme 42). After the screening of numerous different catalytic systems and oxidants, the use of Pd(OAc)_2_ in the presence of Cu(OTf)_2_ and Ag_2_O gave the best results. The reaction has a broad scope: 14 examples with yields ranging from 57 and 79% are reported for QNs or arylboronic acids containing EDGs and neutral substituents. However, when the QN is substituted in position 2 or when EWGs are present, the yield decreases or the reaction doesn’t occur. The C3 selectivity is determined by the presence of the *N*-acetyl group, which, since it directs the reaction and is not found in the product, acts as a traceless directing group (TDG).

This reaction relies on the C-H activation of *N*-acetyl dihydroquinoline and thus does not correspond to the general mechanism depicted in Scheme 3. Therefore, the reader is invited to consult the original article for a detailed description if desired. 

**Scheme 43:**
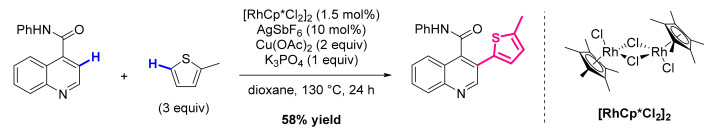
Rh-catalyzed DG-assisted C3 oxidative cross-coupling of quinoline with thiophene.

In 2014 the team of Su [150], as part of their studies on the Rh-catalyzed C-H/C-H cross coupling between pyridines and different heterocycles, reported the C3 functionalization of quinoline with a thiophene derivative, assisted by a *N*-phenylamide as NR-DG. The same reaction functionalizes the position 2 if the DG is located at C3. The reaction affords the 3-substituted quinoline in 58% yield using [Cp*RhCl_2_]_2_ as catalyst, AgSbF_6_, Cu(OAc)_2_ and K_3_PO_4_ in dioxane under N_2_ atmosphere (Scheme 43).

**Scheme 44:**
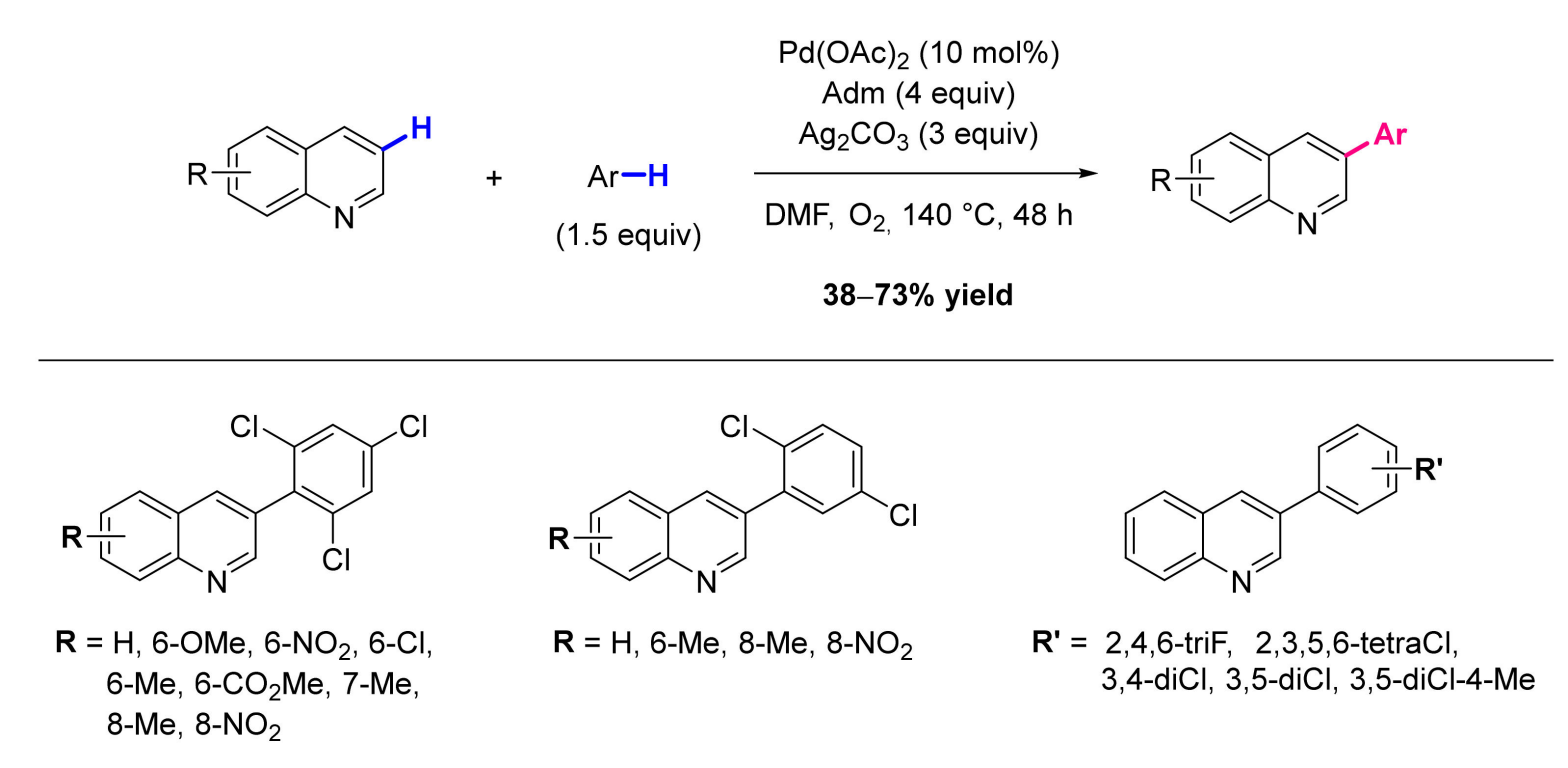
Pd-catalyzed L-promoted C3 oxidative cross-coupling of quinolines with arenes.

In 2016, the team of Huang, inspired from the results of Yu and from their studies on the C2 functionalization [112], developed a new method to synthesize C3-arylated quinoline derivatives through Pd(II) catalysis [151]. High reactivity and selectivity were achieved by using carboxylic acids as ligands and Ag_2_CO_3_ with O_2_ as oxidants (Scheme 44). Screening of solvents, oxidants and addition of phosphine did not improve the process, while the screening of ligands revealed that 1-ada-mantanecarboxylic acid (Adm), compared to the other ligands explored, could increase the yield as well as decrease the reaction time. The scope of the reaction was explored by adding substituents on the quinoline scaffold. Most of the common substituents (OMe, NO_2_, halogens, esters…), both EWGs and EDGs, were well tolerated and afforded selectively the desired 3-aryl quinolines in moderate to good yields (mostly from 58 to 73%, less when a methyl group was present in position 2 or 4). Different mono-chlorobenzenes were also introduced in good yields, even though with a lower selectivity in a few examples, namely when the monochlorobenzene was used as coupling partner, it led to a mixture of *meta*- and *para*-chloro derivatives.

The mechanism proposed by the authors involves a double C-H activation process that matches with the *path a* described above in the Scheme 3, for which the carboxylate ligand plays the role of the base for the deprotonation step **II,** and the silver salt is necessary to reoxidize the Pd (0) into Pd(II) after the RE step **IV**.

### 3.2. Formation of C-Alkenyl Bonds

**Scheme 45:**
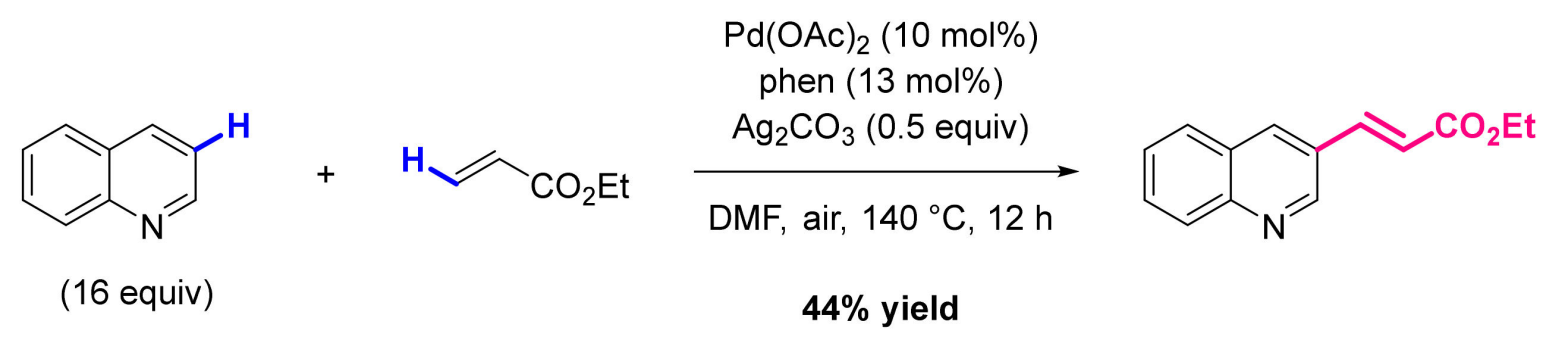
Pd-catalyzed L-promoted C3 alkenylation of quinoline with ethyl acrylate.

In 2011, in parallel with the already described C3 arylation, the team of Yu reported a Pd-catalyzed and ligand-promoted C-H olefination selective for the position 3 of pyridines and quinolines [147]. Optimization of the reaction, involving the screening of solvents, ligands and Pd sources, was carried out on a pyridine substrate and the best conditions were applied to the unsubstituted quinoline, leading to the desired product in 44% yield (Scheme 45). The authors attribute the selectivity of the reaction to the bidentate ligand, ie 1,10-phenanthroline (phen), which is responsible for the assembly of the reactive C-Pd-H intermediate through the trans-effect.

**Scheme 46:**
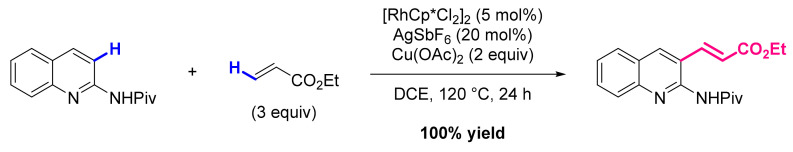
Rh-catalyzed DG-assisted C3 alkenylation of quinoline with ethyl acrylate.

The same reaction was performed by Shi and co-workers in 2013 with Rh catalysis using an amide as DG [152]. After being optimized on pyridines, it was then reported that *N*-(quinolin-2-yl)pivalamide reacts with ethyl acrylate in the presence of [Cp*RhCl_2_]_2_ (5 mol%), AgSbF_6_ (20 mol%), Cu(OAc)_2_ (2.0 equiv.) in DCE for 24 h to give the desired functionalized quinoline with quantitative yield (Scheme 46). It is important to note that this reaction is also one of the rare examples of successful C7 regioselective functionalization. The pivalamide acts as directing group: when it is in position 2, the olefination is regioselective for the C3, while when it is in position 8, the less reactive position 7 is substituted.

### 3.3. Formation of Other C-C Bonds

**Scheme 47:**
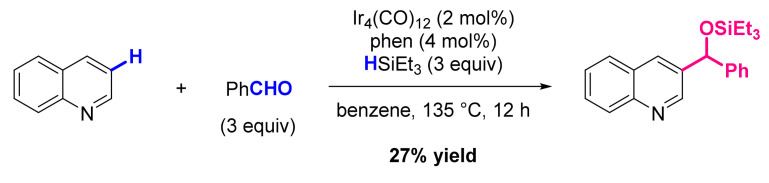
Ir-catalyzed C3 alkylation of quinoline with benzaldehyde and triethylsilane.

In 2010, the team of Shi reported the first *meta* selective Iridium-catalyzed C-H activation of pyridine derivatives and their consequent addition to aldehydes promoted by triethylsilane (Scheme 47) [153]. The reaction was optimized on a phenylpyridine substrate by screening different metal catalysts, like Mn, Re, Ru, Rh or Ir, different Iridium ligands, solvents and silanes. Only the use of Ir_4_(CO)_12_ catalyst allowed the reaction to occur with satisfactory yields and the best conditions involved the use of 1,10-phenanthroline (phen) and triethylsilane in benzene at 135 °C for 12 h. The scope of 11 pyridine derivatives also included the unsubstituted quinoline, which afforded selectively the 3-substitued product in 27% yield.

After intensive experimental investigations, the authors proposed a mechanism that is similar to the *path b* described above in the Scheme 3, starting with the formation of an active silyl iridium catalyst **C′**, generated from the combination of Ir precursor and silane, followed by the OA of the pyridyl C-H bond to produce the key intermediate **D**. From there, benzaldehyde insertion into the Ir-Si bond and subsequent RE (step **IV)** yield the product and an iridium hydride species, which is finally reoxidized by hydrosilane to close the catalytic cycle.

**Scheme 48:**
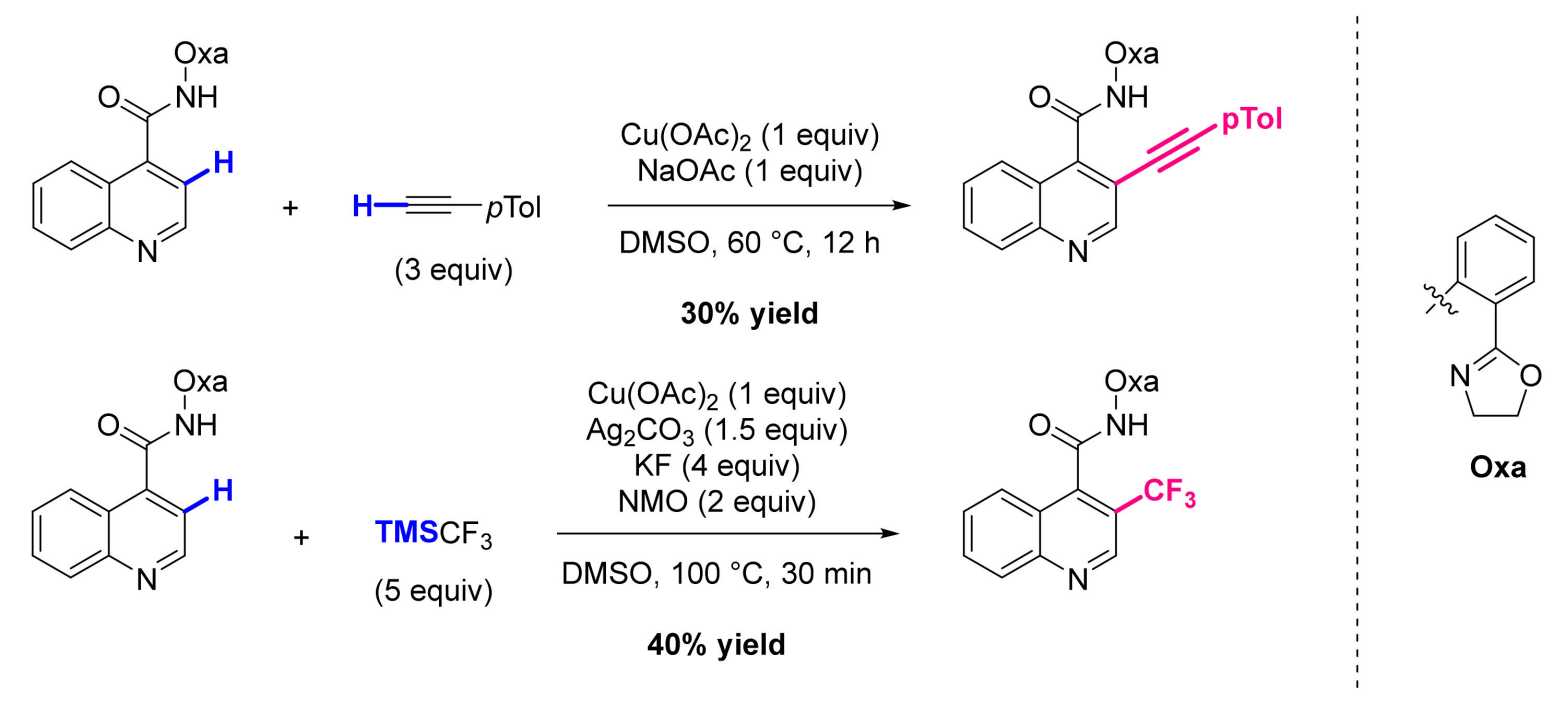
Cu-catalyzed DG-assisted C3 functionalization of quinoline.

In 2014, Shang et al. published two similar Cu-promoted procedures to functionalize, with the aid of a directing group, arenes and heteroarenes [154,155]. Among them, quinoline was selectively functionalized in C3 with alkynes or with a trifluoromethyl substituent in 30% and 40% yield, respectively. The DG was again an amide, namely the carbo-amide of the 2-(2-oxazolyl)aniline (Scheme 48).

The mechanism proposed by the authors corresponds to the *path a* described above in the Scheme 3, starting with the oxazoline-directed formation of a C3-Cu(II) complex which disproportionates to yield the corresponding organocopper(III) complex C, followed by transmetalation with TMSCF_3_ and RE (steps **III** and **IV**) to furnish the product and regenerate the catalytic species. Experimental investigations carried out with TEMPO and measurement of intra- and intermolecular KIE supported a copper-mediated C-H cleavage (step **II**) rather than an electrophilic aromatic substitution (SEAr) or a radical pathway.

### 3.4. Formation of C-Het Bonds

**Scheme 49:**
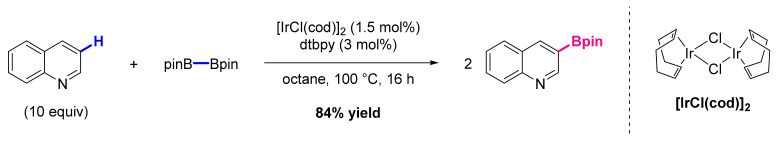
Ir-catalyzed C3 borylation of quinoline with bis(pinacolato)diboron.

C3-H activation has also been used to synthesize important building blocks for further use in synthesis, such as borylated or silylated compounds. The first C3-H functionalization of quinolines to form C-Het bonds dates back to 2002, when Ishiyama, Miyaura and co-workers, as part of their extensive studies on the Ir-catalyzed synthesis of heteroarylboronates, reported the borylation of a not-functionalized quinoline catalyzed by [IrCl(cod)]_2_ and 4,4′-di-*t*-butyl-2,2′-bipyridine (dtbpy) in octane with 84% yield (Scheme 49) [156].

The team of Steel further explored the Ir-catalyzed borylation of aromatic C-H bonds and published their findings in two articles in 2009 and then in 2012 [157,158]. In the first one, a microwave-accelerated procedure with several arenes and heteroarenes as substrates is described: the reaction has in general very good yields and selectivity, while reaction times are highly shorter compared to the standard heating procedures. However, the use of quinoline as substrate leads to a mixture of regioisomers with a double functionalization in position 3,6 and 3,7. In the second one, an extensive study on the effect of steric and electronic factors on the regioselectivity of the C-H borylation on quinolones is reported: 26 different quinolines, monosubstituted and disubstituted, were used as substrates and reacted with one or two equivalent of B_2_pin_2_ to study both the mono- and the di-borylation. Results and methods highly depend on the substitution pattern of the QN. Therefore, as no general trend can be highlighted, the reader is invited to consult the original article for more information if desired.

**Scheme 50:**
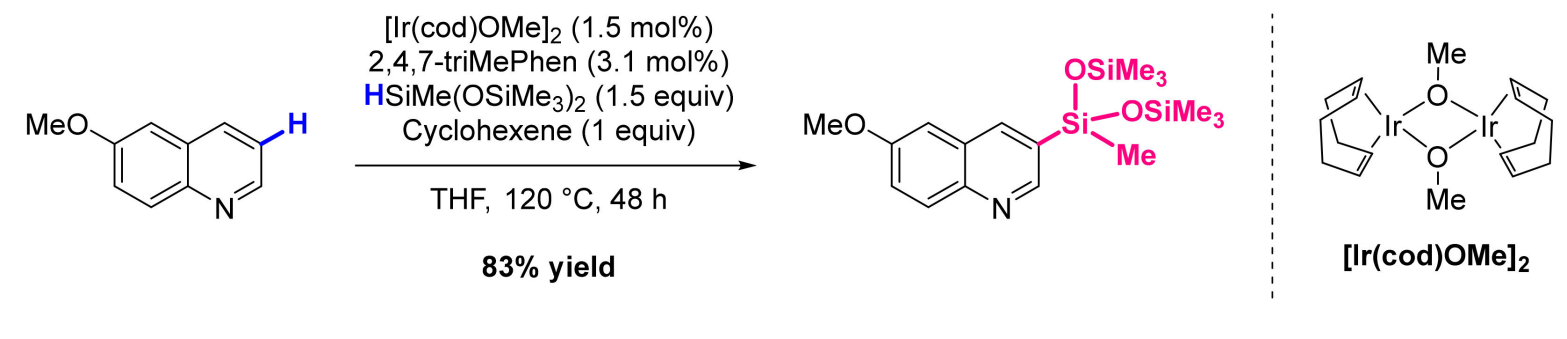
Ir-catalyzed C3 silylation of quinoline with silane.

In 2015, Chang, Hartwig and collaborators, still working on different arenes and heteroarenes, focused their attention on a new Ir-catalyzed C-H silylation [159]. The reaction was initially developed by the group with a Rh-catalyst [160], but its unsuitability for nitrogen-containing substrates and also the long experience of the team with the Ir-catalyzed borylation of heteroarenes prompted them to explore the Ir-catalytic system. Successfully, the reaction of 6-methoxyquinoline and HSiMe(OSiMe_3_)_2_, catalyzed by [Ir(cod)OMe]_2_ in the presence of 2,4,7-trimethylphenanthroline (2,4,7-triMePhen) in THF at 100 °C afforded selectively the 3-silylated-6-methoxyquinoline with a 83% yield (Scheme 50).

Mechanistic aspects were previously discussed by Hartwigh’s team in 2005 [161] and later in 2013 [162].

## 4. Functionalization in Position 4

Examples of CH-functionalization in position 4 are very scarce. The few existing methods allow the introduction of alkyl, alkenyl and aryl moieties. Apart from two Pd-catalyzed reactions, in most cases the C4-H activation relies on a Ni-Al catalysis in which Al acts as a LA while Ni is the true catalyst.

**Scheme 51:**
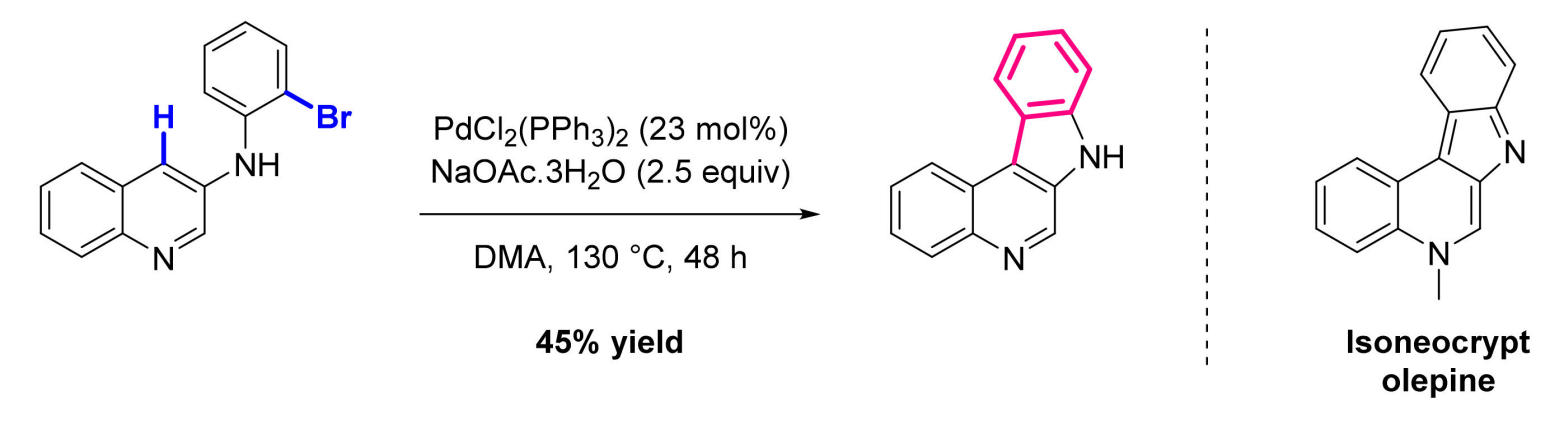
Intramolecular Pd-catalyzed C4 arylation of quinoline.

The first example is the work of Maes in 2005, which applied an intramolecular Pd-catalyzed C4-H activation towards the synthesis of a natural compound, the isoneocryptoleine [163]. The desired product was obtained by intramolecular C-H functionalization with PdCl_2_(PPh_3_)_2_ as catalyst and NaOAc as base, in DMA at 130 °C in a 45% yield, while the 2-substituted isomer being isolated in only 4% yield (Scheme 51).

**Scheme 52:**
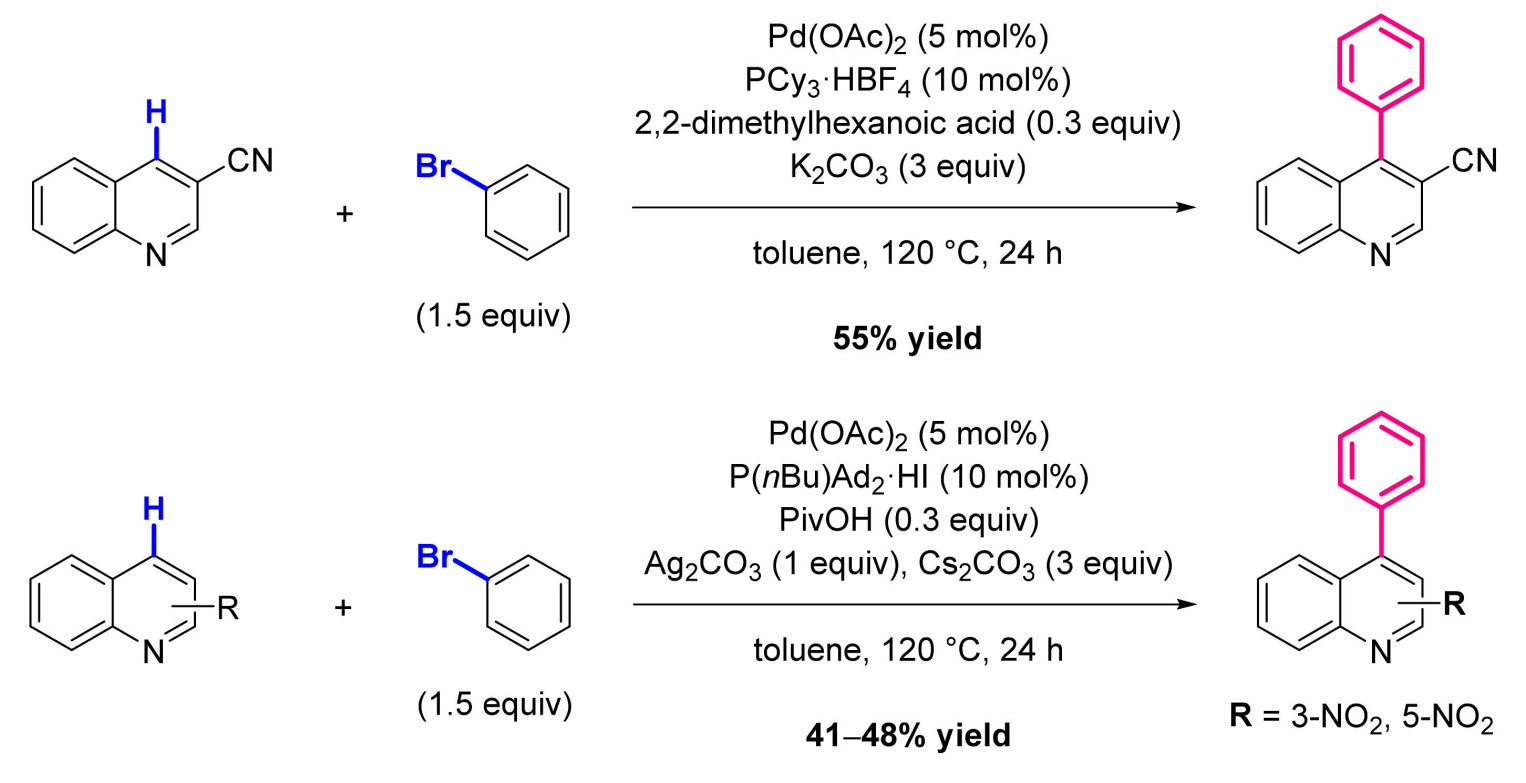
Pd-catalyzed DG-assisted C4 arylation of quinolines with phenyl bromide.

As previously mentioned in Section 3, Guo et al. described in 2011 the Pd-catalyzed C-H arylation of pyridines and quinolines directed in positions 3 and 4 with the aid of an EWG substituent acting as NR-DG [148]. Starting from cyano and nitro derivatives, the C4 arylation of quinoline with phenyl bromide afforded the desired products with moderate 41 to 55% yields (Scheme 52).

**Scheme 53:**
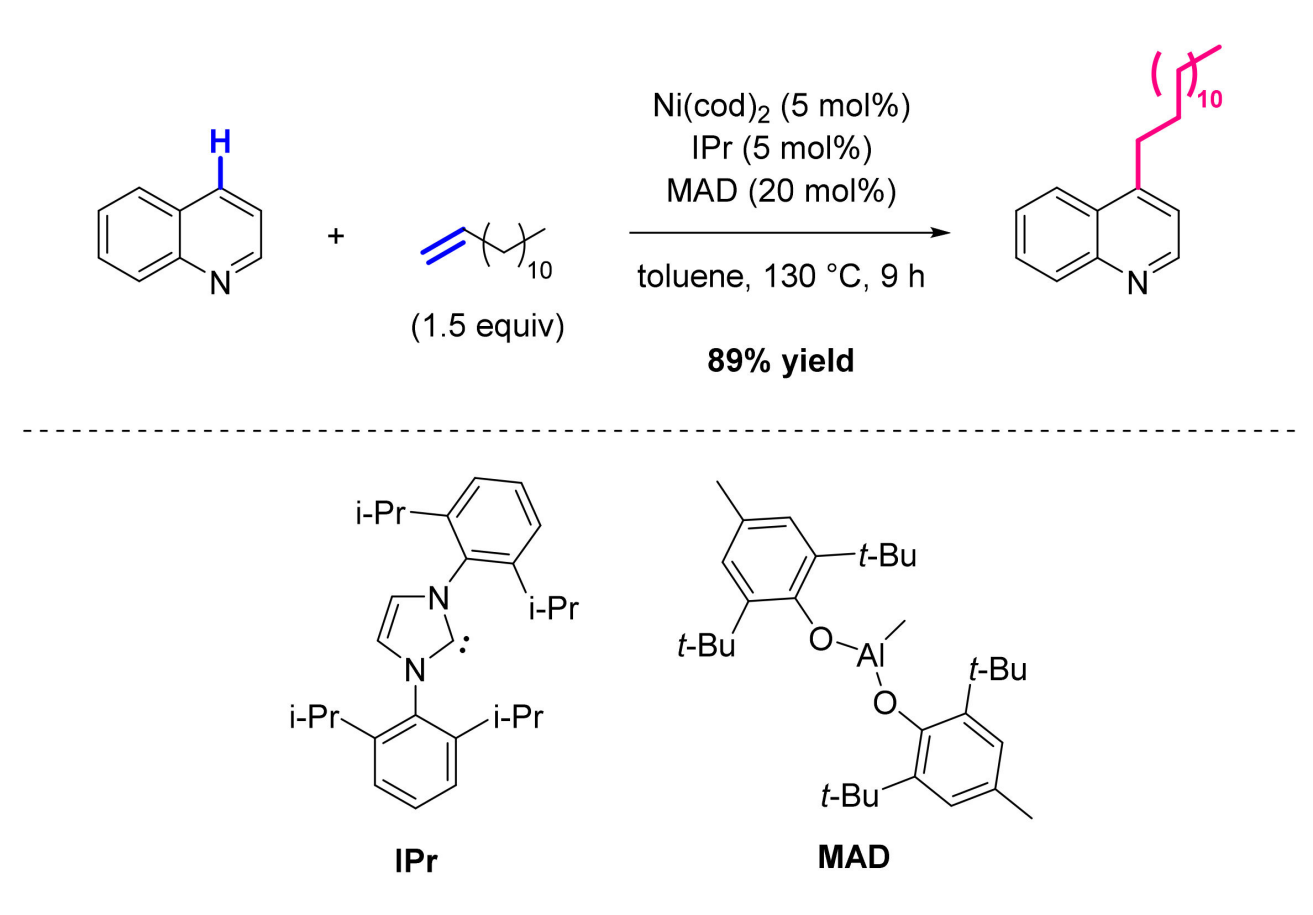
Ni-catalyzed LA-assisted C4 alkylation of quinoline with 1-tridecene.

In 2010 Hiyama’s group published the first selective C4 alkylation of pyridine with alkenes by Nickel/Lewis Acid catalysis [124]. This team, who previously worked on the C2 alkylation of pyridines and quinolines, found that nickel catalyst was very useful to functionalize these heterocycles because the Ni-complex could bind the nitrogen and catalyze the C-H activation selectively in position 2. They proposed that it could be possible to re-direct the functionalization selectively in position 4 by adding an Al-complex which could interact with the heteroatom instead of Ni and by using a highly bulky *N*-heterocyclic carbene (NHC) ligand which would prevent the C3 functionalization by steric hindrance. Applying this strategy, they found that the reaction of quinoline (1.0 mmol) and 1-tridecene (1.5 mmol) in the presence of Ni(cod)_2_ (5 mol%), 1,3-(2,6-diisopropylphenyl)imidazol-2-ylidene (IPr, 5 mol%), and MAD (20 mol%) as LA catalyst in toluene at 130 °C for 3 h gave 4-tridecylquinoline in 89% yield (Scheme 53) [164].

After experimental studies carried out with deuterated substrates, the authors proposed a mechanism which corresponds to the *path a* described above in the Scheme 3, involving an alkene insertion/RE sequence. From their results, it appeared that both sterically highly demanding Lewis acid and NHC ligand are crucial to induce the C4 vs. C2 and C3 selectivity on the C-H activation step **II** as well as the RE step **IV**.

**Scheme 54:**
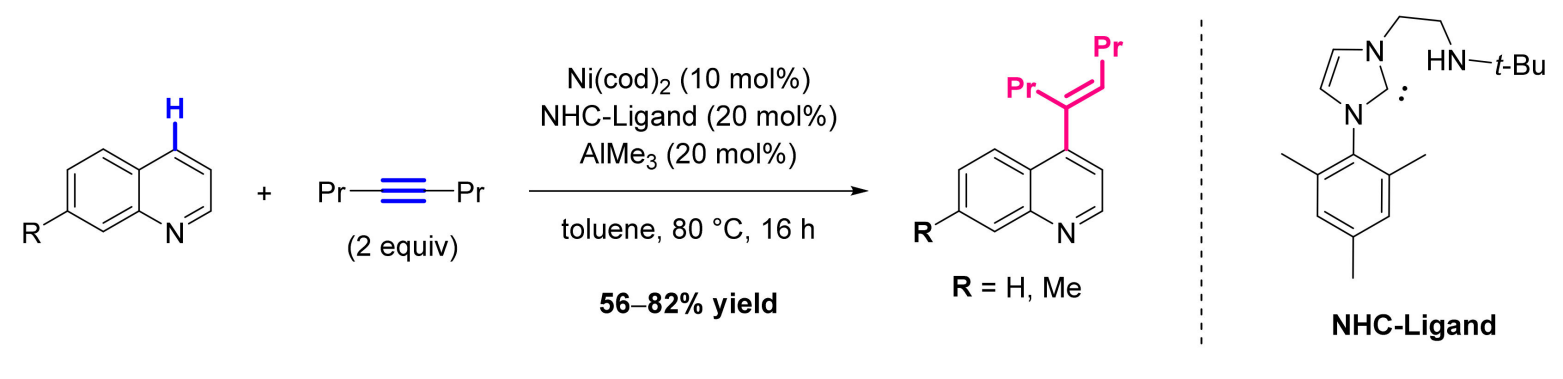
Bimetallic Ni/Al-catalyzed LA-assisted C4 alkenylation of quinolines with 4-octyne.

The same year, Ong’s group reported the bimetallic catalysis with Ni-AlMe_3_ system for the C4 alkenylation on pyridines and quinolines [165]. Their work is highly complementary to the one of the Hiyama group, allowing the coupling of quinolines with alkynes to provide C4-alkenylated products in 56–82 % yields (Scheme 54). Optimization studies were carried out with a pyridine substrate and included both ligands and Lewis acids screenings. This catalytic method features a cooperative interaction between Ni and Al to enable the remote para C-H activation.

After intensive experimental investigation, the authors proposed a mechanism which corresponds to the *path a* described above in the Scheme 3, relying on an alkyne insertion/RE sequence. The structure of a bimetallic η2,η1-pyridine Ni(0)-Al(III) complex that is supposed to be the intermediate between **B** and **C** was unambiguously confirmed by X-ray crystallography. This complex proved to be catalytically active and further measurement of KIE demonstrated that the C-H bond breaking (step **II**) is not the rate-limiting step of the reaction.

**Scheme 55:**
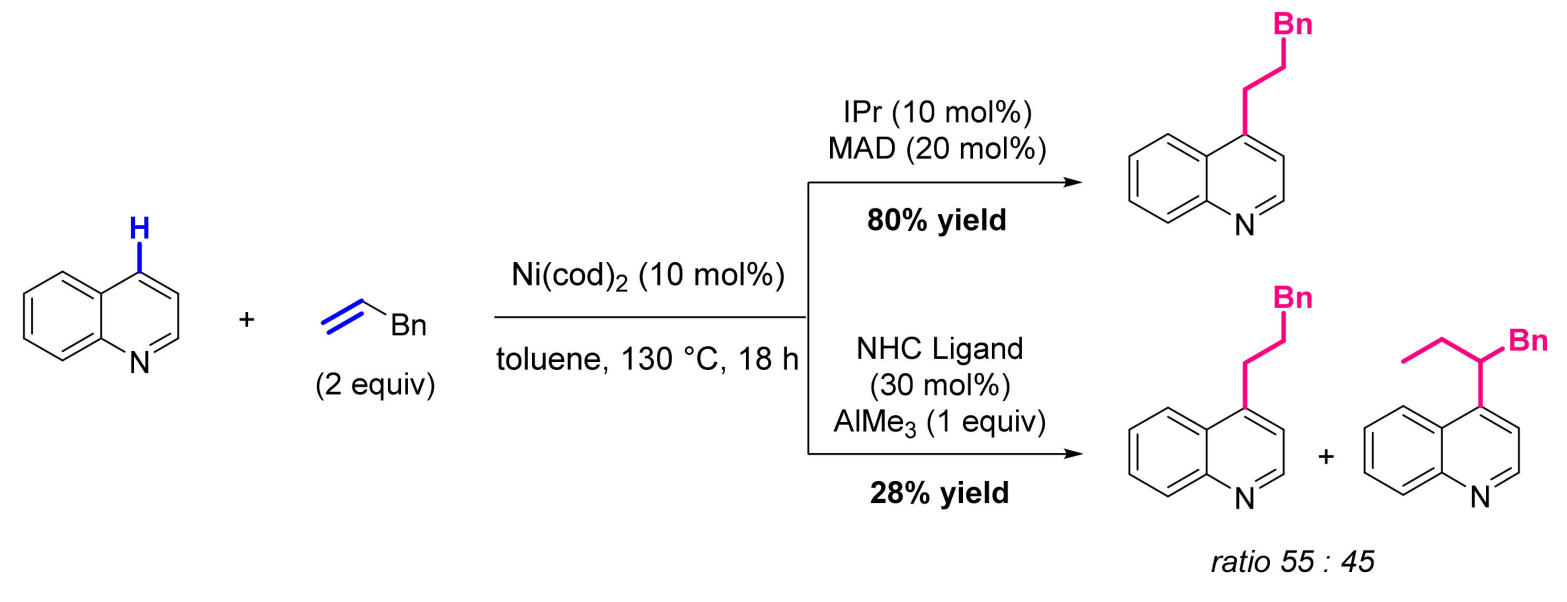
Bimetallic Ni/Al-catalyzed LA-assisted C4 alkylation of quinoline with styrene.

Ong’s team further studied, in the following years, the Ni-Al catalyzed C4-alkylation of pyridines and quinolines with alkenes by developing a method to control the isomerization of the formed product, and thus to obtain selectively linear or branched C4-alkylated compounds (Scheme 55) [166]. As already described by Hiyama, a complete regioselectivity was reported for the linear isomer in high yields (95% for pyridines and 80% for quinoline) with IPr as ligand and MAD as additive. Then, the steric congestion around the metal center was reduced using a tertiary amino-NHC as ligand and AlMe_3_ as additive. As before, this strategy was optimized and gave fine results with pyridines, affording the branched product in yields around 80% and selectivity 99:1. However, the results with a quinoline substrate were less encouraging, since the yield dropped at 28% and it was not selective (branched and linear isomers were found in a 55:45 ratio).

After several control reactions in order to investigate the mechanism, the authors proposed a possible reaction pathway consisting of two operating catalytic cycles: the first one leading to the Ni-catalyzed isomerization of the alkene, which was evidenced by GC and NMR, and the second one resulting in the C-H functionalization, according to the *path a* described above in the Scheme 3.

**Scheme 56:**
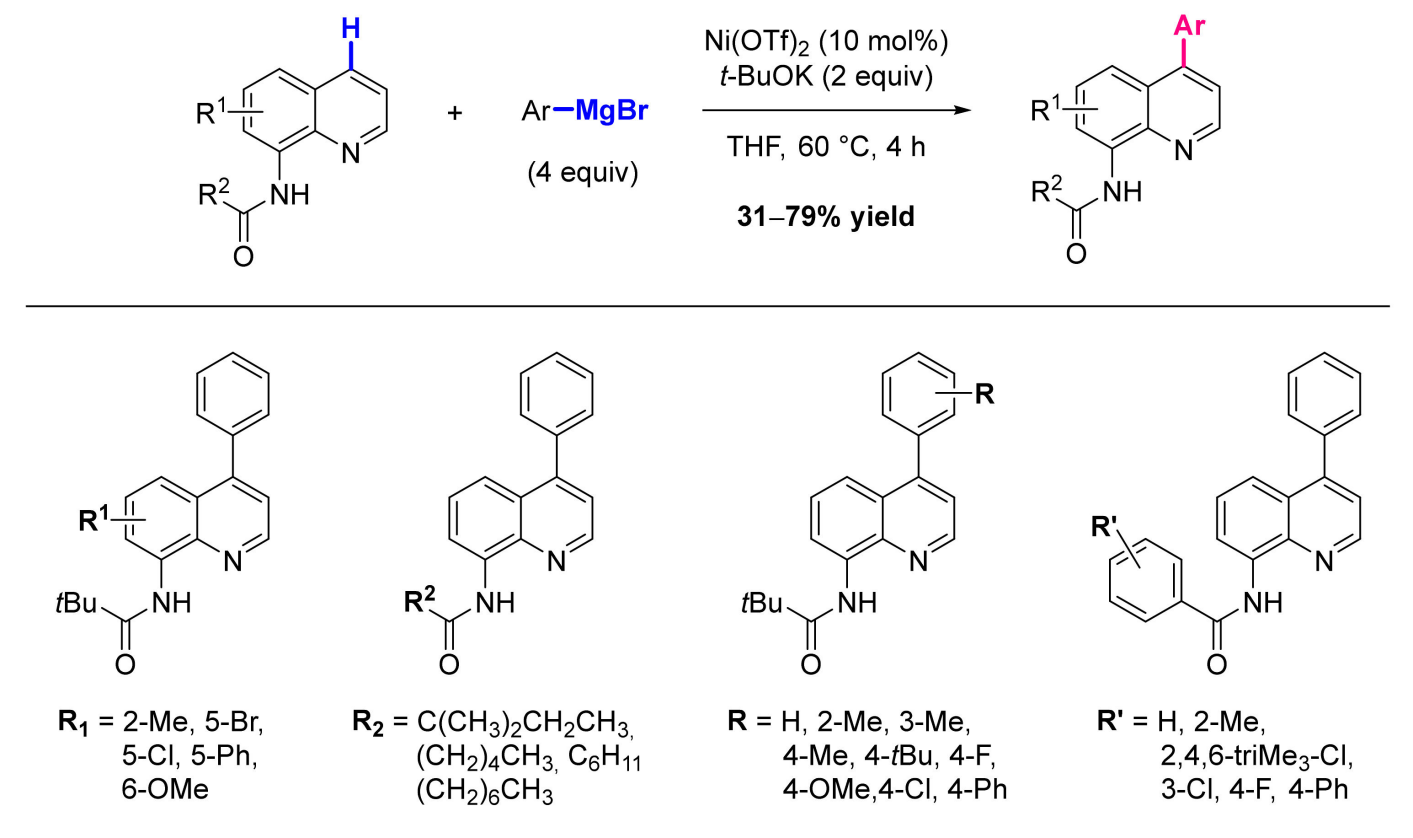
Ni-catalyzed C4 arylation of quinolines with aryl magnesiumbromides.

Very recently, in 2019, the team of Qiu and Kambe reported their work on the nickel-catalyzed remote C4-H arylation of 8-aminoquinolines, an important class of molecules which is widely represented in biologically active compounds, materials and DG or ligands for metal catalysis (Scheme 56) [167]. Several reactions have been developed for their C5 functionalization but, to our knowledge, this is the only example where a metal catalyzed C4-H activation pathway is described. During the optimization studies, different reaction conditions were tested regarding the Ni catalysts, the equivalents of the reagents or the presence of the base. Reaction of *N*-(phenylquinolin-8-yl)pivalamide with 9 different aryl Grignard reagents, in the presence of Ni(OTf)_2_, and *t*-BuOK at 60 °C gave the C4 arylated products with yields ranging from moderate to good (60–70% when introducing aryls with EDGs and 40–50% when EWGs). Similar results were obtained when the arylation was performed with PhMgBr on 14 different 8-aminoquinolines (both different 8-amidogroups or different substituents on the QN moiety).

After extensive experimental investigation, two mechanisms were proposed by the authors: in the first one, Ni acts as a Lewis acid that promotes the addition of the Grignard reagent, whereas the second one is a Ni-catalyzed C-H activation process that matches with the *path a* described above in the Scheme 3. In this latter case, deprotonation of the amide by *t*BuOK occurs prior to the C-H activation (step **II**), and is followed by a standard transmetalation/RE sequence.

## 5. Functionalization in Position 5

Metal-catalyzed remote C-H functionalization in position 5 of quinolines has been widely explored in the last years [99], especially with 8-aminoquinolines as substrate, but only three examples are relying on a true C-H activation mechanism. 

**Scheme 57:**
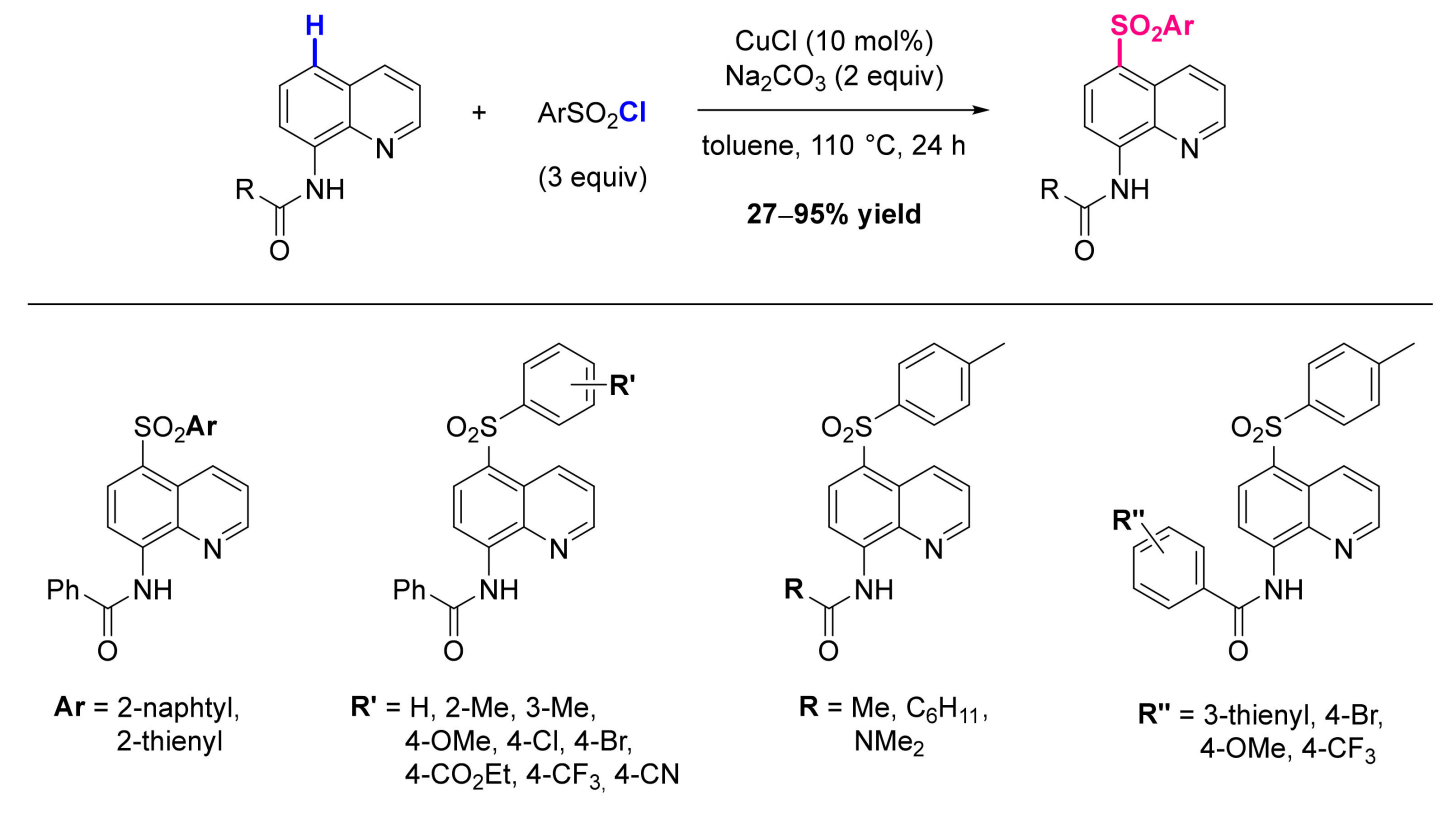
Cu-catalyzed AG-assisted C5 sulfonylation of quinolines with arylsulfonyl chlorides.

The first report of C5-H functionalization though C-H direct activation is the Cu-catalyzed sulfonylation discovered by Wei et al., [168]. Using 8-aminoquinolines and aryl sulfonyl chlorides, the C5 sulfonylated quinolines were obtained regioselectively in good to excellent yields. Among different copper salts and other metal catalysts (Pd, Rh, Fe, Au, Ni, Zn), CuCl gave best result. The screening of different solvents, bases and catalytic charges revealed the use of 10 mol% of CuCl and 2 equivalents of Na_2_CO_3_ in toluene at 110 °C as the best conditions (Scheme 57). With these optimized reaction conditions, the substrate scope was explored with a pannel of different sulfonyl chlorides and aminoquinoline amides (respectively 12 and 6 examples). All the different aromatic (aryl or heteroaryl) sulfonyl chlorides exhibited good reactivity, with reaction yields ranging from 65 to 95%. Only the presence of cyano-substituents or alkyl sulfonyl chloride was not well tolerated during the reaction. It is worth to cite that in the case of 5-OMe substituted 8-aminoquinolines, the sulfonylation reaction takes place at the disfavored 7-position.

After experimental studies, the authors proposed a mechanism which corresponds to the *path b* described above in the Scheme 3, starting with the chelation of CuCl with the DG and the QN ring, that may enhance the polarization of the C5-H bond, followed by the OA of the arylsulfonyl chloride to CuCl to furnish the complex **C′**. These two species then react together to form the key Cu(III) intermediate **D** which in turn undergoes RE (step **IV)** to yield the product and to regenerate the catalyst.

**Scheme 58:**
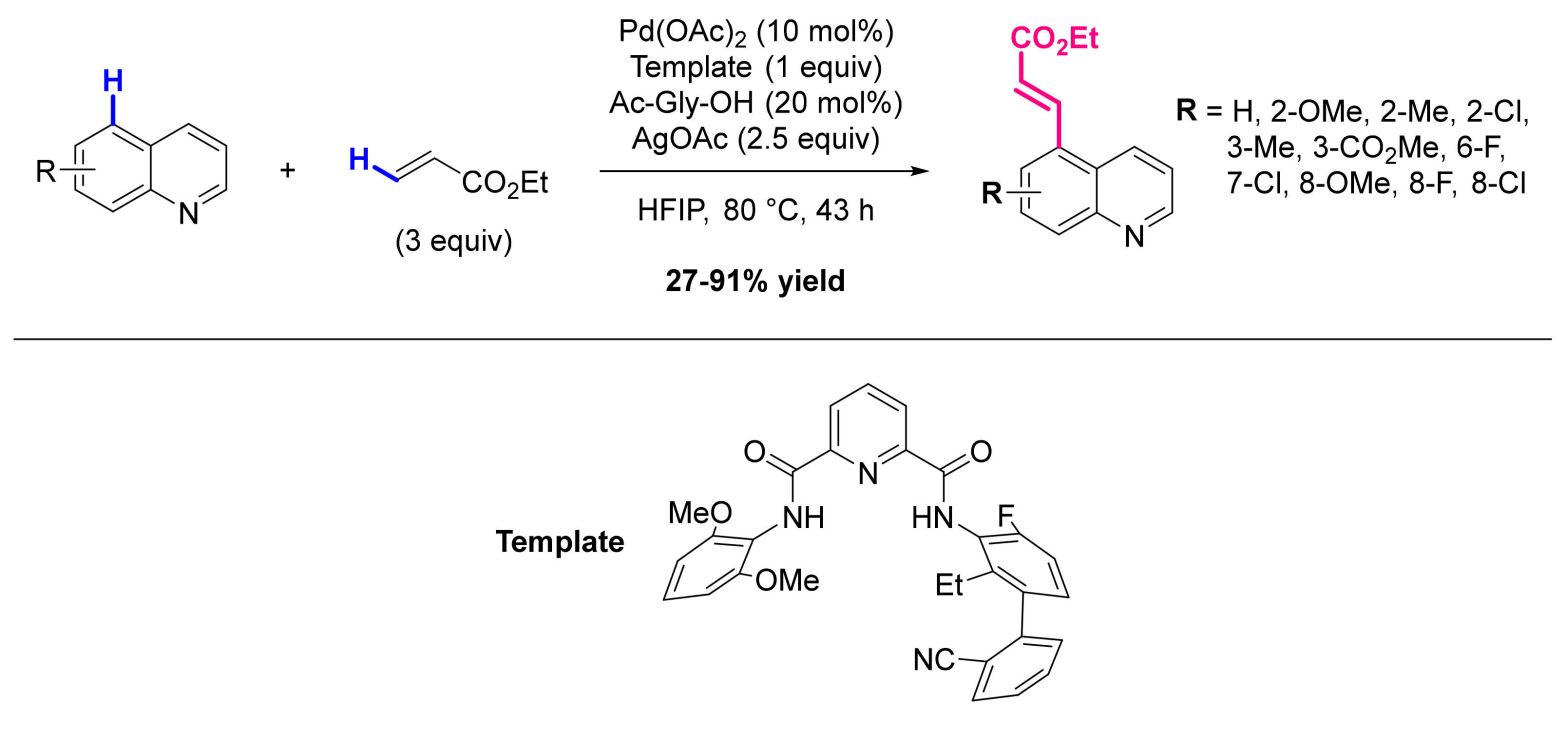
Pd-catalyzed TDG-assisted C5 alkenylation of quinolines with ethyl acrylate.

In 2017, Zhang, Tanaka and Yu succeeded in functionalizing quinolines in C5 without the assistance of the 8-amido directing group, developing a palladium-catalyzed olefination reaction directed through non-covalent interactions [169]. They used a bi-functional nitrile template that binds the substrate via reversible coordination. This template includes two metal centers: the first one is able to chelate the nitrogen of the QN substrate and to anchor it to the catalyst, while the second one directs the palladium catalyst towards the desired C-H bond. The bifunctional ligand consists in a central pyridine functionalized with two amido-functions. Once complexed in acetonitrile, they form a fourth coordination site for the first Pd center. The second metal coordination site is formed through the distal cyano-groups. Optimization of the reaction consisted in the choice of the template between 8 synthetized nitrile templates, as well as the screening of catalysts and ligands: the reaction of quinoline with ethyl acrylate in the presence of the optimized complex T18, a catalytic amount of Pd(OAc)_2_ and *N*-acetyl-protected glycine, gave the desired product in 75% yield. Furthermore, it was proved that the template complex can be recovered in 96% yield through a simple protocol, thus demonstrating that it is a recyclable reagent. The reaction proved to be tolerant since a wide range of QNs, containing both EWGs and EDGs were converted in good yields and selectivity (Scheme 58).

**Scheme 59:**
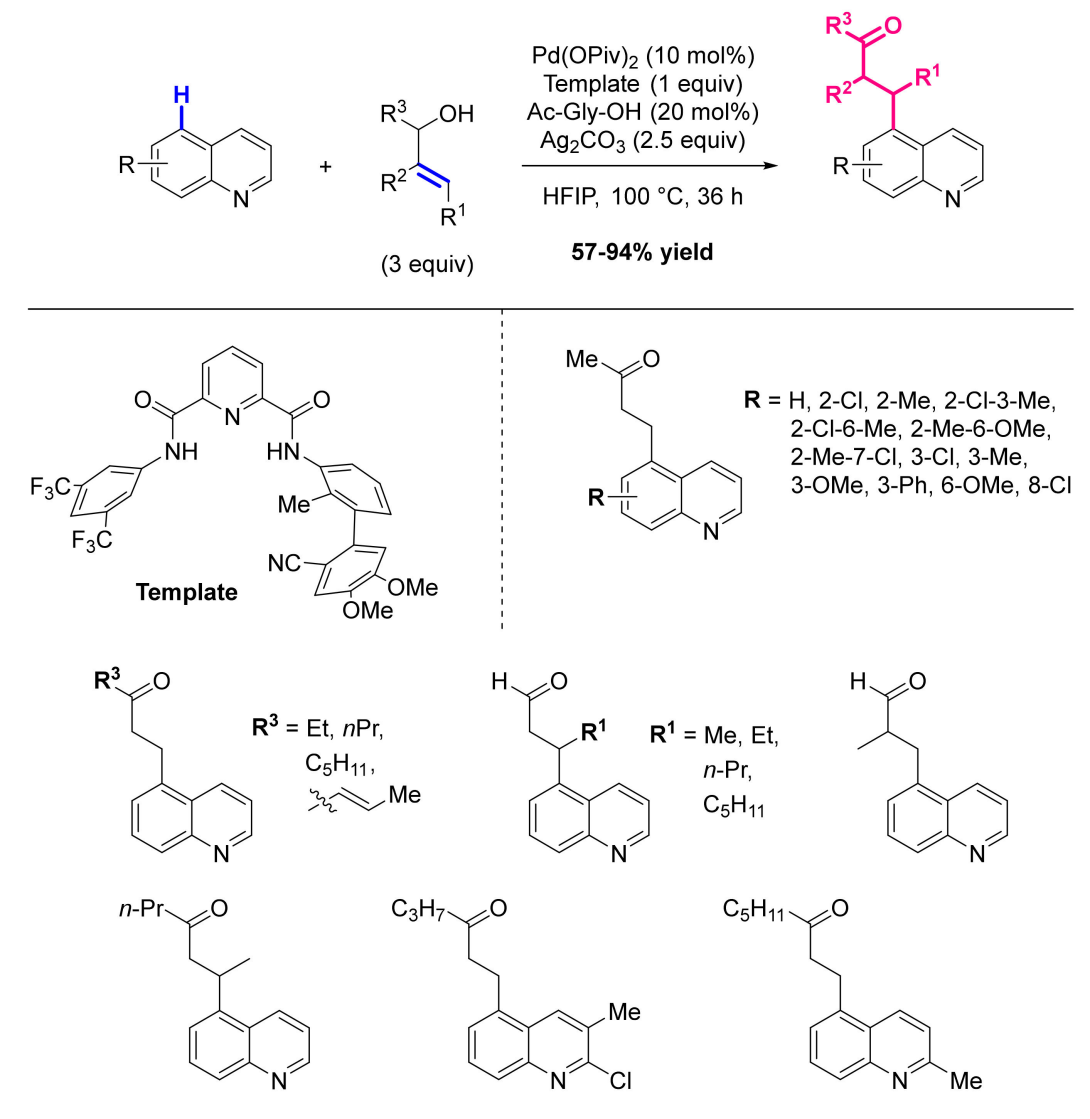
Pd-catalyzed TDG-assisted C5 alkylation of quinolines with allyl alcohols.

In 2019, inspired by the work of Yu and coworkers on the olefination of quinolines, and in the continuation of their recent work on template systems and C-H activation of quinolines, benzoxazoles, benzothiazoles and thiazoles [170], the team of Maiti further optimized this strategy and broaden the scope of possible coupling partners. The template was optimized and distal alkylation of nitrogen containing heterocycles with allylic alcohols was achieved, obtaining saturated ketones and aldehydes [171]. Both the efficiency and the selectivity of the C5 alkylation were increased thanks to the screening of 19 different unsymmetrical pyridine-bisamide templates synthetized by the group. The process was further optimized regarding the ligand, the solvent and the oxidant. The scope of the reaction includes various substituted quinolines, bearing EDGs and EWGs, and various allyl alcohols, leading to the desired coupling products in moderate to excellent yields and selectivity (Scheme 59).

The authors proposed a mechanism that is consistent with the *path a* described above in the Scheme 3, which was validated through the characterization of several intermediates by X-ray crystallography and other spectroscopic techniques. It starts with the template-assisted C-H activation (step **II**), followed by the migratory alkene insertion to provide an enol as key intermediate **D′**, which evolves into the corresponding ketone through tautomerization (step **IV**). Finally, the addition of DMAP allows the release of the alkylated product from the template complex and an acidic treatment in acetonitrile regenerates in each cycle more than 90% of the template.

## 6. Functionalization in Position 6

**Scheme 60:**
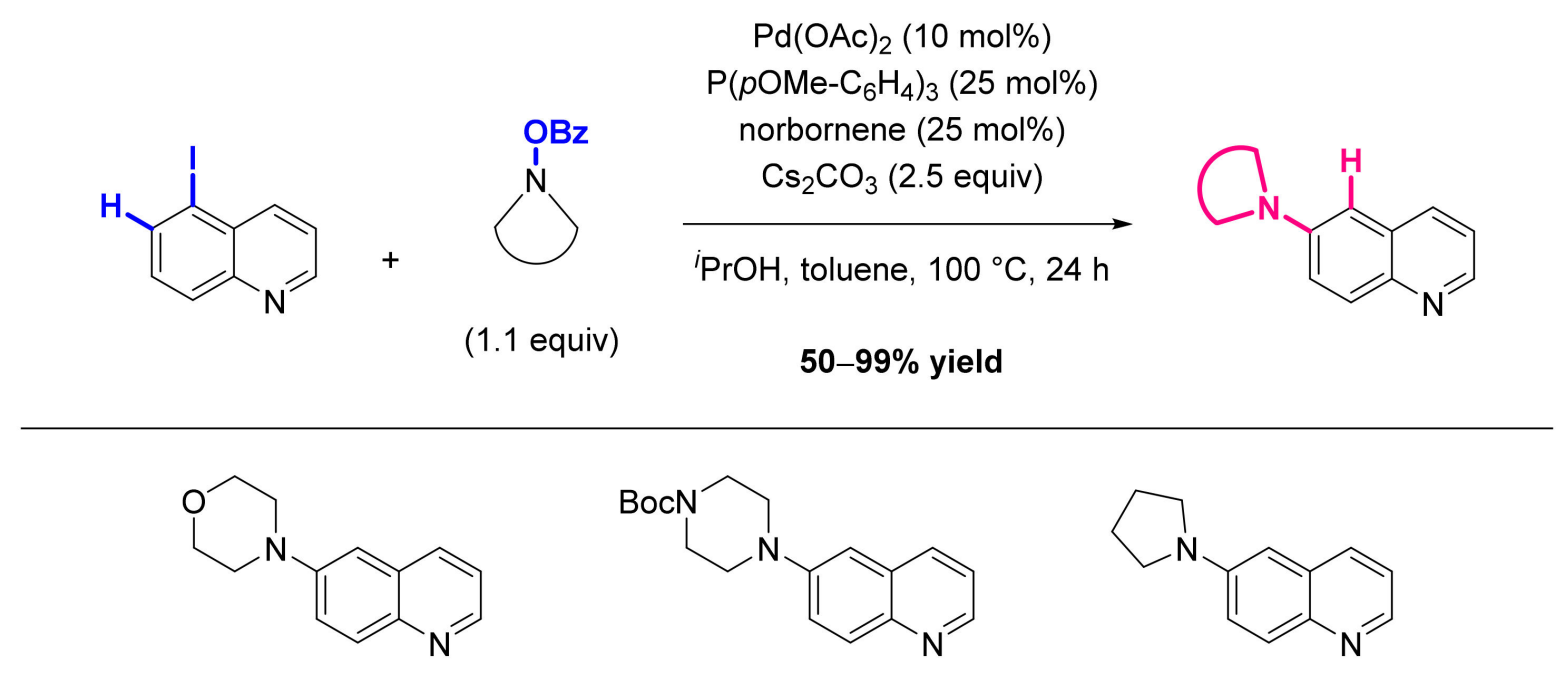
Pd-catalyzed C6 amination of 5-iodoquinoline with *N*-benzoyloxyamines.

As far as we know, there is no example, and even less full scope study, of any metal-catalyzed method to afford the C6-H functionalization of quinolines, or quinoline *N*-oxides, described in the literature. Usually, C6-functionalized QNs are reported as side products, which was for instance the case during the investigation of C8-H borylation discussed below in this review [172]. Nevertheless, C6-amination of QNs can be achieved starting from the 5-iodoquinoline thanks to the Pd- and norbornene-catalyzed Catellani reaction, as reported by the team of Dong in 2013 [173]. Primarily optimized using iodotoluene as substrate, this reaction proved to tolerate also iodoquinoline and furnished three aminated products in 50–99% yields (Scheme 60).

The Catellani mechanism is much more complex than the general mechanism depicted above in Scheme 3, therefore the reader is invited to consult the original article for a detailed description if desired.

## 7. Functionalization in Position 7

**Scheme 61:**
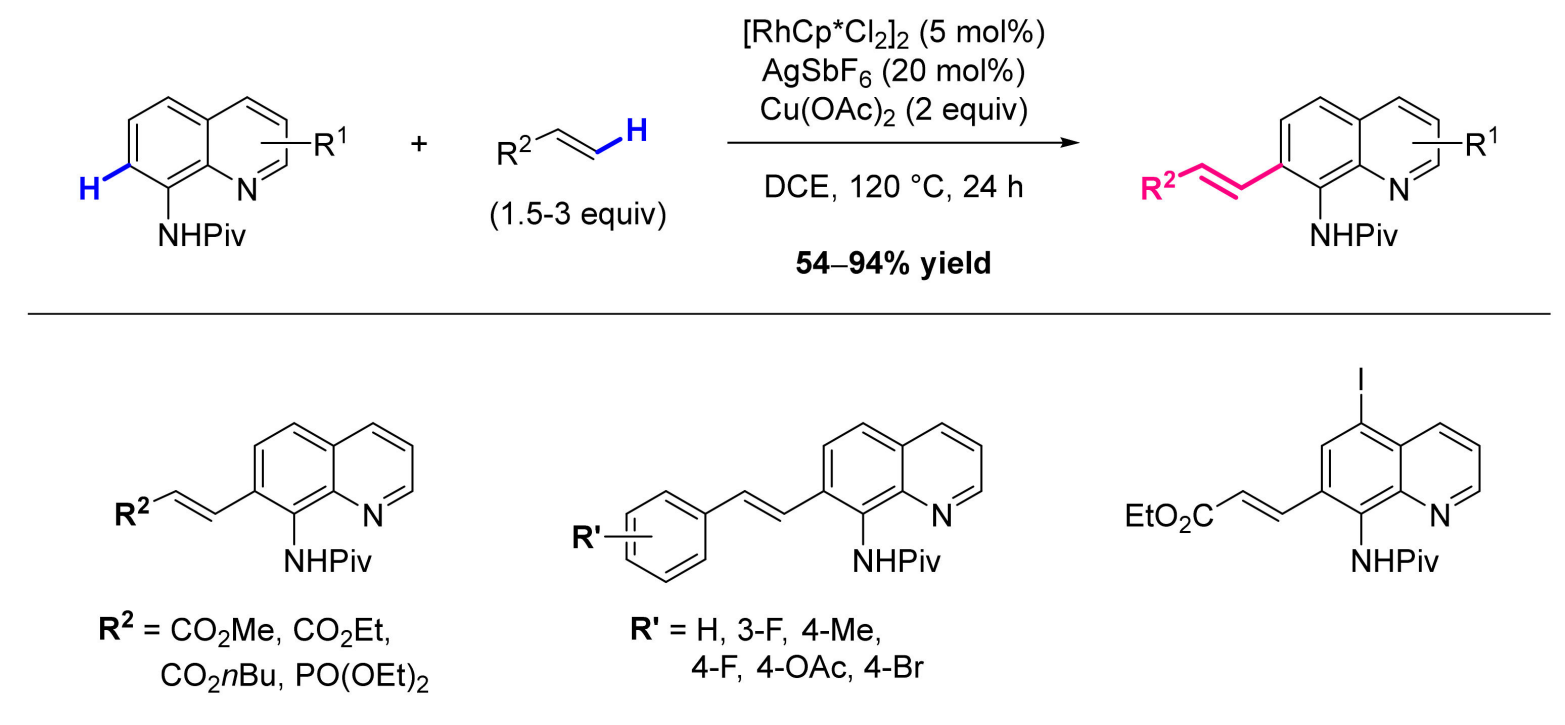
Rh-catalyzed DG-assisted C7 alkenylation of quinolines with acrylates and styrenes.

Just as C6, C7 can also be considered as an orphan position since, to the best of our knowledge, no specific study was realized towards its selective functionalization. However, with the help of a DG, quinolines can be readily functionalized in position 7, as exemplified by the work of Shi and colleagues who, as previously discussed in this review, developed a Rh-catalyzed NHPiv-assisted olefination reaction [152]. When this NHPiv directing group is in position 8, quinoline and its 5-iodo derivative can be alkenylated with various acrylates and styrenes in good to excellent 54–94% yields (Scheme 61).

## 8. Functionalization in Position 8

**Scheme 62:**
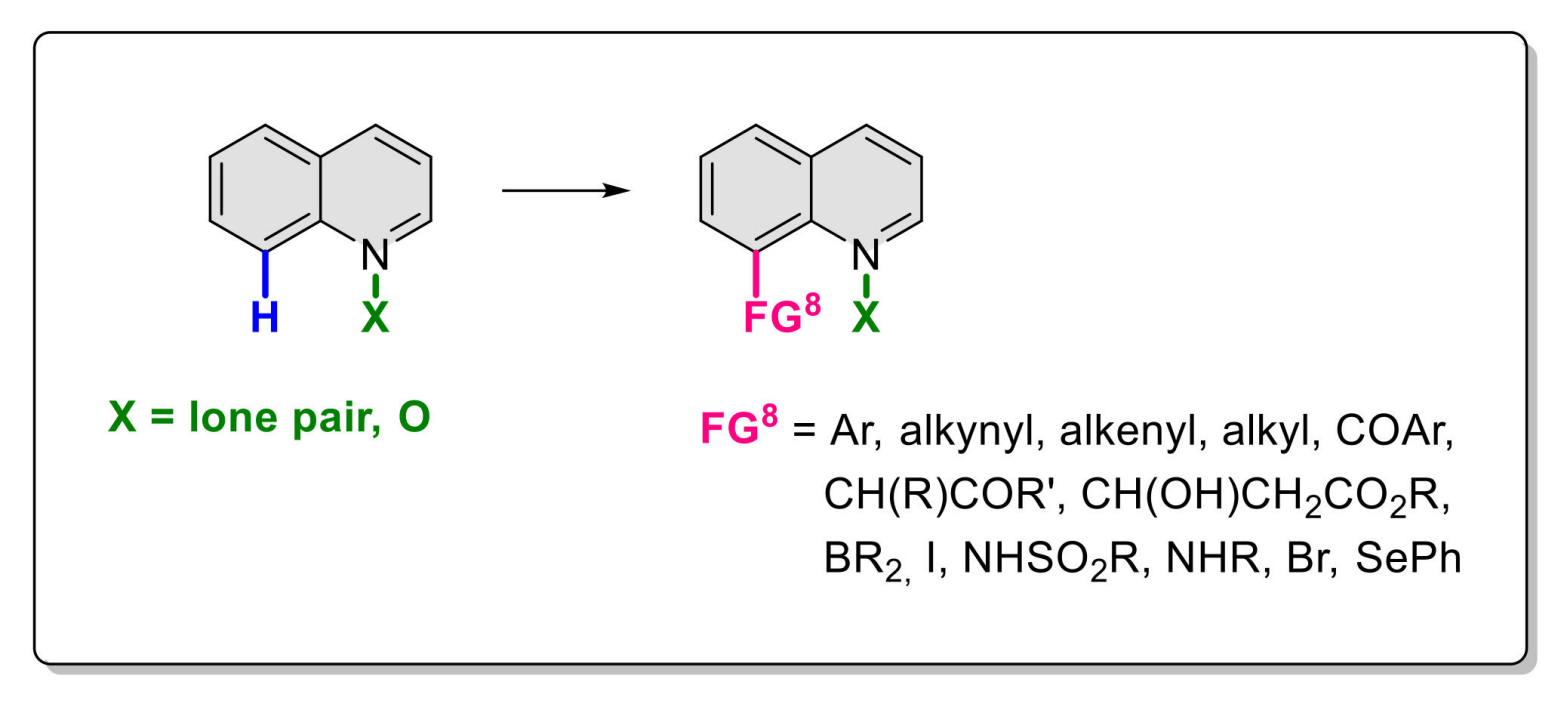
Scope of the selective C-H functionalization achieved in position 8 to date.

Owing to the facility of activation of the C8-H bond, albeit to a lesser extent than C2-H bond, about twenty methods were designed over the last decade to achieve the selective functionalization of position 8 with a wide diversity of aryl, alkynyl, alkenyl, alkyl, acyl, halogeno, borono, amino and seleno functional groups (Scheme 62). The greater part of these reactions is using quinoline *N*-oxides as substrates and, while Pd remains the best option to perform arylations, Rh-based catalysts are the most employed to form other C-C bonds, as well as some of the C-heteroatom bonds.

**Scheme 63:**
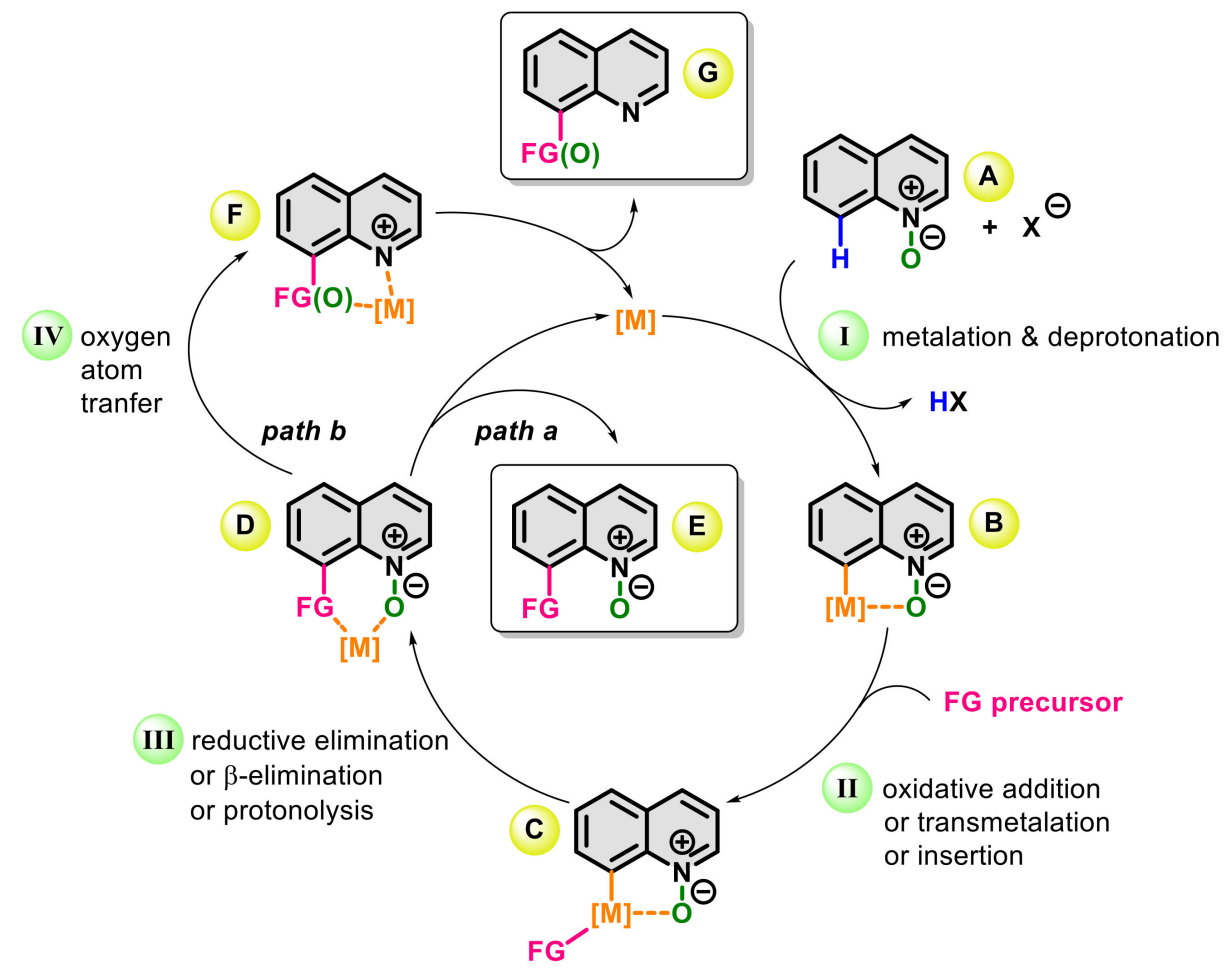
Two main pathways for the C8-H functionalization using *N*-oxide as directing group.

From a mechanistic point of view, functionalization of the quinoline *N*-oxide in position 8 can result from two main pathways (Scheme 63) that are slightly different from the general Scheme 3 depicted above. In this case, after coordination of the metal to the oxygen of the oxide in step **I**, subsequent metalation and deprotonation leads to the formation of a five-membered metalacycle **B** which reacts in turn with the FG-precursor to form the key intermediate **C**. After reductive elimination in step **III**, two options can be considered: either the step **IV** is a straightforward decomplexation that leads to C8-functionalized quinoline *N*-oxides **E** (***path a***); or the conditions allow the subsequent formal oxygen atom transfer (OAT) from the *N*-oxide to the FG (***path b***) that results in C8-substituted quinolines **G**.

### 8.1. Formation of C-Ar Bonds

**Scheme 64:**
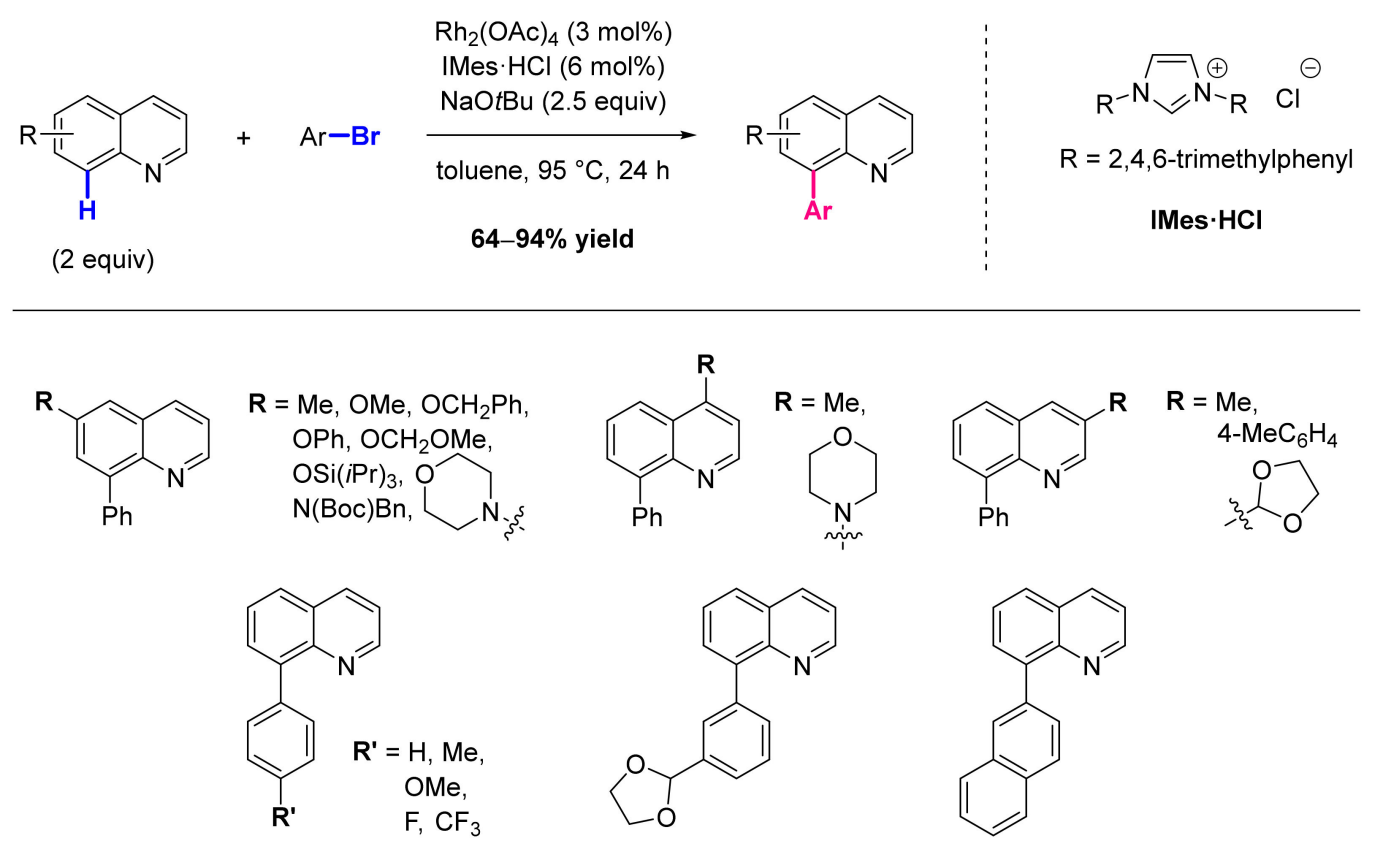
Rh-catalyzed C8 arylation of quinolines with aryl bromides.

The very first example of C-H functionalization in position 8, which was reported in 2011 by the team of Chang, is the Rh(NHC)-catalyzed arylation of quinolines with bromoarenes [174]. During the optimization of the conditions for the coupling of quinoline (2 equiv) and 4-bromotoluene (1 equiv) in toluene, various Rh-based catalytic systems were screened, and both Rh(OAc)_2_ as catalyst source and IMes as ligand appeared to be crucial to reach high efficiency and C8 vs. C2 selectivity. This reaction tolerates a wide variety of diversely substituted coupling partners, enabling the synthesis of a series of arylated QNs in a yield range of 64 to 94% (Scheme 64). Noteworthy, tricyclic heteroarenes such as phenanthridine, benzoquinoline, acridine and phenazine are also suitable substrates.

The reaction pathway proposed by the authors entails a base-assisted concerted proton abstraction/metalation step, hypothesis that was supported by the measurement of the primary KIE, possibly leading to bimetallic or monomeric Rh-NHC species as key intermediates.

**Scheme 65:**
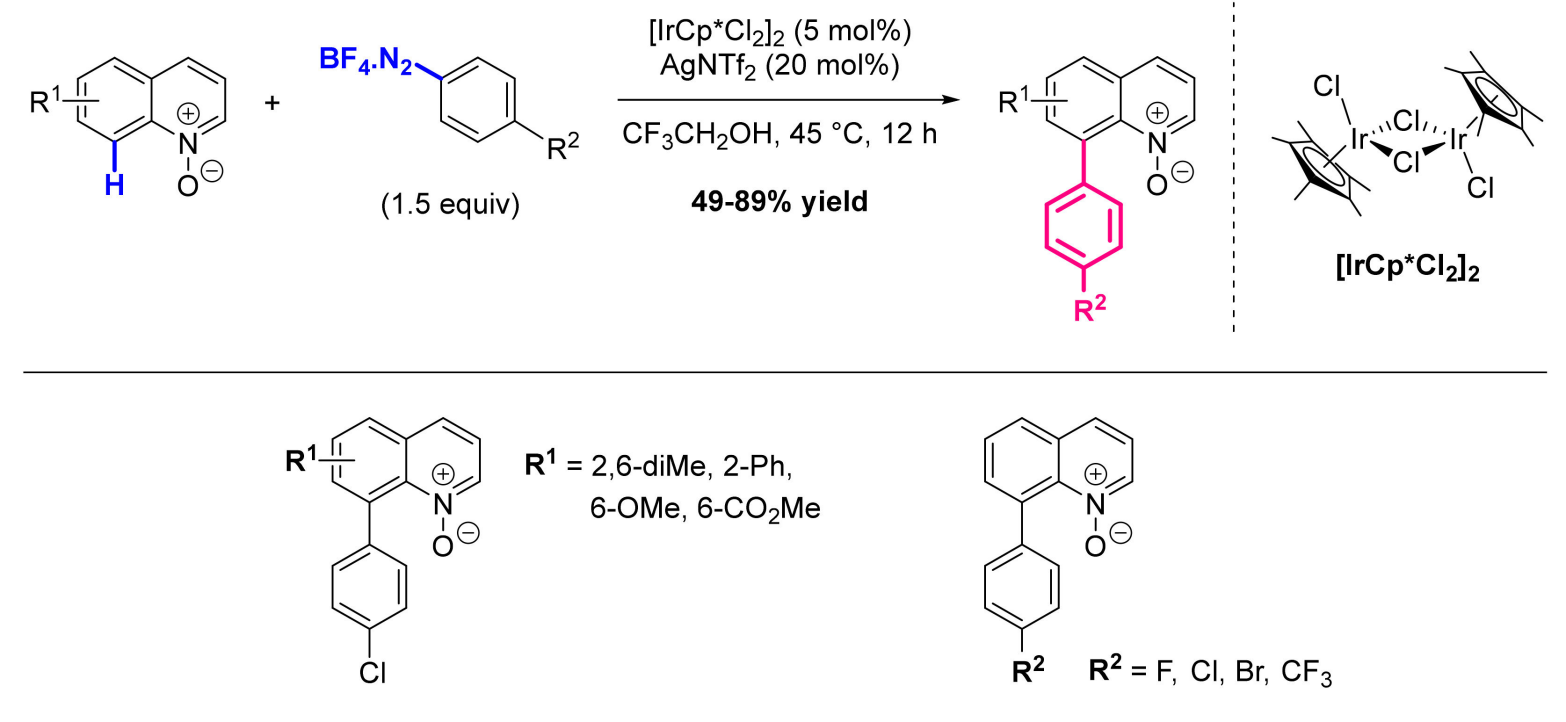
Ir-catalyzed C8 arylation of quinoline *N-*oxides with arenediazonium salts.

Later on, the same group developed another method using Cp*Ir(III) to catalyze the C-H arylation of arenes and alkenes with arenediazonium tetrafluoroborates via an external oxidant-free approach [175]. Full optimization of the reaction conditions as well as assessment of the scope were carried out with benzamides. When enamides were in turn investigated as substrates, the catalytic system had to be adapted and [IrCp*Cl_2_]_2_ (5 mol%) in combination with AgNTf_2_ (20 mol%) proved to be the best option. These conditions were also efficient with QNOs substrates as well when electron-poor coupling partners were used, affording the corresponding 8-arylated products in 49–89% yields (Scheme 65). Noteworthy, the arylation was maintained in position C8 of the QN even in the presence of a phenyl group in position 2.

The mechanism was studied only for benzamides substrates and the reader is invited to consult the original article for a detailed description if desired.

**Scheme 66:**
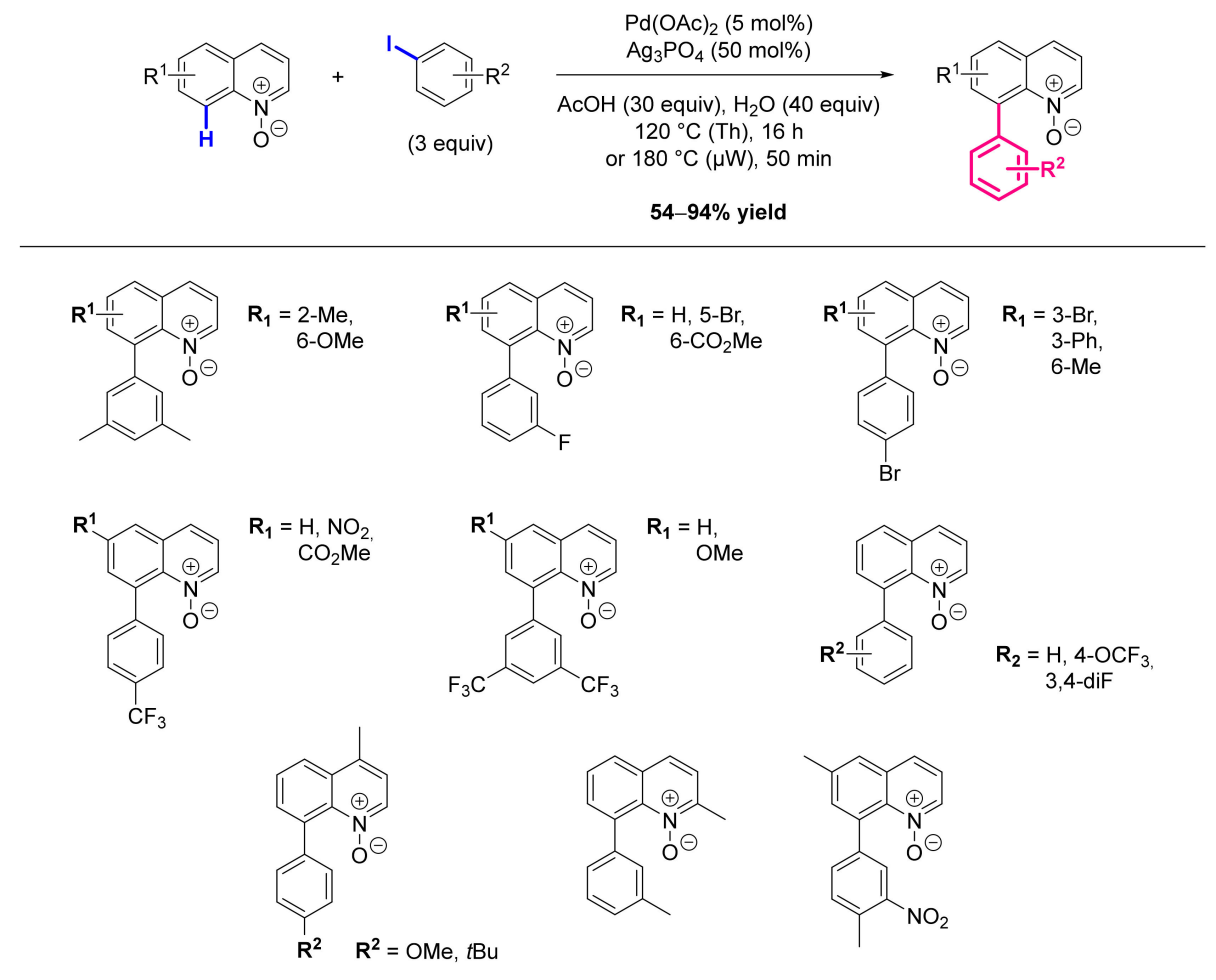
Pd-catalyzed C8 arylation of quinoline *N*-oxides with aryl iodides.

In 2015, Larionov and coworkers reported the first Pd(II)-catalyzed C8 arylation of quinoline *N-*oxides with iodoarenes [176]. Using quinoline *N*-oxide (1 equiv) and 4-trifluoro-iodobenzene (3 equiv) as model substrates, screening various catalysts and additives revealed that the use of Pd(OAc)_2_ (5 mol%), Ag_3_PO_4_ (0.5 equiv) as oxidant and a mixture of AcOH/H_2_O as solvent significantly improved both the efficiency and the selectivity. This reaction exhibits a broad synthetic scope and led to the preparation of 8-arylquinolines diversely substituted on both QN and phenyl rings in yields ranging from 54% to 93% for the thermal conditions (Th), and from 69 to 94% under microwave irradiation (µW) in less than one hour (Scheme 66). Worthy of note, a one-pot procedure that includes oxidation of the substrate with H_2_O_2_, C-H functionalization and reduction of the intermediate with H_3_PO_4_ was also developed to allow the straightforward transformation of QNs into 8-arylquinolines within 3 h.

Extensive experimental and theoretical mechanistic studies were undertaken in order to rationalize this unusual C8 vs. C2 selectivity. The overall observations led to the conclusion that AcOH plays a crucial role for the C-H bond cleavage because it acts as a non-innocent ligand that directs the rate-determining cyclopalladation-step to the C8 position.

**Scheme 67:**
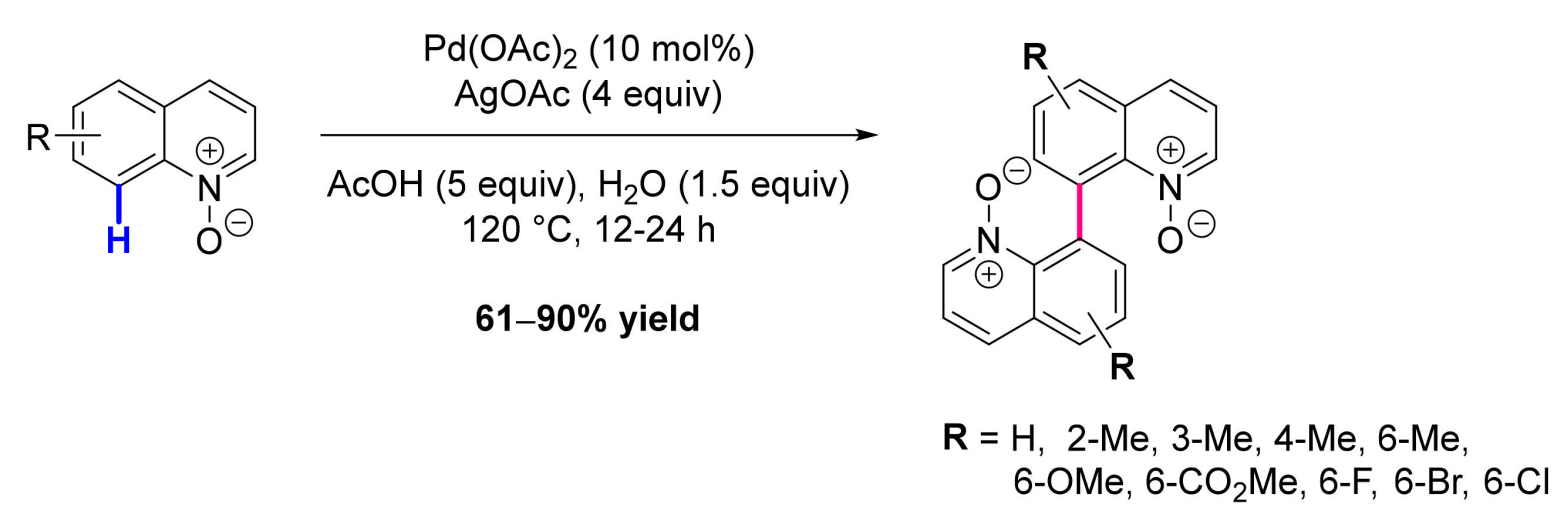
Pd-catalyzed C8 dimerization of quinoline *N*-oxides.

Shortly after, the same team reported a modified version of this Pd(II)-catalyzed C-H activation leading to the homocoupling of quinoline *N*-oxides [177]. To this purpose, various catalysts, solvents and oxidants were screened and a mixture of Pd(OAc)_2_ (10 mol%) and AgOAc (4 equiv) in AcOH/H_2_O (5:1.5) appeared to be optimal conditions. The scope of the reaction was assessed with diversely substituted QNOs, which resulted in the synthesis of the corresponding dimers in 61–90% yields (Scheme 67). Interestingly, this reaction can be successfully carried out on a gram scale.

The mechanism proposed by the authors is a Pd(II)/Pd(IV) process that matches with the *path a* described above in the Scheme 63, with the silver salt acting as an oxidant to regenerate the catalytic species. In line with earlier observations, the pivotal role of acetic acid in step **I** and the accelerating effect of water addition was confirmed. Further mechanistic investigations, including kinetic and Hammet studies, revealed that the turnover-limiting step is not the cyclopalladation but more likely the reductive elimination in step **III**.

**Scheme 68:**
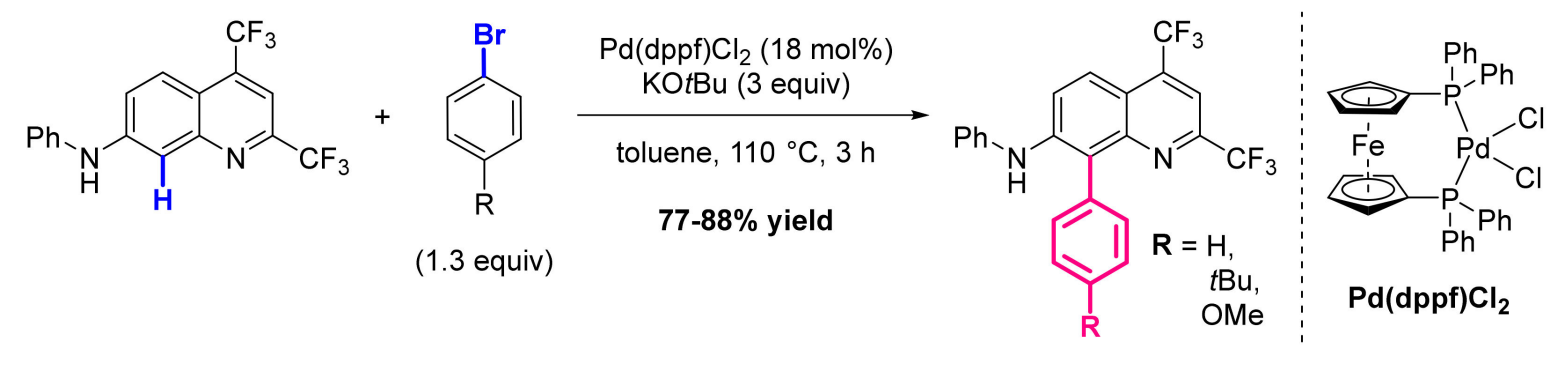
Pd-catalyzed arylation of quinolines with aryl bromides.

The last example of C8-arylation was described in 2019 by Fuchi and Karasawa who developed the synthesis of quinoline-based fluorescent probes thanks to the Pd(II)-catalyzed direct C-H activation of quinolines with aryl bromides [178]. This reaction, which was firstly observed as a side reaction during a Buchwald-Hartwig cross-coupling, was optimized through the screening of various Pd sources and ligands, which led to the selection of Pd(dppf)Cl_2_ (18 mol%) as the best catalyst. Under the optimized conditions, three arylated products were obtained in 77% to 80% yields (Scheme 68).

The mechanism proposed by the authors corresponds to the general mechanism depicted above in Scheme 63, involving a Pd(II)/Pd(IV) catalytic cycle.

### 8.2. Formation of Other C-C Bonds

#### 8.2.1. Without Incorporation of Oxygen (*Path A*)

**Scheme 69:**
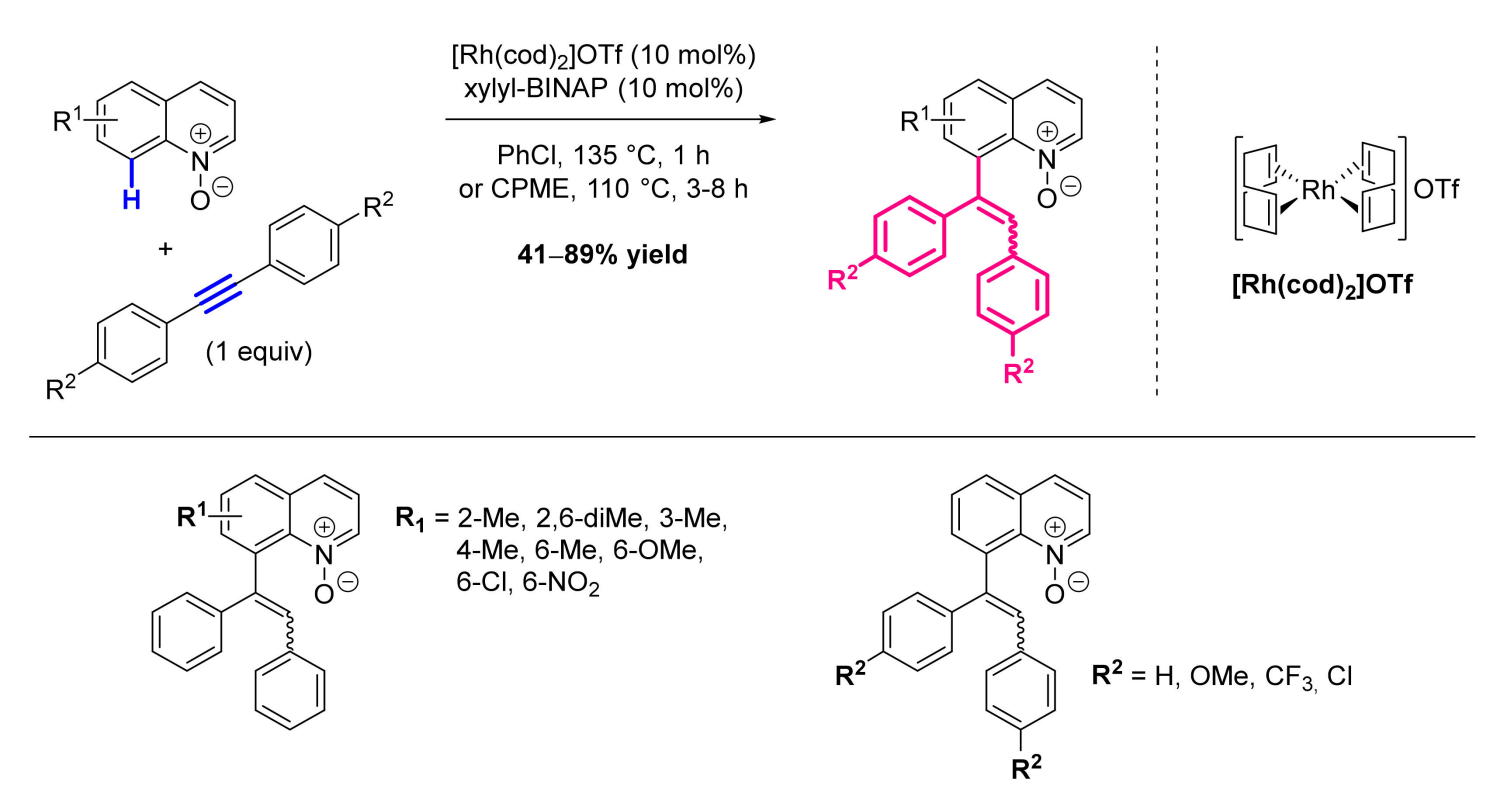
Rh-catalyzed C8 alkenylation of quinoline *N*-oxides with alkynes.

In 2014, Shibata and Matsuo were the first ones to explore the direct C8-H alkenylation of quinoline *N*-oxides with diarylalkynes promoted by a cationic Rh(I) catalyst [179]. The model reaction of quinoline with diphenylacetylene (1 equiv) was optimized through the screening of various [Rh(cod)_2_]X catalysts (10 mol%) and ligands (10 mol%), which demonstrated that the combination of [Rh(cod)_2_]OTf and xylyl-BINAP was the most efficient in chlorobenzene at 135 °C, as well as in cyclopentyl methyl ether (CPME) at 110 °C. Under these optimized conditions, several diarylacetylenes and QNs were used as coupling partners to afford a series of alkenylated products in 41% to 89% yields (Scheme 69). However, the desired products were obtained as an E/Z mixture of diastereoisomers with an E/Z ratio ranging from 1:1 to >20:1, and EWGs on the aryl moiety appeared to lower the reactivity. Interestingly, the final *N*-oxides were readily reduced into the corresponding QNs using PCl_3_.

Deuteration experiments revealed that the C-H bond cleavage is reversible at both the C2 and C8 positions. The authors suggest that the alkyne insertion only occurs in C8 position due to the stability of the five-membered metalacycle corresponding to structure **B** in Scheme 63.

**Scheme 70:**
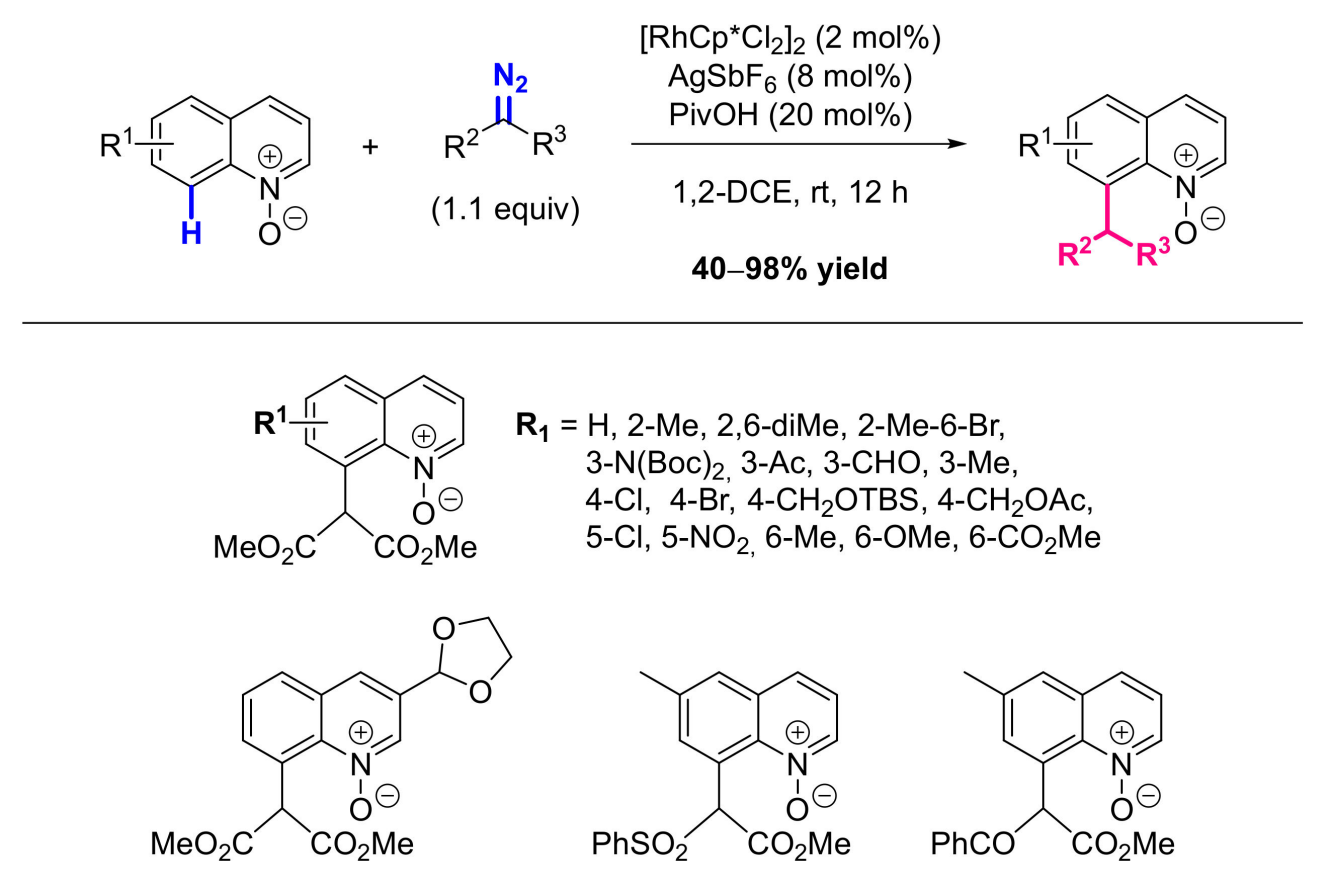
Rh-catalyzed C8 alkylation of quinoline *N*-oxides with diazo malonates.

The same year, Chang’s team developed two types of C8-H functionalization of quinoline *N*-oxides using cationic Rh(III) catalytic systems which, unlike the preceding method, have the advantage of proceeding at room temperature [180]. The first method employs diazo malonates (1.1 equiv) to achieve the C8 alkylation and, after the full optimization of the reaction parameters, a mixture of [RhCp*Cl_2_]_2_ (2 mol%), AgSbF_6_ (8 mol%) and PivOH (20 mol%) in DCE proved to be the best conditions. This reaction tolerates a wide variety of diversely substituted QNOs as substrates, thus enabling the synthesis of 8-alkylated products in 40–98% yields (Scheme 70).

The mechanism proposed by the authors corresponds to the *path a* described above in the Scheme 63, where the coordination of the diazo reagent, followed by the release of N_2_ and migratory insertion of Rh, affords a 6-membered rhodacycle as the key intermediate **D**. From there, protonolysis gives the desired product **E** and regenerates the catalyst. Kinetic isotope-labelling experiments showed that the C-H bond cleavage in step **I** was involved in the rate-determining step.

**Scheme 71:**
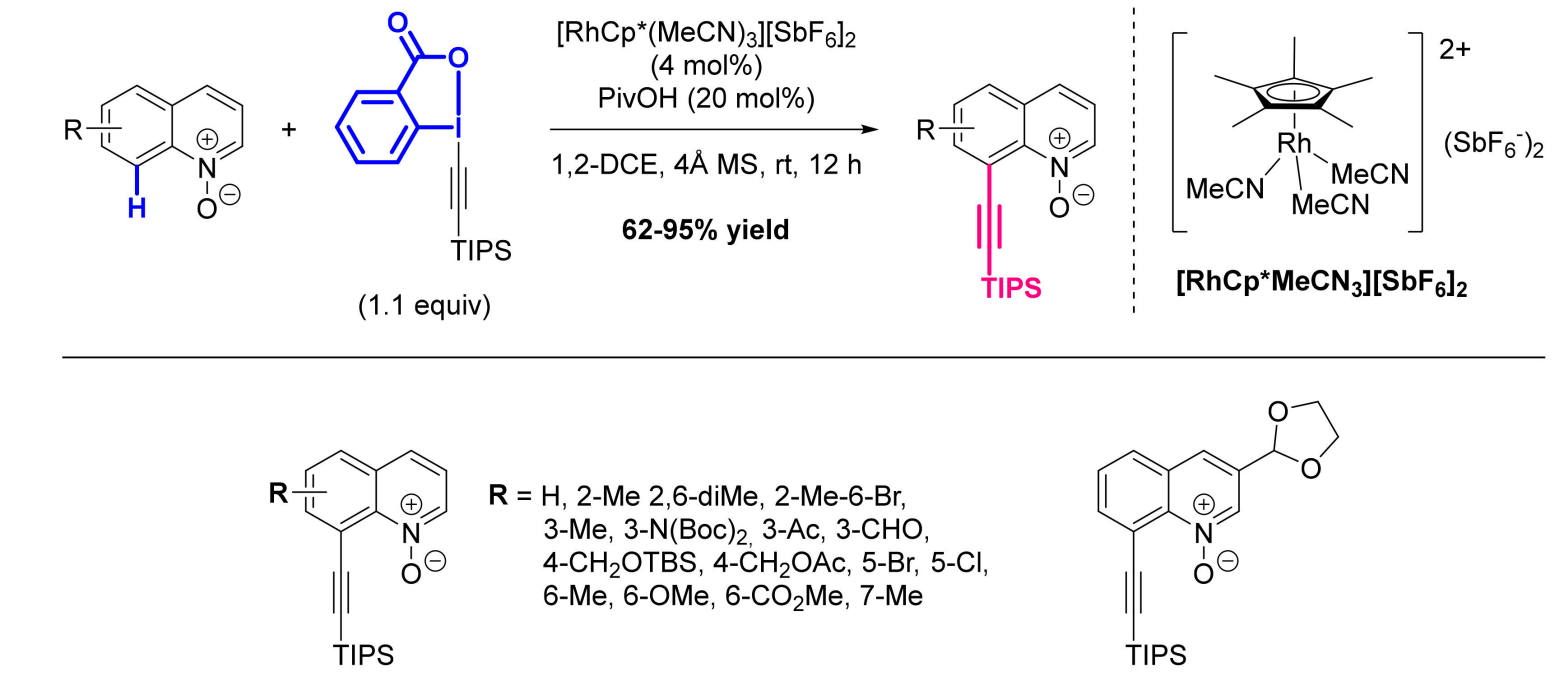
Rh-catalyzed C8 alkynylation of quinoline N-oxides with hypervalent iodine reagents.

The second method leads to the C8 alkynylation using hypervalent iodine reagents (1.1 equiv) for which it was found that using the pregenerated catalyst [RhCp*(MeCN)_3_][SbF_6_]_2_ and molecular sieves was required to improve the reaction efficiency. Several QNOs derivatives were tested as substrates and were smoothly converted into the corresponding alkynylated products in good to excellent 62–95% yields (Scheme 71). Interestingly, these alky(ny)lated products can be easily deoxygenated with the use of zinc to recover the corresponding QNs.

**Scheme 72:**
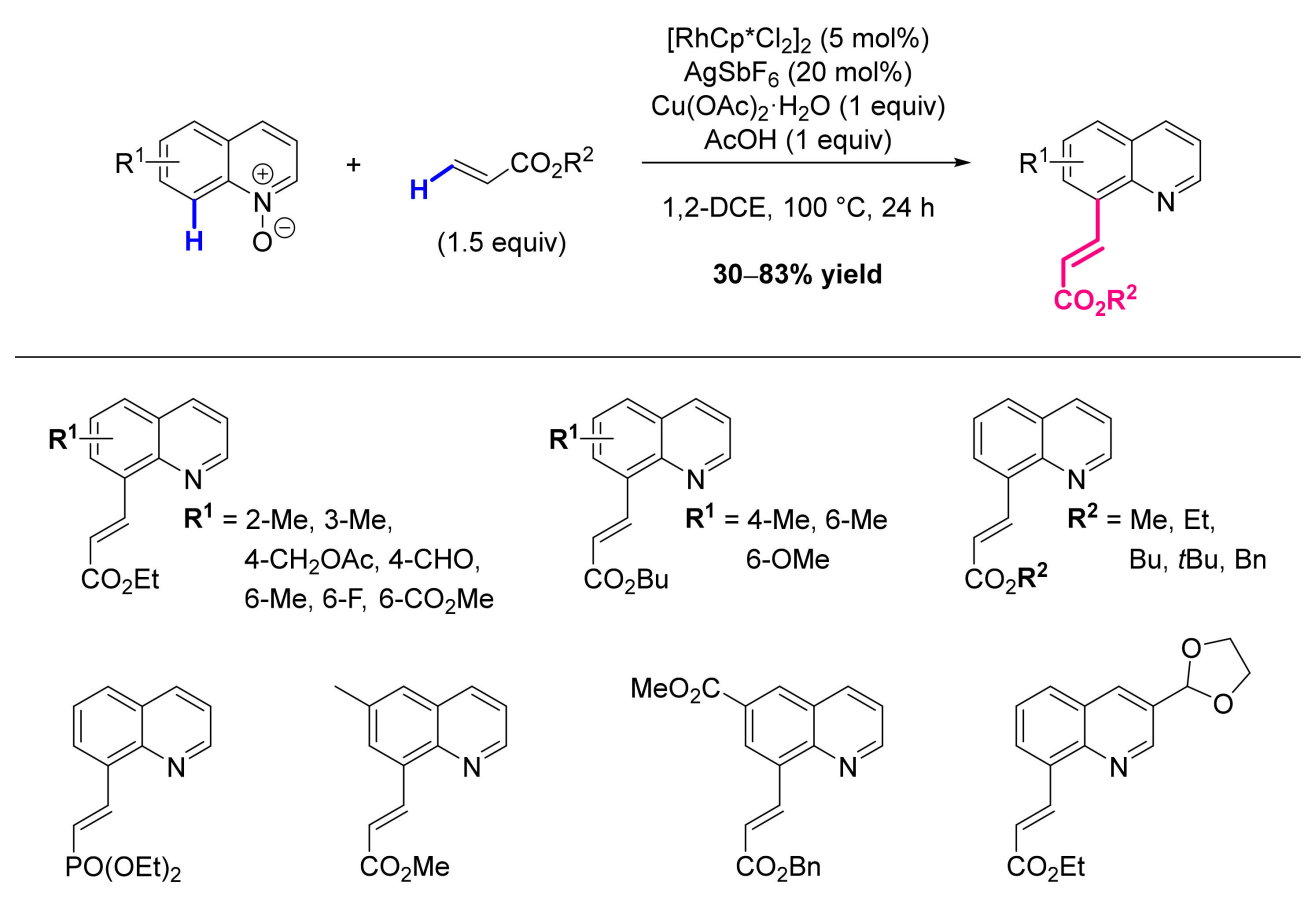
Rh-catalyzed C8 alkenylation of quinoline *N*-oxides with acrylates.

In 2015, the team of Sharma entered the field and made significant contributions, starting with the Rh-catalyzed dehydrogenative coupling of quinoline *N*-oxides with alkenes for which the *N*-oxide moiety is used as traceless directing group [181]. Inspired by the Fujiwara-Moritani alkenylation of arenes with simple alkenes, the authors developed an efficient catalytic system that combines [RhCp*Cl_2_]_2_ (5 mol%), AgSbF_6_ (20 mol%), Cu(OAc)_2·_H_2_O (1 equiv) and acetic acid (1 equiv) in DCE. Investigation of the scope with various QNs and acrylates showed that the outcome of the reaction is not affected by the nature or the position of the substituents on the QN nucleus, leading to a series of 8-alkenylated products in 30–83% yields (Scheme 72). The only exception was the 6-nitro-substituted QNO, which did not lead to the desired product.

**Scheme 73:**
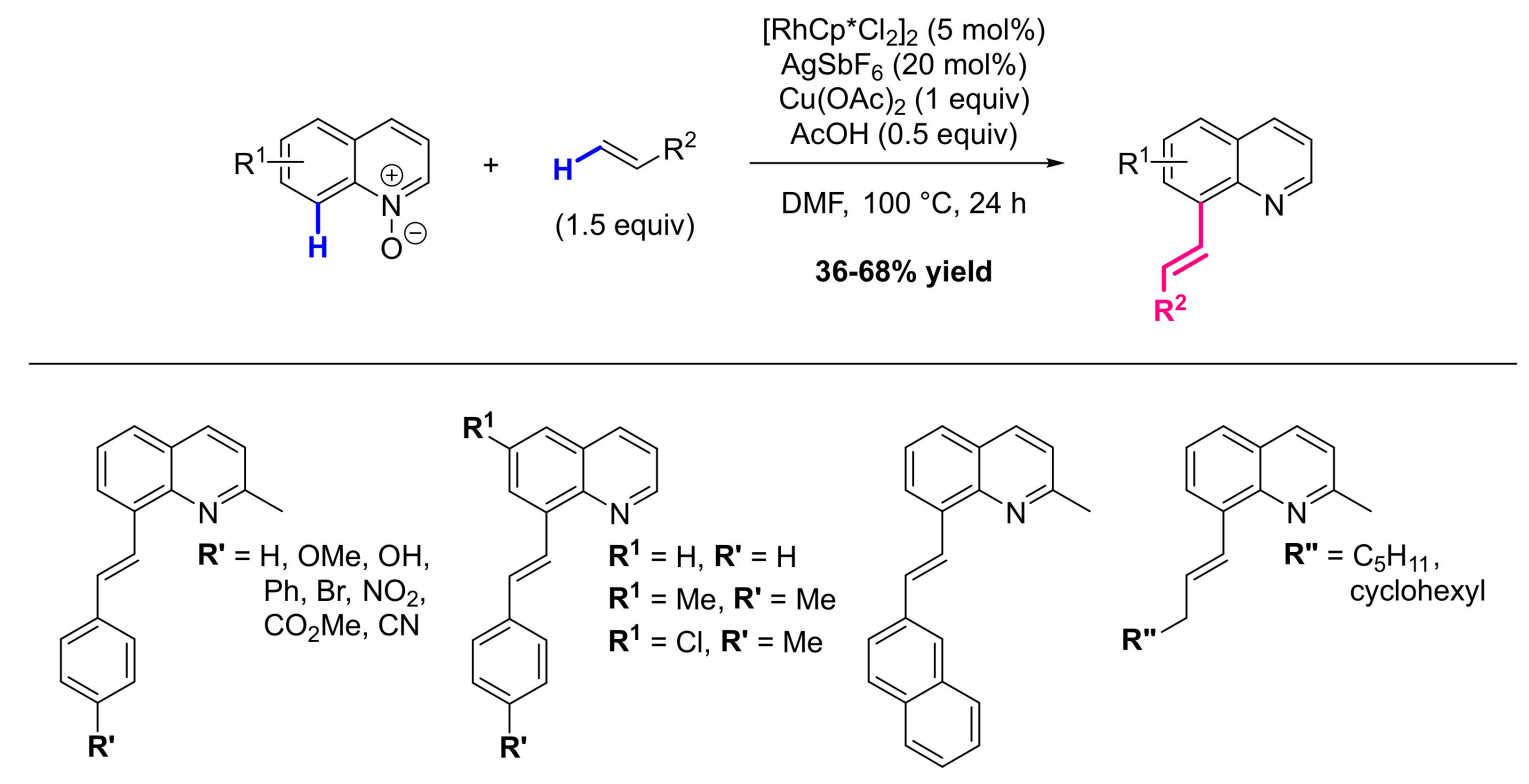
Rh-catalyzed C8 alkenylation of quinolines with styrenes and aliphatic olefins.

Further optimization was required to couple quinolines *N*-oxides with styrene, which revealed that non hydrated Cu(OAc)_2_ and DMF were more efficient in this case. In these adapted conditions, a few QNOs were reacted with several styrene derivatives as well as challenging unactivated olefins, affording a library of 8-olefinated QNs in yields ranging from 36 to 68% (Scheme 73).

After intensive experimental investigations, the authors proposed a mechanism that is similar to the *path a* described above in the Scheme 63, starting with the formation of the five-membered rhodacycle intermediate **B**, which was synthesized and characterized by X-ray and NMR for the first time. Coordination of the olefin, followed by its insertion leads to a seven-membered rhodacycle that undergoes β-hydride elimination to yield intermediate **D**. From there, elimination of HCl and oxidation with Cu(OAc)_2_ is necessary to deliver the desired product **E** and to close the catalytic cycle. Measurement of the KIE indicates that the cleavage of the C-H bond in step **I** is involved in the rate-determining step.

**Scheme 74:**
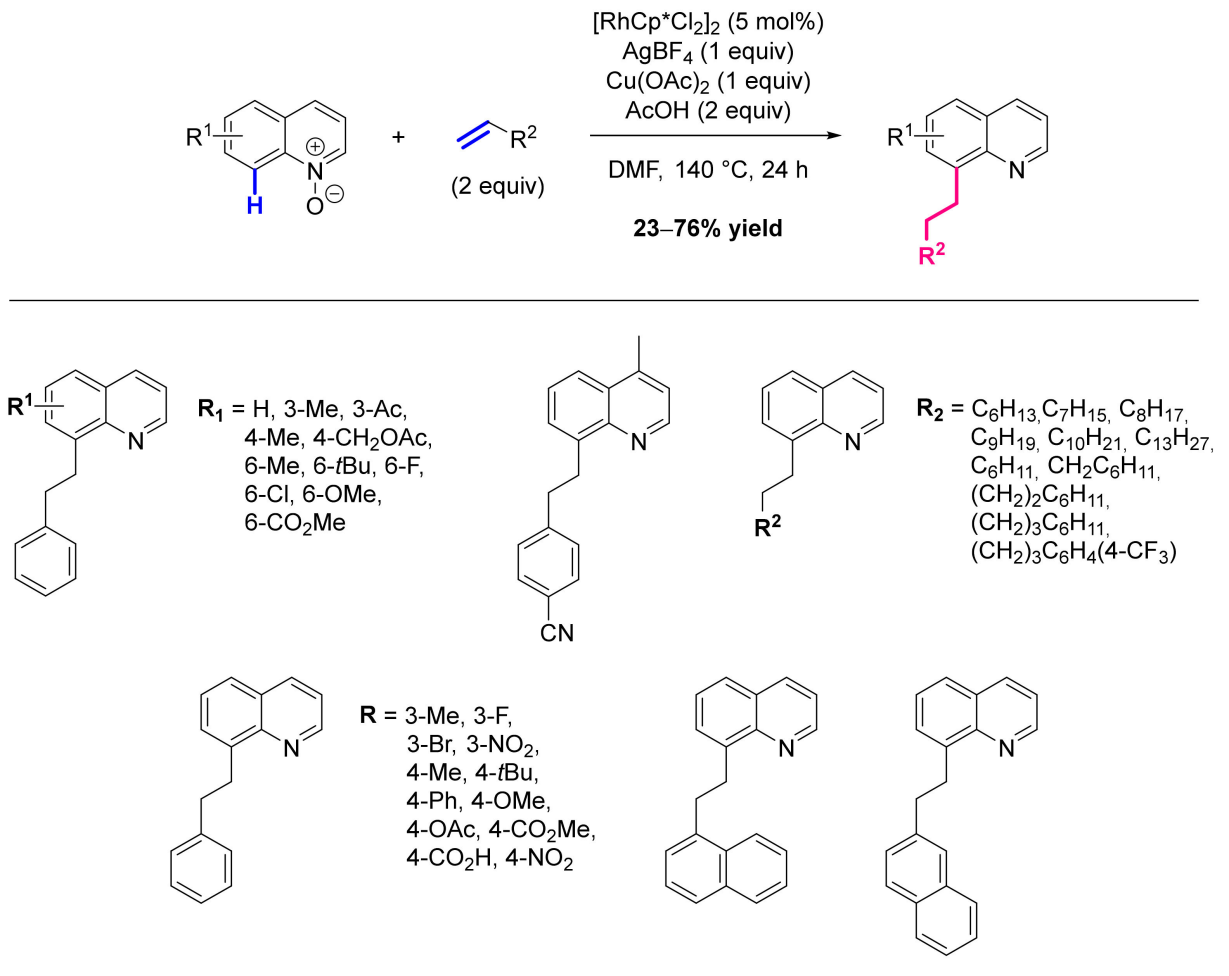
Rh-catalyzed C8 alkylation of quinolines with styrenes and aliphatic olefins.

Later on, exploiting a side reaction observed during their previous study on alkenylation, the same group developed an analogous Rh-catalyzed alkylation method using again the *N*-oxide moiety as traceless directing group [182]. The coupling of quinoline *N*-oxide and styrene (2 equiv) with [RhCp*Cl_2_]_2_ (5 mol%) in DMF was taken as a model reaction to screen different additives and it was found necessary to use AgBF_4_ and a closed vessel to promote the alkylated vs. alkynylated product. Under these optimized conditions, a wide range of styrene derivatives and aliphatic olefins, albeit to a lower extent, were tolerated as alkylating reagents, thus affording a large series of C8-alkylated QNs in 23–76% yields (Scheme 74). The only exception was the 2-methyl-QNO, which did not furnish the desired product, most probably because of steric hindrance.

The mechanism proposed by the authors is much more complex than the general mechanism described above in Scheme 63, with a first cycle corresponding to the former alkenylation reaction, followed by a second cycle that leads to the saturated C8 alkylated quinoline. Extensive mechanistic studies confirmed that the first step of C-H cleavage/carbometalation is reversible and rate-limiting. Moreover, DMF proved to be involved in the reduction of the alkenylated intermediate through the generation of formic acid.

**Scheme 75:**
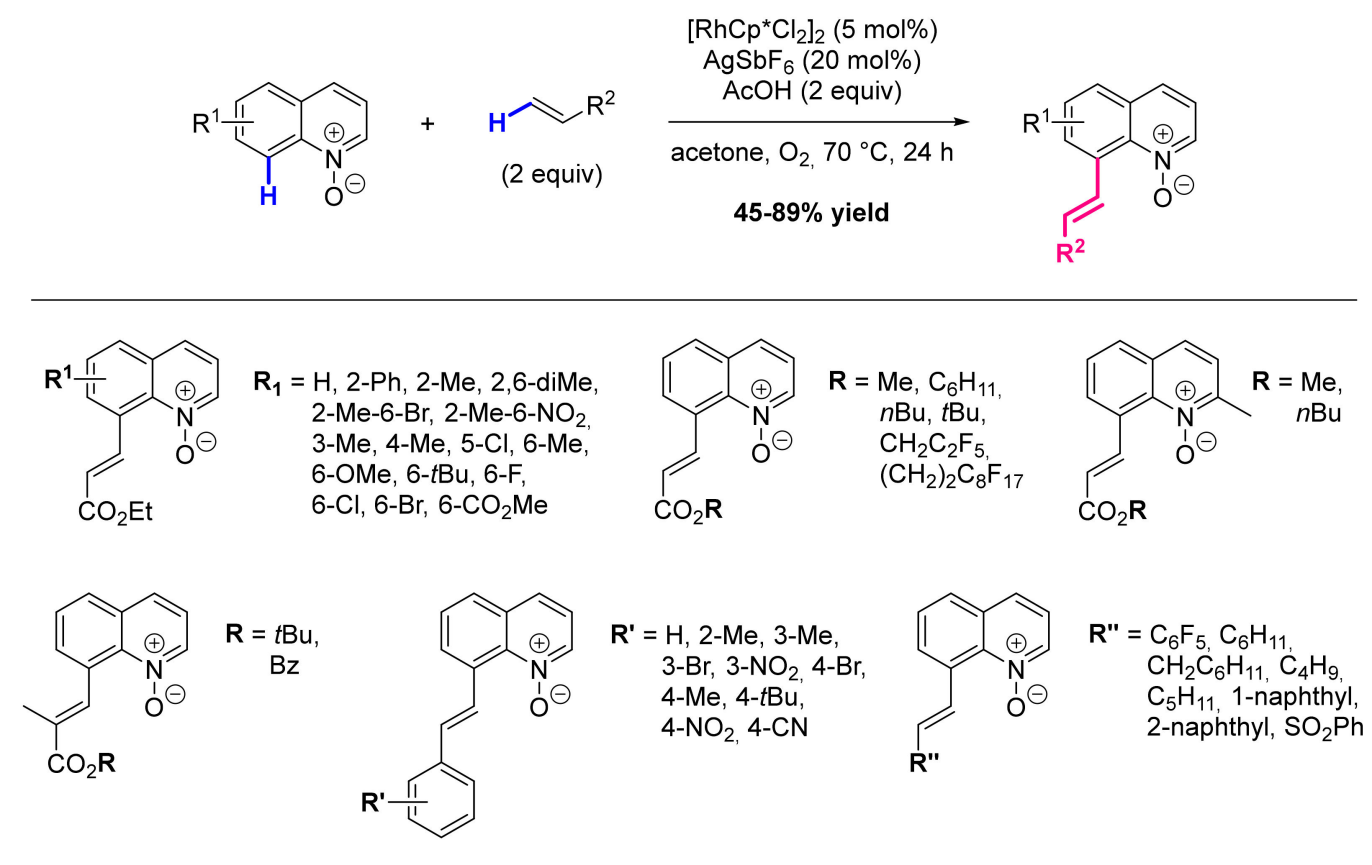
Rh-catalyzed C8 alkenylation of quinoline N-oxides with olefins.

The last piece of work reported by Sharma’s group in 2019 is a greener version of their previous alkenylation protocol for which the copper acetate was advantageously replaced by molecular oxygen as a cheaper and cleaner oxidant. However, by contrast with their previous results, the *N*-oxide is not reduced under these conditions [183]. Refinement of the conditions showed that better results are obtained at lower temperature (70 °C vs 100 °C) in acetone. The generality of the reaction was evaluated with various acrylates, including those (not shown) derived from bulky alcohols (ie 3-hydroxy adamantine, 9-anthranyl methyl, vitamin E and estrone), as well as styrenes and unactivated olefins, which provided a wide library of alkenylated QNOs in 45–89% yields (Scheme 75). Remarkably, the 2-substituted QNOs reacted smoothly under these conditions. It is worth mentioning too that the *N*-oxide moiety can be subsequently reduced with the use of PhB(OH)_2_.

Further mechanistic investigations were undertaken with the previously isolated rhodacycle complex **B** which was proven to be an active intermediate and was also detected by ESI-MS analysis. It appeared also that while the initial C-H bond cleavage is reversible, it may not be the rate-determining step.

**Scheme 76:**
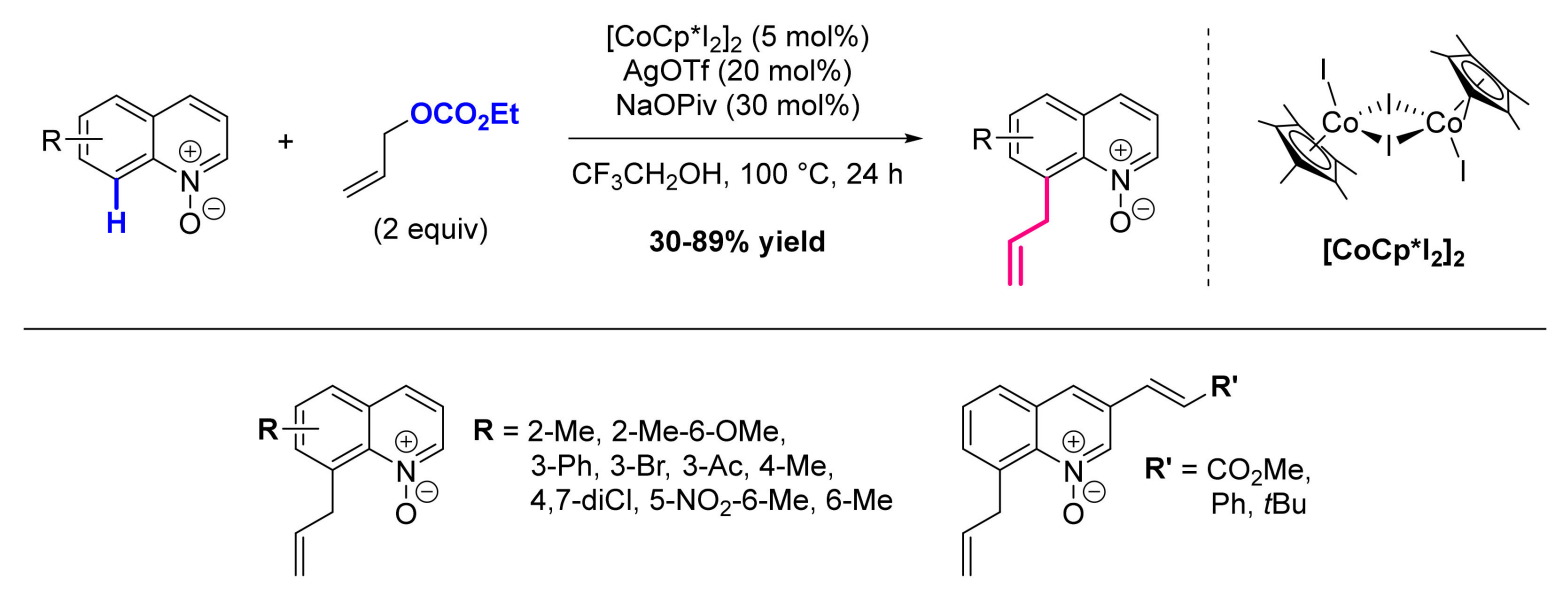
Co-catalyzed C8 allylation of quinoline N-oxides with carbonates.

Meanwhile, Sundararaju and coworkers reported in 2016 a full scope and mechanistic study on Rh- and Co-catalyzed C8 alkylation or allylation of quinoline *N*-oxides [184]. The allylation was firstly investigated with allylcarbonate (2 equiv) in trifluoroethanol and, after the screening of various catalysts, silver salts and carboxylate bases, the system composed of [CoCp*I_2_]_2_ (5 mol%), AgOTf (20 mol%) and NaOPiv (30 mol%) proved to be the most efficient. Under these optimized conditions, several diversely substituted QNOs were tested as substrates and the corresponding C8 allylated products were obtained in 30–89% yields (Scheme 76). The electron poor substrates turned out to be less reactive than the electron rich ones, while sensitive Ac or NO_2_ groups are well tolerated.

**Scheme 77:**
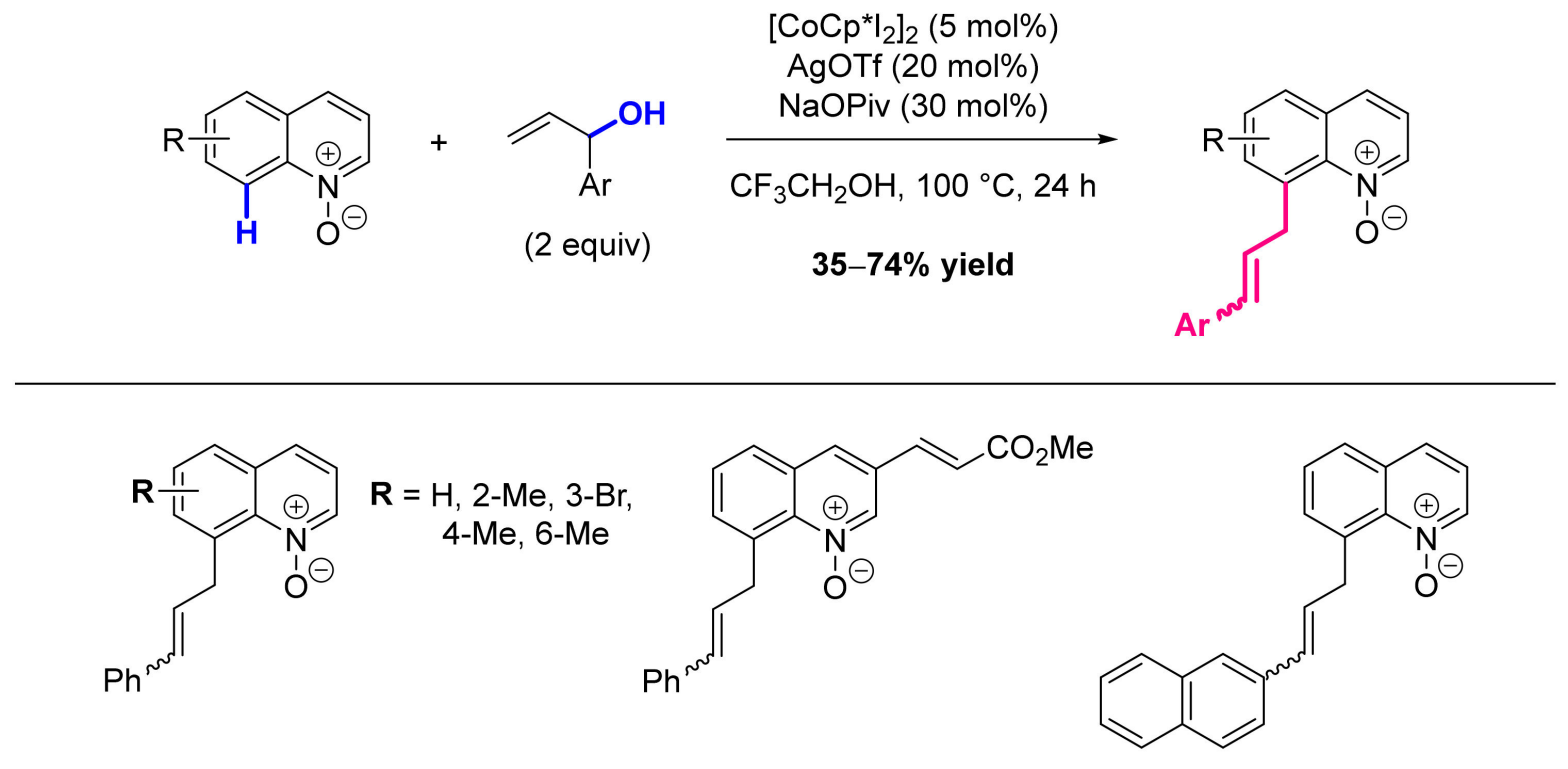
Co-catalyzed C8 allylation of quinoline N-oxides with allyl alcohols.

These exact same conditions were found to be also efficient with allylic alcohol as coupling partner and several α-vinylbenzyl alcohols were therefore used to test the generality of the reaction, leading to the desired products in 35–74% yields (Scheme 77).

**Scheme 78:**
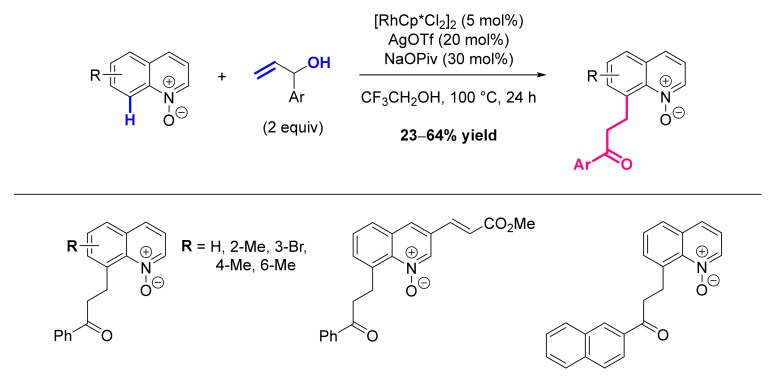
Rh-catalyzed C8 alkylation of quinoline *N*-oxides with allyl alcohols.

Finally, when the Co(III) catalyst was switched to [RhCp*Cl_2_]_2_ while keeping all other reaction parameters constant, the reaction provided β-aryl ketones instead of unsaturated products, as it was anticipated by the authors, leading to a series of C8 alkylated quinoline *N*-oxides in 23–64% yields (Scheme 78). The presence of sensitive groups such as Br and CO_2_Me led to moderate results in both Co- and Rh-catalyzed methods.

The authors, inspired by previous studies on Co(III) and Rh(III)-catalyzed allylation, assumed that the former would follow a β-hydroxy-elimination whereas the latter would proceed via a β-hydride-elimination. This difference in reactivity was further supported by DFT calculations.

**Scheme 79:**
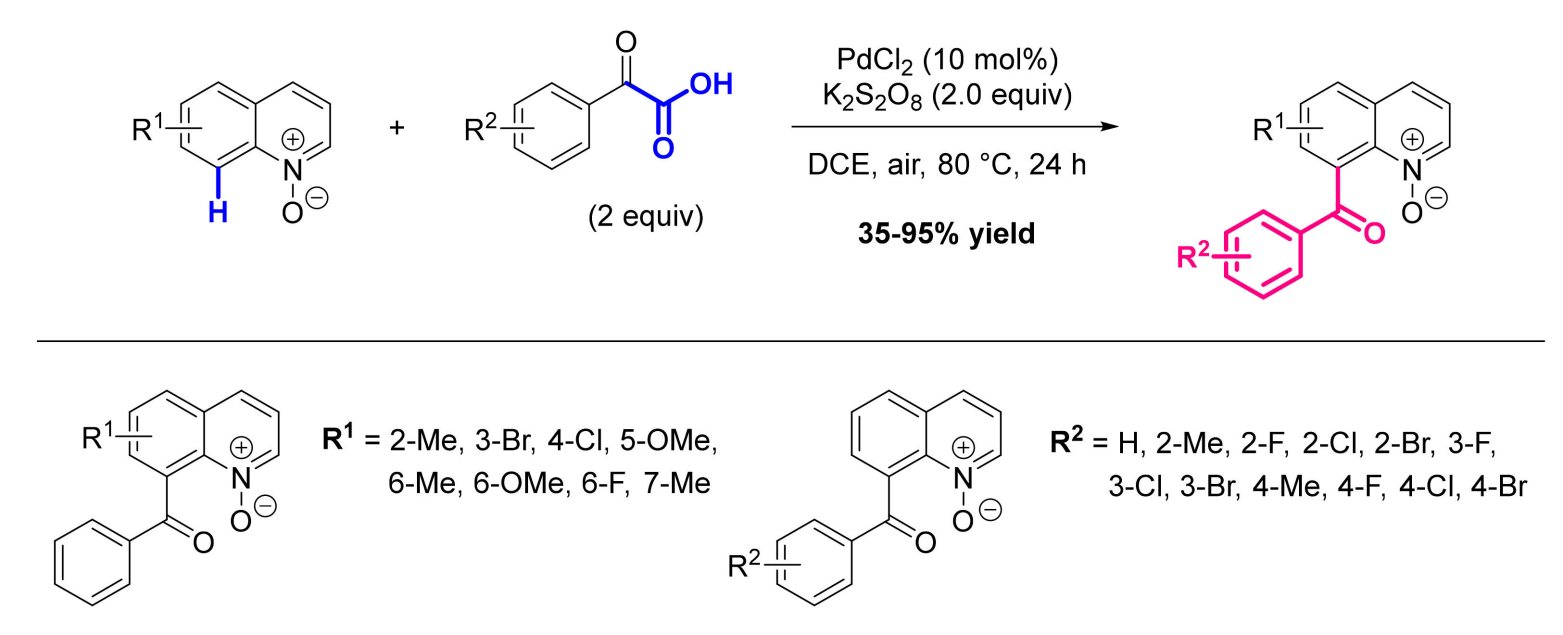
Pd-catalyzed C8 acylation of quinoline *N*-oxides with α-oxocarboxilic acids.

The last possibility of C-C bond formation described in this section is the C8 acylation, which was firstly developed by the group of Cui and Wu in 2016 [185]. Using 2-oxo-2phenylacetic acid as aryl donor, various reaction parameters such as the catalyst, the oxidant and the temperature, were optimized and PdCl_2_ (10 mol%), K_2_S_2_O_8_ (2 equiv) in DCE at 80 °C proved to be the best conditions. Diversely substituted QNOs and α-oxocarboxylic acids were used as coupling partners and were converted into the desired C8 acylated QNOs in 35–95 % yields (Scheme 79). In general, QNOs bearing EDGs were more reactive than those with EWGs.

After a few control experiments, the mechanism proposed by the authors matches with the *path a* described above in Scheme 63, for which the α-oxocarboxylic acid decarboxylates after its coordination to Pd to afford the key intermediate **C**, and K_2_S_2_O_8_ is necessary to reoxidize the Pd (0) into Pd(II) after the RE step **III**.

**Scheme 80:**
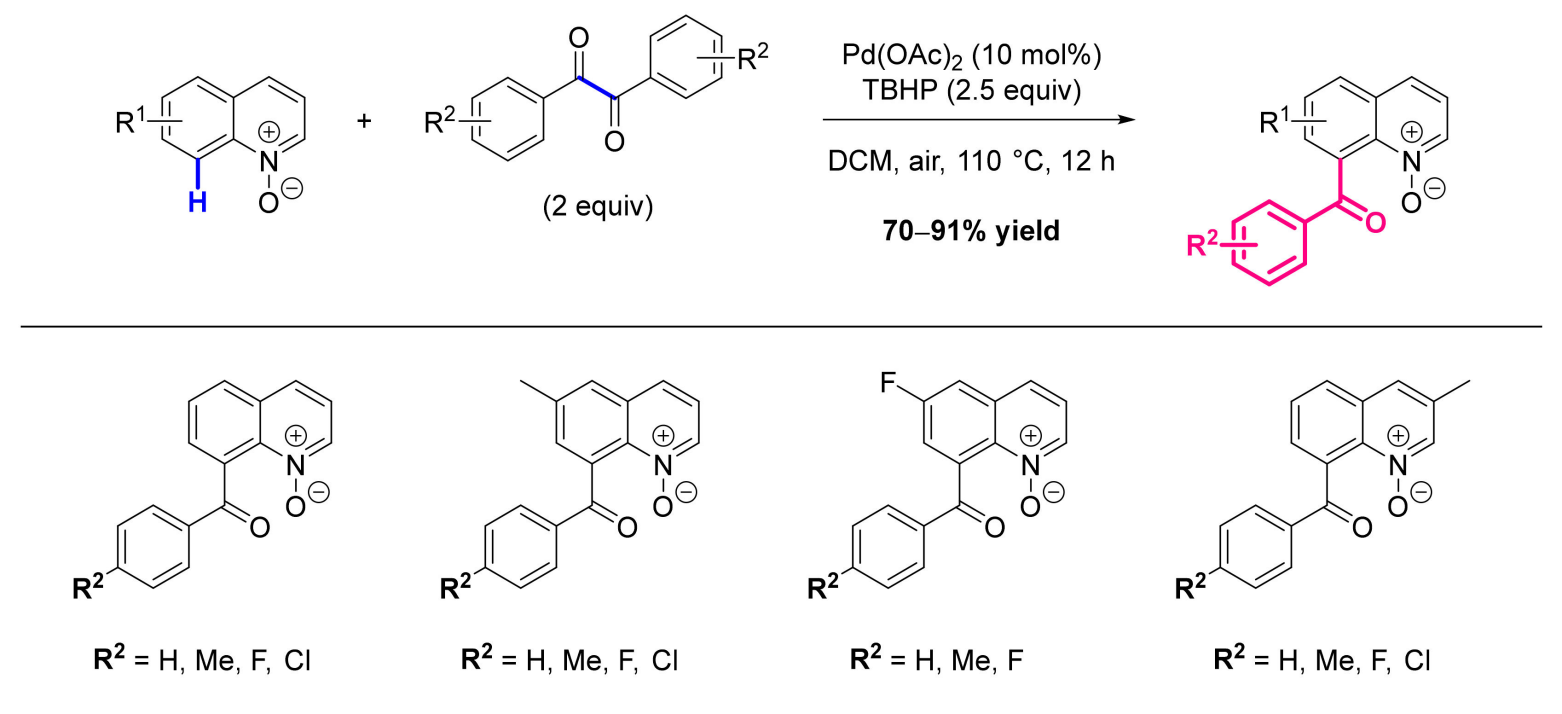
Pd-catalyzed C8 acylation of quinoline *N*-oxides with α-diketones.

More recently, the group of Wang reported in 2019 another Pd-based method to achieve the C8 acylation of quinoline *N*-oxides with α-diketones that are considered as benzoyl radical sources under oxidative conditions [186]. Optimization of the conditions using diphenyl ethanedione (2 equiv) in DCM was carried out through the screening of various Pd-based catalysts and oxidants, which revealed that Pd(OAc)_2_ (10 mol%) and TBHP (2.5 equiv) was the best combination. Under these conditions, various QNOs and α-diketones were tested and led to the synthesis of the corresponding C8 acylated products in good to excellent 70–91% yields (Scheme 80).

The mechanism proposed by the authors is in agreement with the *path a* described above in Scheme 63 where the FG-precursor is a benzoyl radical generated in situ by the homolytic cleavage of the benzoin with TBHP. This radical oxidizes the cyclopalladated intermediate **B** in step **II**, to afford the key intermediate **C** which then undergoes RE in step **III**.

#### 8.2.2. With Incorporation of Oxygen (*Path B*)

In this subsection were gathered all the methods for which the *N*-oxide moiety acts as both a traceless directing group and an O atom donor, ie. as an internal oxidant. All these reactions proceed via C-H activation/OAT cascade processes corresponding to the *path b* in the general Scheme 63 represented above.

**Scheme 81:**
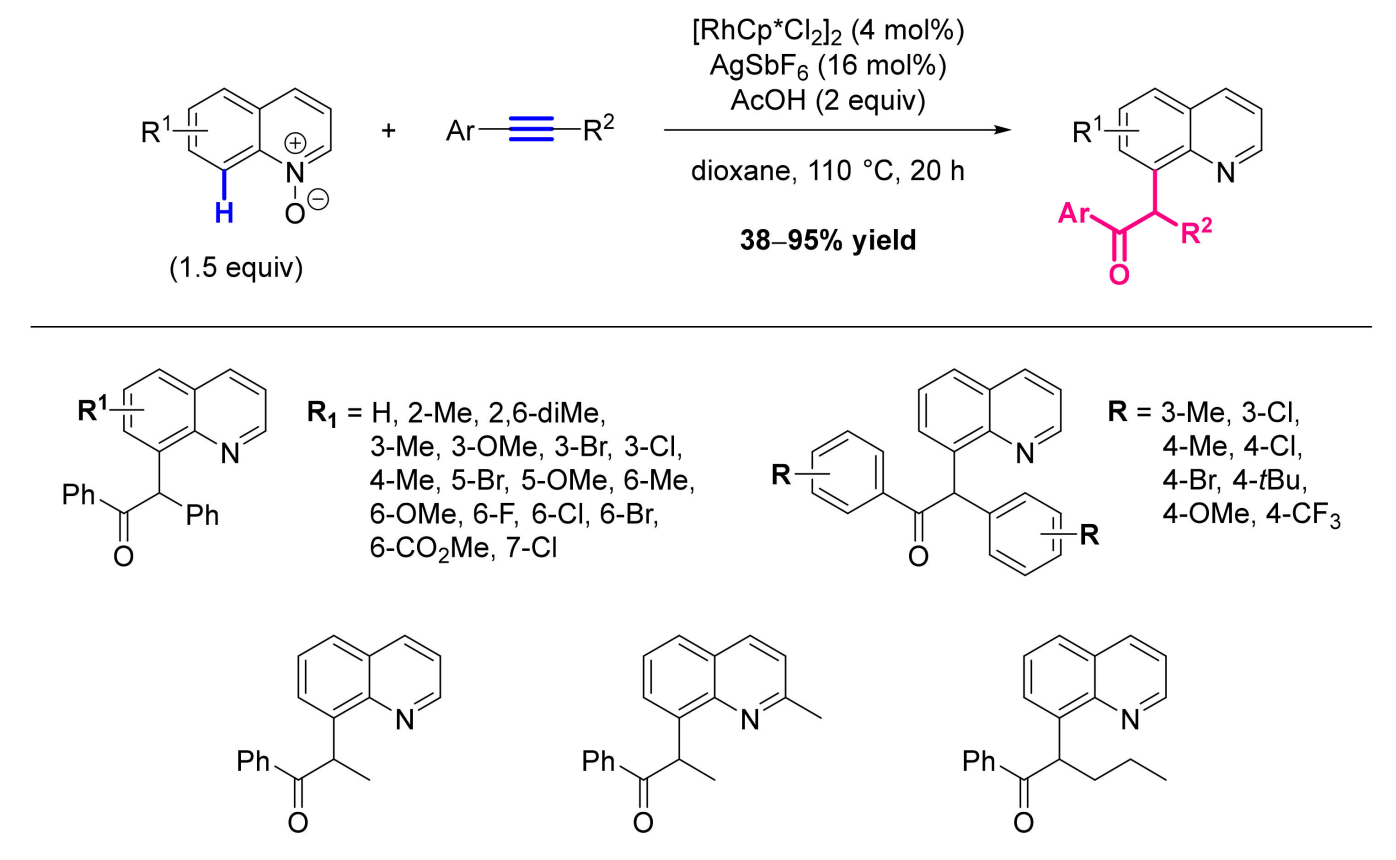
Rh-catalyzed OAT/C8 alkylation of quinoline N-oxides with acetylenes (Li et al.).

In 2014, inspired by the pioneering work of Fagnou, the group of Li was the first one to combine the *N*-oxide assisted C-H activation of quinolines with the OAT to alkynes thanks to Rh catalysis, leading thus to (8-quinolyl)acetophenones with 100% atom economy [187]. A model reaction composed of quinoline *N*-oxide (1.5 equiv), diphenylacetylene (1 equiv) and [RhCp*Cl_2_]_2_/AgSbF6 in a 1:4 ratio was used to test various solvents and catalyst loadings: dioxane proved to have a dramatic beneficial impact and the amount of catalyst can be lowered to 4 mol% without observing negative effect. The generality of this reaction was evaluated with various QNOs and acetylenes, affording a series of α-substituted acetophenones in 38–90% yields (Scheme 81). This reaction turned out to tolerate both EWGs and EDGs, whereas it seems sensitive to steric hindrance at position 7. Moreover, while all diarylacetylenes coupled smoothly, unsymmetrical ones led to two regioisomeric products.

Extensive mechanistic studies have been carried out and the results of these experiments suggest that C-H activation might be an irreversible rate-limiting step, that occurs prior to the OAT, which is very likely an intramolecular process. However, the key intermediate **B** is not a five-membered metalacycle but an unusual rhodium-(III) η3-benzyl intermediate that has been isolated and proved to be a catalytically active species. All these hypotheses and findings were found to be in agreement with the computational DFT study that Li’s group reported thereafter [188].

**Scheme 82:**
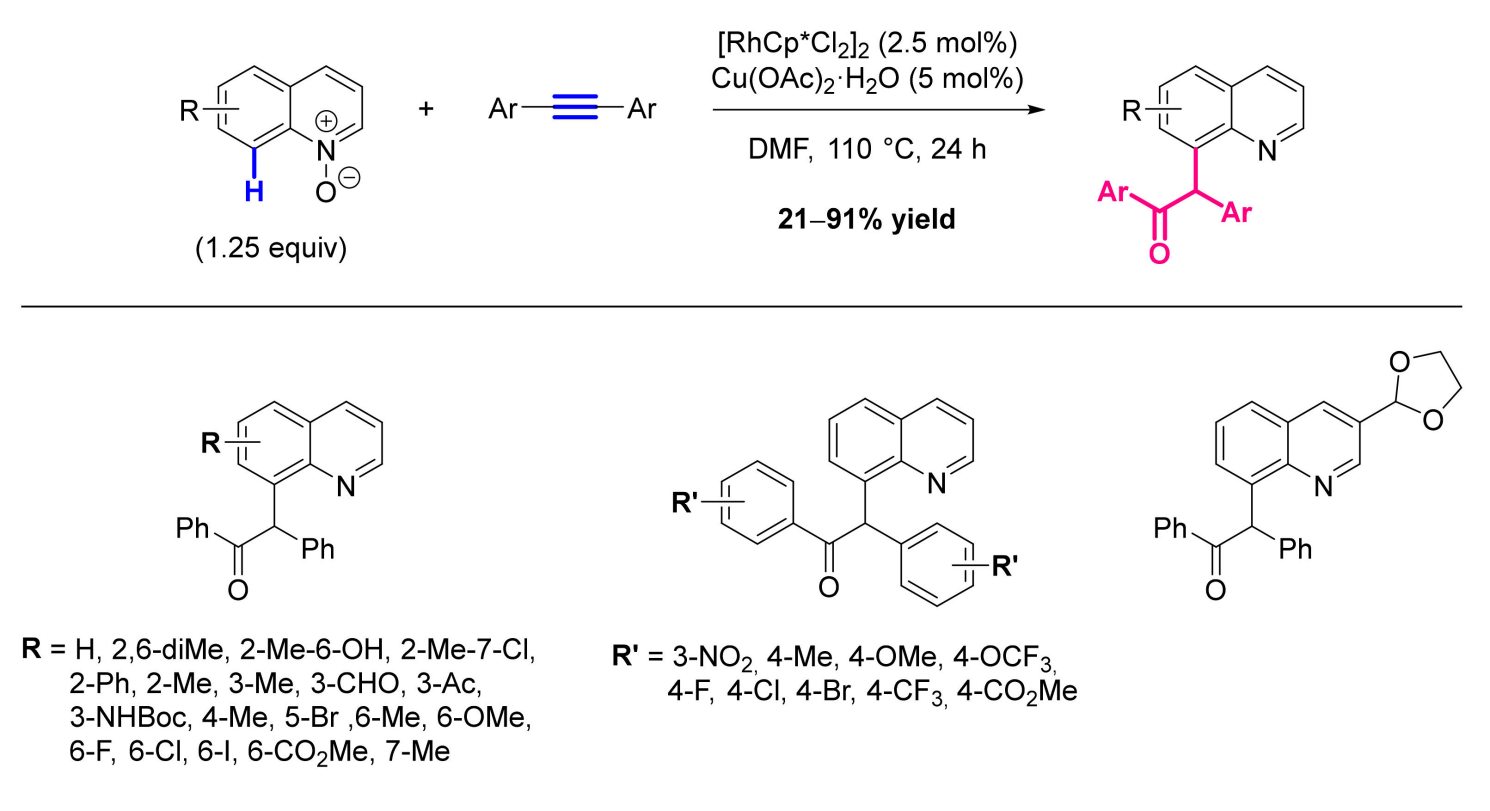
Rh-catalyzed OAT/C8 alkylation of quinoline *N*-oxides with acetylenes (Chang et al.).

Exactly at the same time, Chang and Sharma disclosed a similar traceless [RhCp*Cl_2_]_2_-catalyzed coupling of quinoline *N*-oxides with internal diarylalkynes that combines alkene insertion and OAT in a cascade process [189]. In this case, an extensive screening of the reaction parameters led to the selection of [RhCp*Cl_2_]_2_ (2.5 mol%) and Cu(OAc)·H_2_O (5 equiv) to reach the highest efficiency. The scope of the reaction was investigated with a wide range of QNOs and various symmetrical alkynes, which resulted in the synthesis of the corresponding (8-quinolyl)acetophenones in yields ranging from 21 to 93% (Scheme 82). Interestingly, this reaction has a large functional group tolerance but, as also mentioned by Li’s group, leads to regioisomeric mixtures when unsymmetrical alkynes are used.

Mechanistic studies confirmed that the C-H cleavage would be rate-limiting and that the *N*-oxide moiety is the source of oxygen in the OAT process.

**Scheme 83:**
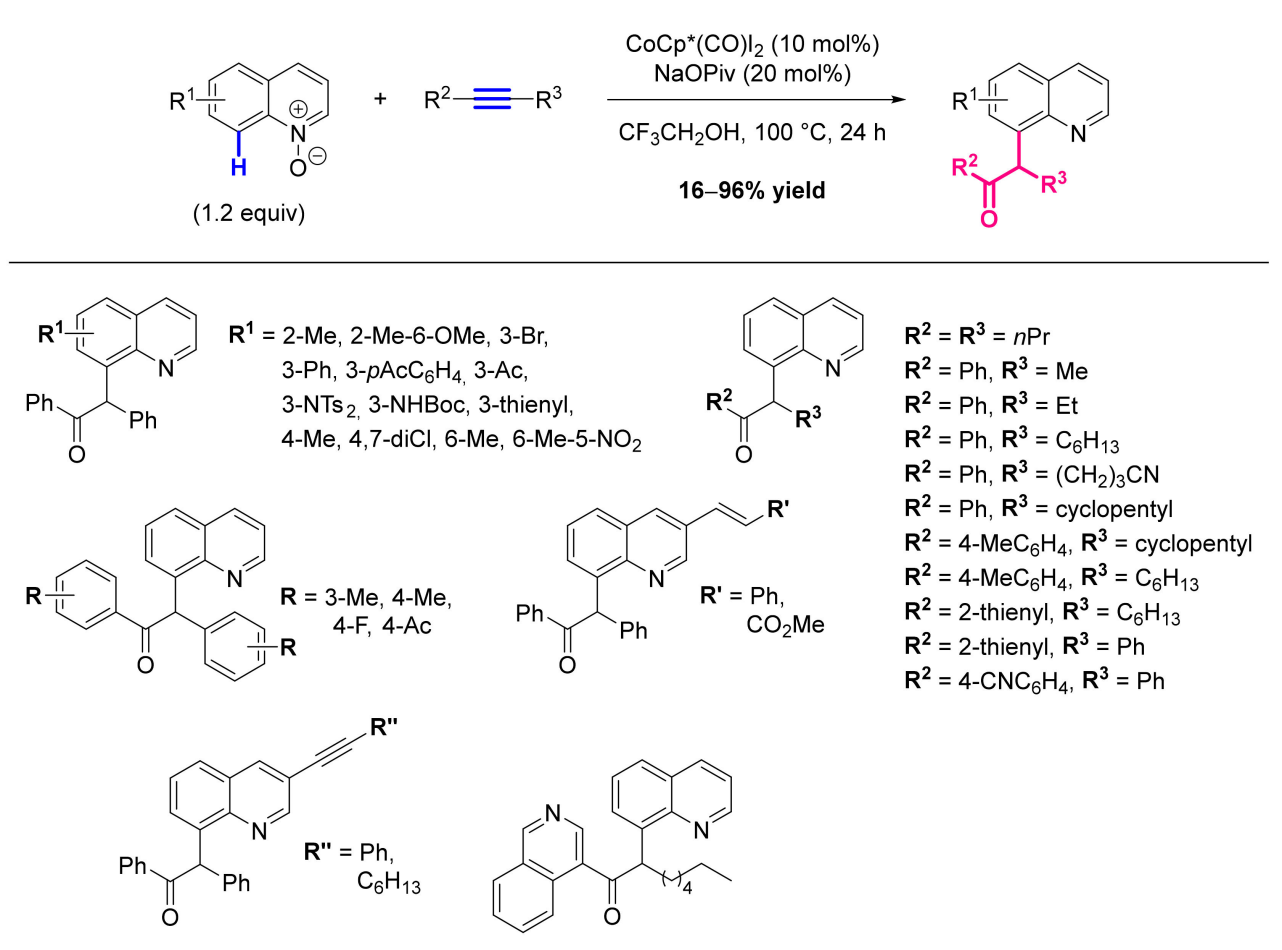
Rh-catalyzed OAT/C8 alkylation of quinoline *N*-oxides with acetylenes.

In 2015, prompted by the desire to use relatively cheap and abundant first row transition metal catalysts, Sundararaju and coworkers developed a comparable co-catalyzed coupling of quinoline *N*-oxides with internal alkynes followed by OAT [190]. After an extensive screening of various Co-catalysts, additives and solvents, the use of CoCp*(CO)I_2_ (10 mol%) and NaOPiv (20 mol%) in trifluoroethanol proved to be the best conditions. When the scope was investigated with diversely substituted QNOs and alkynes, the reaction appeared to possess a wide group tolerance, which led to the synthesis of a large series of products in 16–96% yields (Scheme 83). These conditions proved to offer several advantages compared to the previously reported Rh-catalyzed alternatives: i) aliphatic alkynes are well tolerated as coupling partners, ii) the reaction can be carried out on the gram-scale and iii) unsymmetrical internal alkynes provided a single regioisomer with excellent control of the regioselectivity, except for electronically biased species.

Deuteration experiments suggested that cyclometalation/C-H activation is likely to be irreversible, and that C-H activation followed by OAT is a cascade process. The proposed mechanism was further supported by DFT calculations. It was also shown that efficiency and reactivity of Co(III) catalyst is better than Ir(III) and Rh(III) catalysts towards C(8)-H bond functionalization of QNO.

**Scheme 84:**
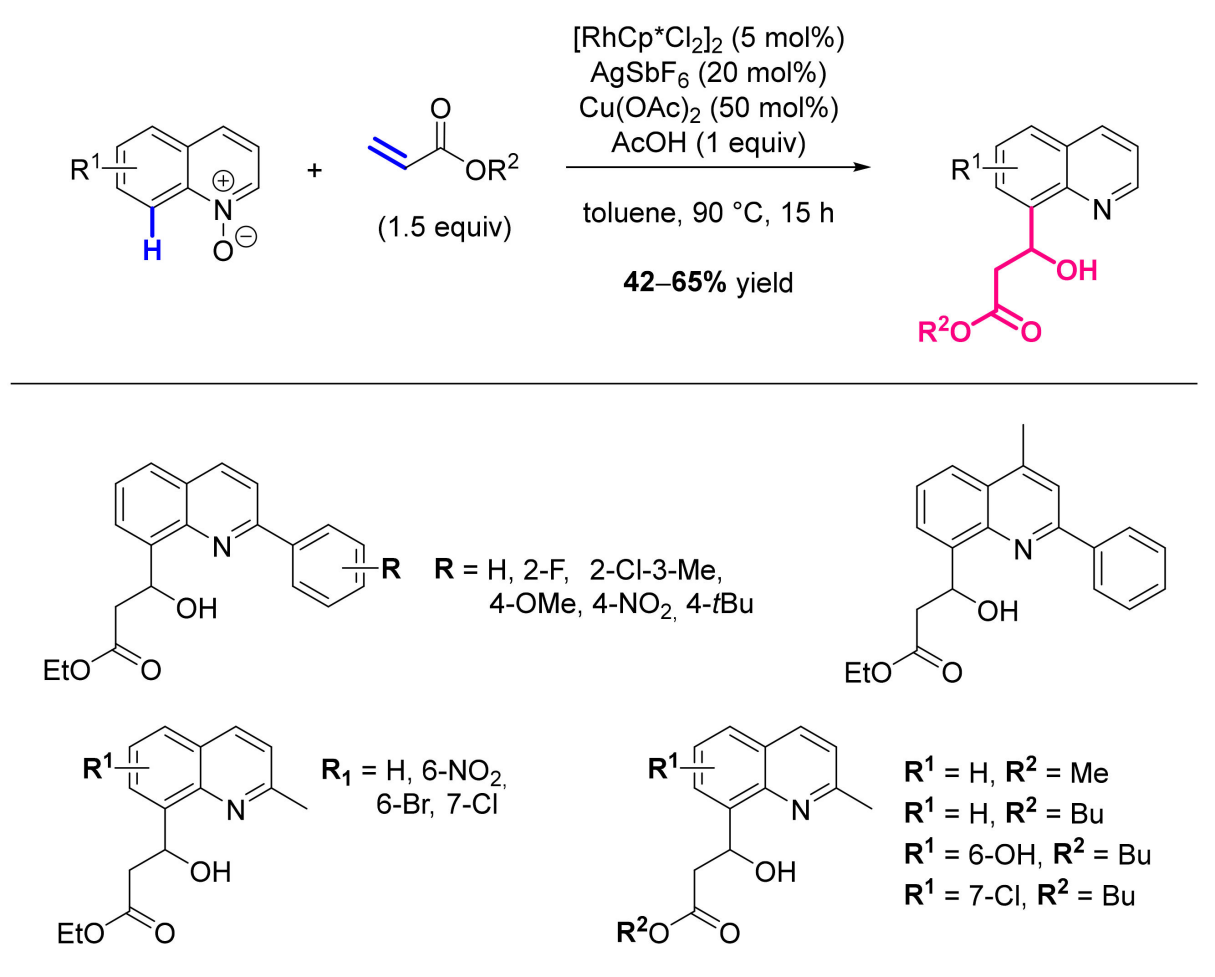
Rh-catalyzed OAT/C8 alkylation of quinoline *N*-oxides with acrylates.

In 2018, taking advantage of a side reaction observed during their previous study [181], the team of Sharma developed Rh-based conditions to achieve the hydroxyalkylation of quinoline *N*-oxides with acrylates via the simultaneous formation of C-C and C-O bonds [191]. The reaction conditions were fully optimized in order to promote the introduction of hydroxyalkyl substituents in position 8 of QNs, which was successfully achieved by using [RhCp*Cl_2_]_2_ (5 mol%), AgSbF_6_ (20 mol%), Cu(OAc)_2_ (0.5 equiv) and acetic acid (1 equiv) in toluene. This reaction tolerates a large diversity of substituents on both coupling partners, leading to the synthesis of a short series of 3-hydroxyquinolin-8-yl propanoates in 45–65% yields (Scheme 84). However, the scope of this reaction is limited to C2-substituted QNOs.

On the basis of experimental mechanistic investigations, and comparison with precedent findings, the authors proposed a mechanism that is analogous to the one reported previously for the olefination [181]. More importantly, the catalytically five-membered rhodacycle **B** was prepared, characterized and proved to be catalytically active.

### 8.3. Formation of C-heteroatom Bonds

**Scheme 85:**
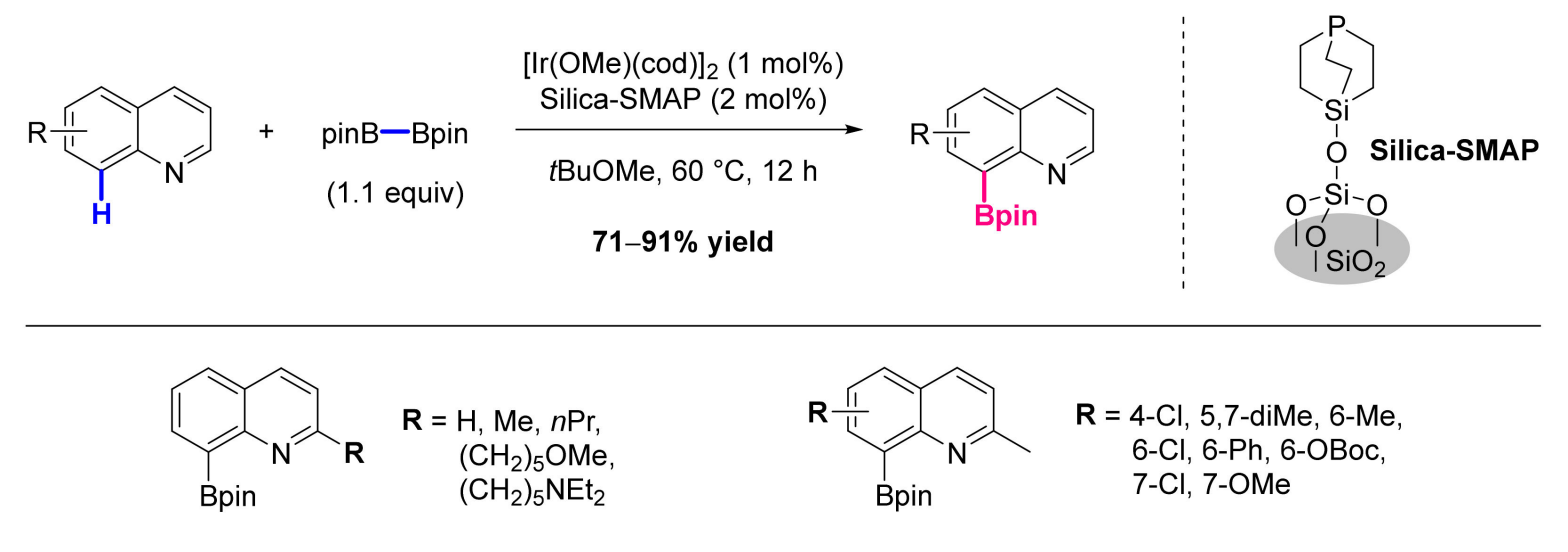
Ir-catalyzed C8 borylation of quinolines with bis(pinacolato)diboron.

In 2014, the group of Steel, Marder, and Sawamura reported the Ir-catalyzed borylation of quinolines with bis(pinacolato)diboron using a silica-supported trialkylphosphine ligand (Silica-SMAP) [172]. As above-mentioned in this review, borylation of QNs occurs preferentially at the C3 position when an Ir-bipyridine catalytic system is used [156], whereas in this case, the immobilized catalyst prepared *in situ* from [Ir(cod)(OMe)]_2_ (1 mol%) and Silica-SMAP (2 mol%) proceeds with a C8 regioselectivity. Under these conditions, several QNs were smoothly converted into the corresponding 8-quinolinylboronates in good to excellent 71–91% yields (Scheme 85). Interestingly, C8-borylated QNs proved to be useful substrates for various transformations, such as Heck and Suzuki-Miyaura cross-couplings.

**Scheme 86:**
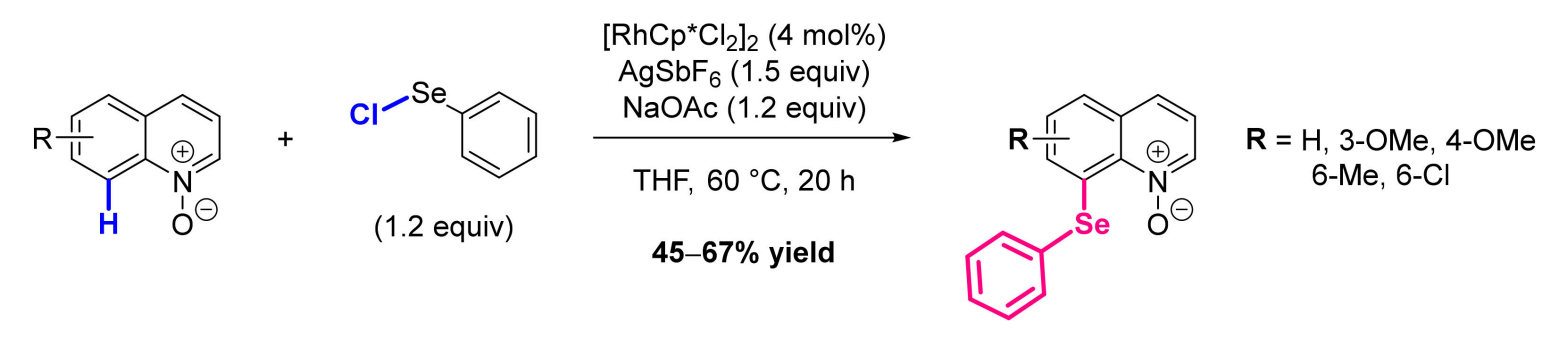
Rh-catalyzed C8 selenylation of quinoline *N*-oxides with selenyl chlorides.

In 2015, the team of Wan and Li developed the Rh-catalyzed selenylation of (hetero)arenes, including quinoline *N*-oxides, with selenyl chlorides [192]. Various additives, bases and solvents were screened against the model coupling reaction using the methyloxime derived from acetophenone (1 equiv), and phenylselenyl chloride (1.2 equiv) as substrates: the mixture of [RhCp*Cl_2_]_2_ (4 mol%), AgSbF6 (1.5 equiv) and NaOAc (1.2 equiv) in THF proved to be the most efficient. Under these optimized conditions, few QNOs were well tolerated as substrates and were converted into the corresponding C8 seleniated products in moderate to good 45–67% yields (Scheme 86).

Several experiments have been carried out using phenyl-oxime as substrate to study the reaction mechanism. The main steps of the proposed pathway are similar to those depicted above for the *path a* in Scheme 63.

**Scheme 87:**
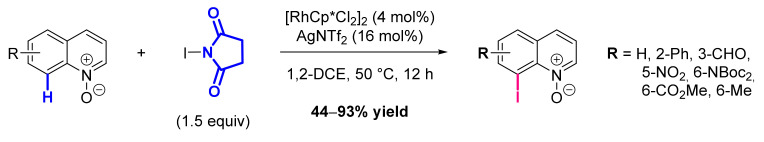
Rh-catalyzed C8 iodination of quinoline *N*-oxides with NIS.

The same year, the group of Chang developed two methods to perform the regioselective introduction of heteroatoms at the C8 position of quinoline *N*-oxides, namely a Rh-catalyzed iodination using NIS and an Ir-catalyzed sulfonamidation with aryl sulfonyl azides [193]. Concerning the first one, the iodination, after the investigation of various halogenation conditions through the screening of different catalyst/additive combinations, halogen sources and solvents, it appeared that classic chlorination and bromination agents were not synthetically useful whereas using a mixture of [RhCp*Cl_2_]_2_ (4 mol%), AgNTf2 (16 mol%) and N-iodosuccinimide (NIS, 1.5 equiv) in DCE proved to be efficient. Several QNOs were tested as substrates and were smoothly converted into the desired iodinated products in 44–93% yields (Scheme 87). Interestingly, this reaction proved to be regioselective for the C8 position even if a phenyl is present in position 2.

**Scheme 88:**
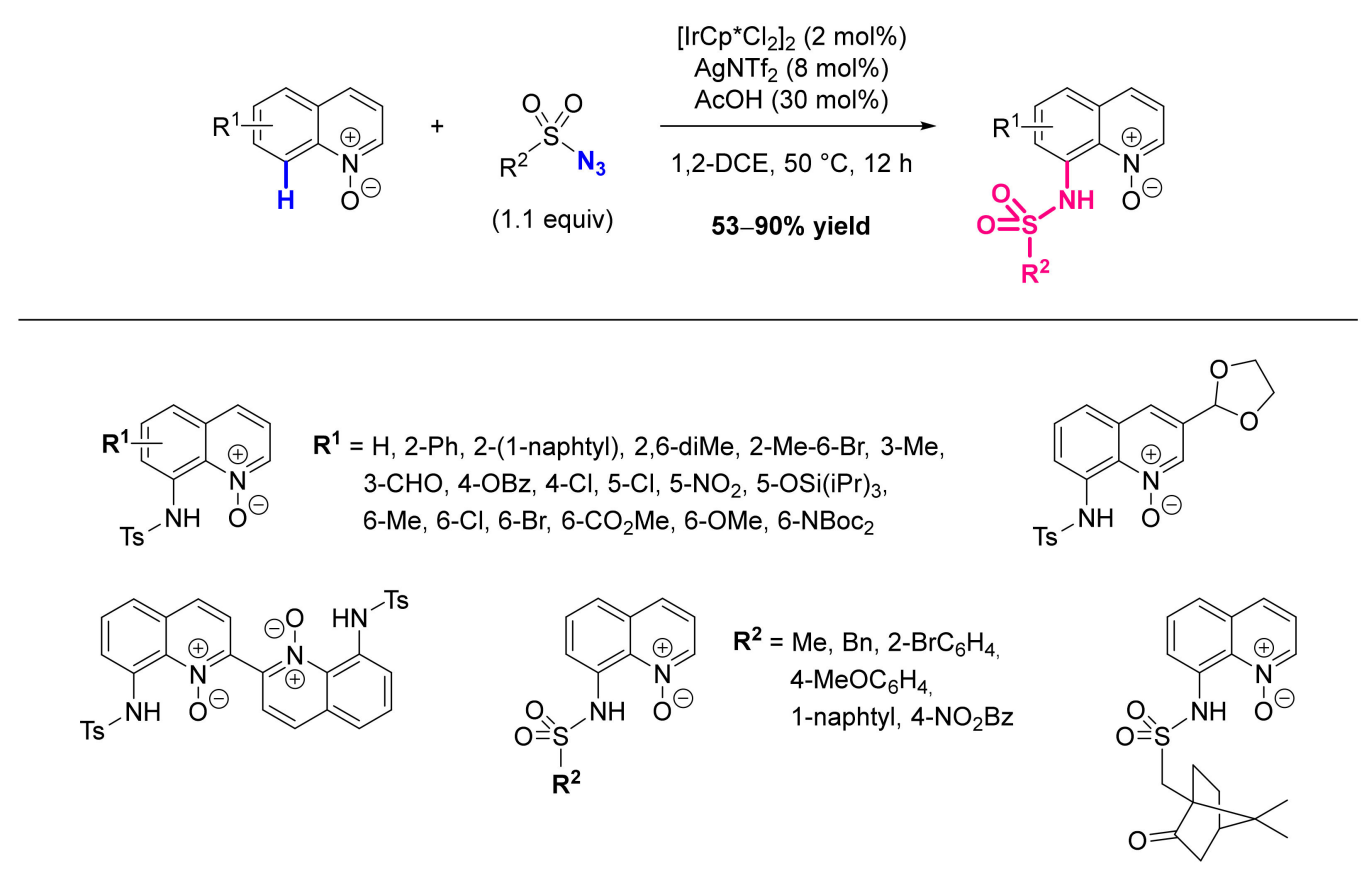
Ir-catalyzed C8 sulfonamidation of quinoline *N*-oxides with sulfonyl azides.

Next, investigation of the sulfonamidation reaction was carried out with *p*-toluenesulfonyl azide (1.1 equiv) and required in turn a full optimization of the conditions, during which the addition of acetic acid (30 mol%) and the decrease of temperature to 50 °C were found to be crucial parameters. The generality of the reaction was assessed using diversely substituted quinoline *N*-oxides with aryl or alkyl sulfonyl azides, affording a wide library of the desired C8 substituted products in good to excellent 53–90% yields (Scheme 88). This reaction exhibits a broad functional group tolerance and a very high regioselectivity.

After several control reactions in order to investigate the mechanism, the authors proposed a possible reaction pathway that matches with *path a* described above in Scheme 63, for which the key metalacycle **B** was characterized by X-ray analysis and the key intermediate **D** was detected by ESI-MS.

**Scheme 89:**
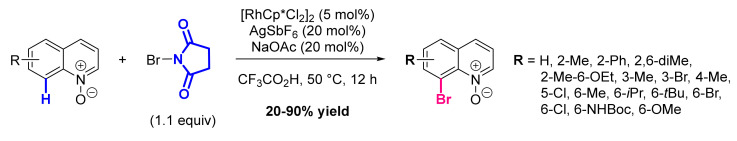
Rh-catalyzed C8 bromination of quinoline N-oxides with NBS.

Inspired by this work, the group of Sharma reported in 2019 two new Rh-based protocols to achieve the C8 regioselective formation of C-Br and C-N bonds on quinoline *N*-oxides [194]. Regarding the bromination method, various reaction parameters were studied to rapidly optimize the model reaction involving *N*-bromosuccinimide (NBS, 1.1 equiv), [RhCp*Cl_2_]_2_ (5 mol%) and AgSbF_6_ (20 mol%) in trifluoroethanol, and addition of NaOAc (20 mol%) proved to be beneficial. Exploration of the scope with various QNOs afforded the corresponding C8 brominated products in 20–90% yields, the lower value corresponding to the sterically hindered 2-phenyl substrate (Scheme 89). Besides being compatible with various functional groups, this reaction can also be performed on a gram scale.

The mechanism proposed by the authors is similar to the *path a* described above in Scheme 63, for which the key rhodacycle **B** was prepared and successfully used to catalyze the bromination.

**Scheme 90:**
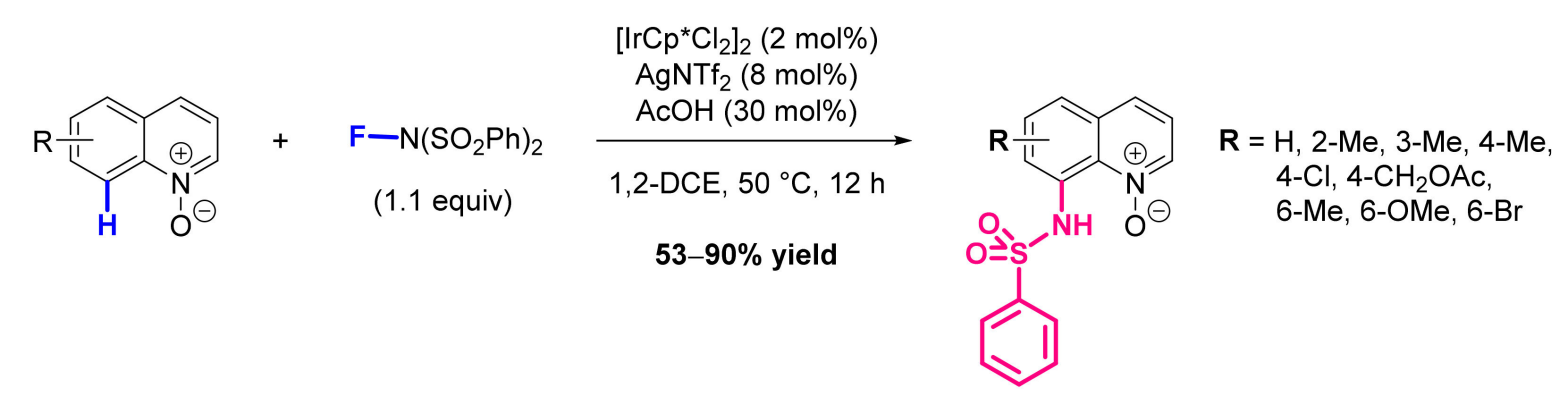
Rh-catalyzed C8 sulfonamidation of quinoline *N*-oxides with NFSI.

In the second part of their study, the C8 amidation was investigated in the exact same conditions with *N*-fluoro-bis(phenylsulfonyl)-imide (NFSI, 1.1 equiv) as a bench-stable amidating agent, and proved to require shorter time than the bromination. The scope of the reaction was assessed with a panel of QNOs which were converted smoothly into the corresponding phenylsulfonylamides in good 58–78% yields (Scheme 90). Interestingly, these conditions are also compatible with aryl sulfonyl azides, which allow the introduction of structural diversity on the phenyl ring.

## 9. Summary and Outlook

This review summarizes the tremendous work that has been achieved towards the development of transition-metal-catalyzed C-H functionalization of quinolines, which allows the formation of carbon-carbon bonds as well as carbon−heteroatom bonds, such as C-N, C-O, C-X, C-B, C-Si, C-Se. With respect to the catalyst, Pd, Cu and Rh are the most widely used along with a few examples of Ni, Ir or Co-based conditions. The vast majority of these methods targets the C2 position because of its proximity to the nitrogen atom of the pyridine ring that acts as a DG. A large number of regioselective methods have also been found to achieve the C8-H functionalization of QNOs thanks to the formation of a key five-membered metalacycle intermediate. Remote C-H activation in the other positions remains a challenge and therefore was less documented. Nonetheless, smart examples of TDG-assisted or LA-promoted methods proved to be efficient and should pave the way for the discovery of other site-selective functionalization methods at distal positions. These recent advances should facilitate the access to diversely substituted quinolines which are highly demanded in medicinal or material chemistry.

## Data Availability

Not applicable.
